# Anti-Kasha fluorescence and persistent room-temperature phosphorescence from a nearly flat non-symmetric polycyclic hydrocarbon

**DOI:** 10.1038/s41467-026-78107-6

**Published:** 2026-10-02

**Authors:** Monike da Silva Kutz, Carolina Francener, Wataru Ota, Manuela Santos Corrêa, Daniel Crane, Emiko Fujiwara, Tohru Sato, Ivan H. Bechtold, Andrew P. Monkman, Eduard Westphal, Harald Bock

**Affiliations:** 1 Department of Chemistry, Universidade Federal de Santa Catarina, Florianópolis, Brazil; 2 Centre de Recherche Paul Pascal, Université de Bordeaux/Centre National de la Recherche Scientifique, Pessac, France; 3 Department of Physics, Durham University, Durham, UK; 4 Fukui Institute for Fundamental Chemistry, Kyoto University, Kyoto, Japan; 5 Department of Molecular Engineering, Graduate School of Engineering, Kyoto University, Nishikyo-ku, Kyoto, Japan; 6 Department of Physics, Universidade Federal de Santa Catarina, Florianópolis, Brazil

**Keywords:** Excited states, Optical materials, Computational chemistry

## Abstract

Polycyclic aromatic hydrocarbons are molecular frameworks that enable the development of efficient emitters owing to their extensive conjugation and rigid structure. Here, we introduce monohomotruxene (MHTX), designed by reducing the symmetry of homotruxene (HTX) and truxene (TX). This partial planarization greatly enhances the complex photophysics over symmetric, triply twisted HTX, substantially increasing the photoluminescence quantum yield (PLQY), a result we attribute to its enhanced molecular rigidity and symmetry breaking. MHTX displays an unconventional violation of Kasha’s rule, yielding excitation-dependent dual emission. Excitation of the S_2_ state under anaerobic conditions produces intense, 455–480 nm, ultralong room-temperature phosphorescence (RTP), lasting over 12 s at room temperature. In contrast, excitation into the S_1_ state yields prompt fluorescence and weak delayed fluorescence (DF) by triplet-triplet annihilation (TTA) in the neat film. Aggregate species also present a complex emissive behavior with weak orange phosphorescence and TTA-DF. These findings establish MHTX as a promising emissive scaffold, highlighting the potential for accessing complex excited-state dynamics and advanced photophysical functionalities.

## Abstract (web summary)

Polycyclic aromatic hydrocarbons are efficient emitters, but achieving desirable properties can be challenging. Here, the authors report the development of monohomotruxene, a nonsymmetric compound with anti-kasha and ultra-long blue phosphorescence.

## Introduction

Persistent room-temperature phosphorescence (pRTP) is the long-lived emission originating from a triplet excited state that remains visible to the naked eye for a period ranging from seconds to tens of hours^1,2^. Materials that show it are useful in security printing^3,4^, information storage^5^, time-gated biological imaging^6,7^, and oxygen sensors^8^. Currently, the majority of organic pRTP emitters are heavy metal-containing molecules, where charge-transfer (CT) processes mix singlet (S_n_) and triplet (T_n_) excitons, and the strong spin–orbit coupling (SOC) improves both the rate of intersystem crossing (ISC) (k_ISC_) and phosphorescence (k_phos_)^9^. However, metal-based phosphorescent emitters, such as metal-organic complexes, are often toxic, expensive, chemically unstable, poorly soluble and difficult to process^10,11^. To overcome these limitations, recent efforts have focused on the molecular design of purely organic pRTP emitters, whose structural versatility is exploited to achieve efficient metal-free RTP^12,13^.

One common molecular design strategy to increase triplet excited state yield after photoexcitation focuses on enhancing the k_ISC_ to outcompete both the fluorescence (k_f_) and the internal conversion (k_IC_) rate^14^. This includes: (i) employing the heavy-atom effect (HAE) by incorporating halogen atoms (Cl, Br, I) to enhance SOC^15–17^; (ii) promoting charge-transfer (CT) character to satisfy El-Sayed’s rule, either by introducing carbonyl groups^18^ or heteroatoms with lone electron pairs (e.g., O, N, S); and (iii) minimizing the singlet-triplet energy gap (ΔE_ST_) using twisted donor-acceptor (D-A) architectures, which facilitates the mix of S_n_ and T_n_ states, in accordance with El Sayed’s rule^19–21^. Additionally, ISC restrictions can be lifted through spin-vibronic coupling via out-of-plane modes^22^.

Further, to achieve pRTP, the spin-forbidden nature of radiative triplet decay in heavy-atom-free and metal-free molecules is important. The primary challenge in these systems is not just enabling intersystem crossing (ISC), but outcompeting efficient non-radiative decays. This is addressed by designing rigid molecular structures that suppress higher-amplitude vibrational motions in the T_1_^23^. In planar and symmetric aromatics systems, ISC can be efficient even without heavy atoms if a higher-energy triplet state (T_n_) is close to S_n_. However, an enhancement of k_ISC_ might also lead to greater phosphorescence rate (k_phos_), diminishing its persistence. One alternative is to utilize different excited states, enabling efficient ISC through upper states while maintaining slow radiative decay between T_1_ and S_0_.

Another photophysical process with important applications is emission from multiple singlet excited states, yielding excitation-dependent multilevel emission with potential uses in sensing^24^, bioimaging^25^, and anti-counterfeiting^26^. This phenomenon represents a violation of Kasha’s rule, which states that radiative decay occurs exclusively from the lowest-energy excited electronic state^27^. However, as observed in many anti-Kasha emitters, such as azulene and pyrene, S_0_ ← S_2_ emission can occur when the rate of S_2_ → S_1_ internal conversion (IC) becomes comparable to the fluorescence rate^28^.

**Fig. 1:**
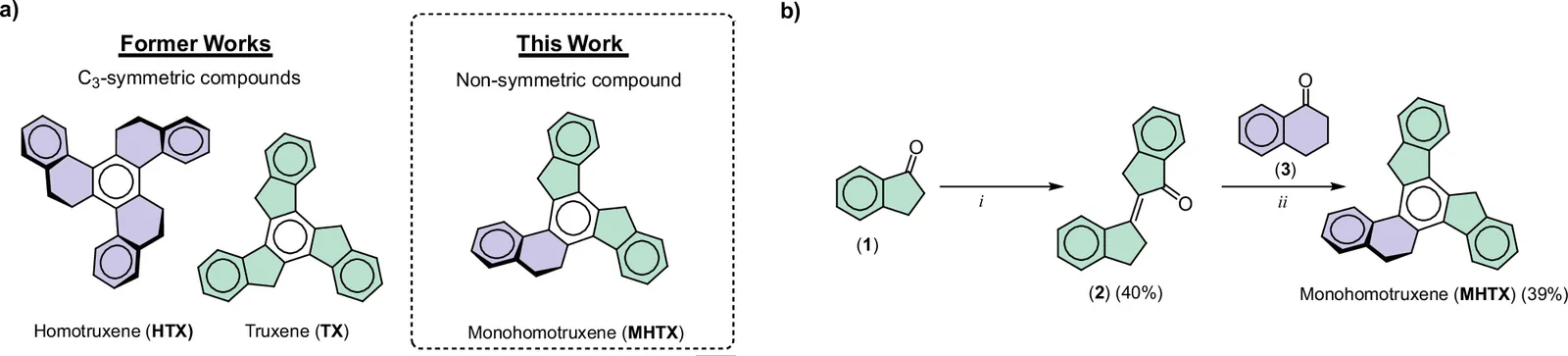
Structure and synthetic route of MHTX. **a** Molecular structures of homotruxene (**HTX**)^16,29^, truxene (**TX**)^52^ and the novel non-symmetric **MHTX** reported in this work. **b** Synthetic route to **MHTX**: (*i*) TiCl_4_, triethylamine, dichloromethane, hexane, 16 h at room temperature under argon; (*ii) para*-toluenesulfonic acid, propionic acid, *ortho*-dichlorobenzene, 24 h at 140 °C under argon in the dark.

Homotruxene (**HTX**; Fig. 1a), a non-planar C_3_-symmetric twisted polycyclic aromatic hydrocarbon (PAH), displays blue delayed fluorescence (DF) by triplet-triplet annihilation (TTA) and persistent RTP from aggregate species. The orange aggregate RTP, whose phosphorescence lifetime (τ_phos_) was determined to be 0.38 s, can be easily observed by the naked eye when **HTX** powder is excited at 365 nm^29^. High triplet-state generation in **HTX** is due to an efficient ISC promoted by out of plane vibronic coupling SOC from torsional strain between the molecular orbitals. As the initial (π) and final excited states (π*) are twisted, the change in orbital angular momentum upon excitation, or de-excitation, results in a non-zero spin-orbit coupling matrix element (SOCME), enabling triplet-state population^16,21^. However, truxene (**TX**; Fig. 1a), the planar analogue, does not show any aggregate RTP in the solid state when excited at 365 nm^29^.

In this work, we demonstrate how enhancing molecular planarity and rigidity, and reducing the symmetry from **HTX** can preserve TTA and RTP, enhance the photoluminescence quantum yield (PLQY) and result in complex photophysical behavior, with radiative decay from upper excited states. For this, we replaced two of the three flexible non-planar six-membered rings in **HTX** with planar five-membered rings, resulting in a non-symmetric nearly-flat polycyclic aromatic hydrocarbon (PAH), dubbed monohomotruxene (**MHTX**; Fig. 1a). This modification was designed to reduce non-radiative vibrational decay by increasing rigidity, and to verify the limits of planarization of **HTX** to retain its optical properties. Furthermore, by leveraging the innate torsion of the homotruxene core, we aimed to enhance spin-orbit coupling (SOC) without introducing heteroatoms for lone-pair donation. In this case, SOC could be promoted via out-of plane vibrational coupling, but also, as in coronene, through vibrational mixing of (π,π*) and (σ, π*) orbitals^30^. This mechanism can give rise to efficient ISC and radiative decay of the triplet to the ground state without the need for coupling to high energy vibronic modes. Quenching of these modes by molecular rigidity would greatly reduce non-radiative processes such as internal conversion.

## Results

### Synthesis

**MHTX** was synthesized via a two-step route to minimize the formation of yield-compromising byproducts from the direct reaction of 1-indanone (**1**) and 1-tetralone (**3**) (Fig. 1b). The first step involved the preparation and selective isolation of the α,β-unsaturated dimer (**2**) of the aldol self-condensation of 1-indanone (**1**) in the presence of titanium tetrachloride and triethylamine^31^. Subsequently, the purified dimer underwent a Brønsted acid-catalyzed aldol condensation under an argon atmosphere and in the absence of light with a four-fold molar excess of 1-tetralone (**3**) to afford **MHTX**^32^. Optimization studies (Table S1) identified as the most efficient conditions an equimolar combination of anhydrous *para*-toluenesulfonic acid (PTSA) and propionic acid (1.75 molar equivalents of each relative to **2**) as catalysts. Synthetic procedures for **MHTX,** **TX**, and **HTX**, alongside reaction optimization data and full structural characterization, are provided in the Supplementary Information.

### Anti-Kasha fluorescence of MHTX

**Fig. 2:**
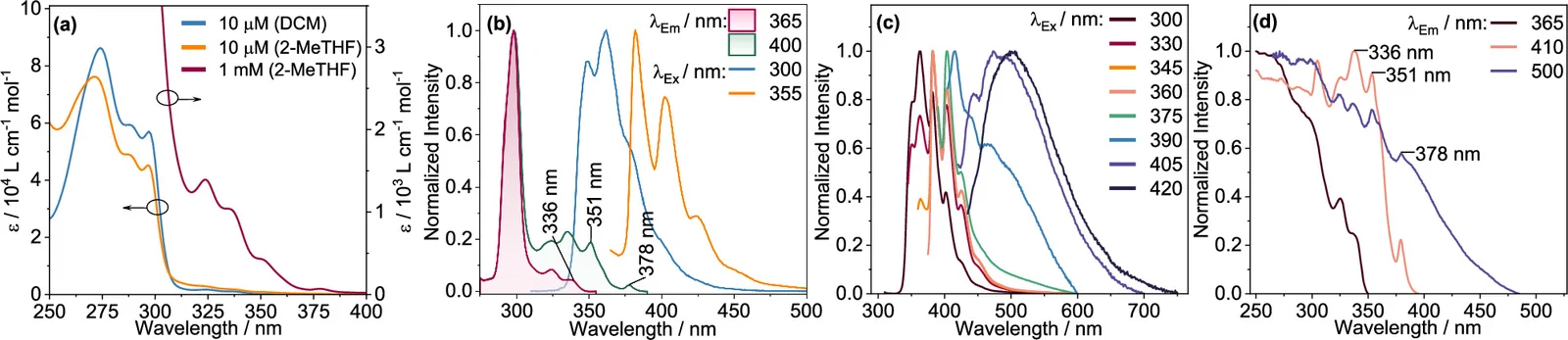
Steady-state photoluminescence and excitation profiles of MHTX at room temperature. **a** Absorption spectra of **MHTX** in 10 μM solutions in dichloromethane, DCM(blue) and 2-methyltetrahydrofuran, 2-MeTHF (orange), and 1 mM solution in 2-MeTHF (dark red). **b** Normalized excitation and emission spectra in 2-MeTHF (10 μM). **c** Normalized emission spectra of a 1 wt.% drop-cast film in Zeonex under different excitation wavelengths in the presence of molecular oxygen. **d** Normalized excitation spectra under the same conditions as Figure (**c**).

The absorption spectra were recorded in different solvents (Fig. 2a and Table S2). In 2-methyltetrahydrofuran (2-MeTHF; 10 µM), **MHTX** exhibits a strong absorption band with a maximum at 271 nm (ε = 7.6 × 10^4 ^L cm^−1^ mol^−1^) and shoulder-like features at 288 and 297 nm. In DCM (10 µM), the maximum is slightly red-shifted to 274 nm, while the shoulders remain at the same wavelengths. Moreover, the molar absorptivity in DCM is somewhat higher than in 2-MeTHF. Weaker bands (ε < 1.4 × 10^3^ L cm^−1^ mol^−1^) are observed in the 315 – 380 nm range in both solvents. Upon increasing the concentration (1 mM) and in the solid state (1 wt.% in Zeonex drop-cast film; Figure S11a), the weak bands become prominent, with distinct maxima at 325, 336, 351, and 378 nm. This spectral profile is identical to the analogous C_3_-symmetric compounds **HTX** (Figure S11b) and **TX** (Figure S11c)^29,33^, where the intense bands are assigned to the symmetry-allowed S_0_ → S_3_ and S_0_ → S_4_ transitions, and the lower-intensity bands to the symmetry-allowed S_0_ → S_2_ transition and symmetry-forbidden S_0_ → S_1_ transition, as described in the theory section. Therefore, the degree of symmetry breaking in **MHTX** is insufficient to completely lift the symmetry selection rule, as the first two electronic transitions remain largely forbidden in character.

Unexpectedly, steady-state photoluminescence measurements reveal anti-Kasha emissive behavior for **MHTX**, as very weakly observed in coronene^34^. In solution, excitation at the edge of the strong absorption region (λ_Ex_ = 300 nm) yields a vibrationally structured emission band with maxima at 350 and 360 nm and shoulders at 380, 400 and 425 nm (Fig. 2b, blue line; and S12a). These spectral features persist across varying concentrations (Fig. S12b) and solvent polarities (Fig. S12c, solid lines), confirming their locally excited (LE) character. In contrast, excitation at 355 nm results in a red‑shifted, still vibronically structured emission band, with maxima at 384, 404 and 425 nm (Fig. 2b, orange line; and S12a). While this band overlaps with the shoulder features observed under 300 nm excitation, their maxima are not coincident, suggesting they originate from distinct electronic levels, which will subsequently be assigned to S_0_←S_2_ and S_0_←S_1_ transitions, respectively. Furthermore, the vibrational resolution and the absence of a solvatochromic shift across different polarities confirm the LE character of this transition (Figure S12c, dashed lines). Integrated photoluminescence intensity is enhanced 2.4-fold and 3.8-fold for S_0_←S_1_ and S_0_←S_2_ transitions, respectively, upon deaeration (Figure S12a), due to the decrease of singlet quenching by oxygen (See SI section 4.1 and Table S3). To elucidate the origin of the dual prompt fluorescence (PF), excitation spectra of the two emission bands were recorded. When monitoring the redder emission (λ_Em_ = 400 nm; Fig. 2b), two primary excitation bands emerge at longer wavelengths, with maxima at 351 nm and 378 nm. They are assigned to the S_1_ state. Conversely, monitoring emission at 365 nm, the resulting excitation profile shows a sharp cut-off at 350 nm and a primary maximum at 336 nm, effectively excluding excitation from the S_1_ state. This divergence in the excitation profiles in dilute solutions provides evidence for an anti-Kasha behavior, where the strong higher-energy emission is assigned to S_0_←S_2_ decay, while the lower-energy emission is identified as the S_0_←S_1_ transition.

To exclude that this dual-PF emission arises from the coexistence of monomeric and dimeric species, an antisolvent experiment was performed in THF-water mixtures with a constant total concentration of 20 µM (Supplementary Information section 4.2). The dual-emission remained unchanged at f_w_ ≤ 60%, indicating a strong THF–**MHTX** interaction that forms a protective solvation shell, effectively hindering molecular aggregation despite the increasing presence of water. In contrast, at f_w_ ≥ 70%, the S_2_ emission strongly decreases, and an emission band similar to S_1_ emission appears when exciting at 300 nm (into S_2_), however the peaks are red-shifted by 4 nm (0.03 eV). Exciting into S_1_ (355 nm) gives very weak S_1_ emission from 0 to 70% water content, followed by a 4 nm (0.03 eV) red-shift of the band at higher water fractions. This coincides perfectly with the emergence of the strong S_1_ emission and decline of S_2_ emission when exciting at 300 nm. We suggest that this red-shifted band emerges due to the formation of a weak intermolecular dimer, designated as “loose dimer” as the water content starts to overcome the stabilizing THF solvation shell, where the molecular orbitals remain predominantly localized on individual molecules and intermolecular interactions are weak. The sustained spectral profile with low to moderate f_w_ confirms that both the S_2_ and S_1_ bands are intrinsic to the monomeric species, rather than being products of ground-state association dimer or excimer formation. Both assignments are further supported by the DFT calculations discussed in the following sections. At f_w_ > 50%, an additional band starts to grow 525 nm, attributed to emission from disordered aggregate states, formed as **MHTX** drops out of solution at high water content.

The anti-Kasha emission is preserved in the drop-cast film (1 wt.% in Zeonex; Fig. 2c, d), showing a marked contrast to previously reported systems where S_2_ emission is restricted to solution^35^. In the film, we observe an intensification of the S_1_ emission relative to the S_2_ band under high-energy excitation (λ_Ex_ = 300 and 330 nm; Fig. 2c). This is attributed to the rigidifying effect of the Zeonex matrix, which suppresses large-amplitude molecular vibrations and restricts the non-radiative dissipation prevalent in fluid media. This structural confinement is further supported by the prompt fluorescence lifetime, determined via time-correlated single photon counting (TCSPC) in the solid state (Fig. S17 and Table S4), yielding values of 30.6 ns and 42.9 ns for S_2_ and S_1_, respectively. The S_1_ decay in the solid state is approximately 10 ns longer than in degassed solution (32.9 ns; Table S3), while the τ_PF_ for S_2_ remains nearly identical (32.9 ns, Table S3), confirming the higher sensitivity of S_1_ to vibrational suppression by the polymer matrix. Excitation at longer wavelengths (λ_Ex_ = 390, 405 and 420 nm; Fig. 2c) leads to a red-shifted, broad emission, dominated by the decay of strongly coupled aggregates, herein called “strong dimer”. A broad, low-energy excitation band associated with this non-monomeric species appears around 400 nm (λ_Em_ = 500 nm, Fig. 2d), confirming that it has a ground state and is, indeed, an aggregate and not an excimer. Additionally, sharp excitation features associated with S_0_ → S_1_ (351 and 378 nm) and S_0_ → S_2_ (336 nm) are clearly present, as in the absorption spectra in solution, indicating energy transfer from monomeric excited states to the aggregate, likely through enhanced vibronic coupling assisted internal conversion to the aggregate intermediate state (Fig. S30c). The rigidity of **MHTX** minimizes conformational reorganization upon photoexcitation, with small Stokes shifts (Δν) of 0.15 eV and 0.05 eV for the S_2_ and S_1_ transitions (Table S2); a spectroscopic signature of constrained geometries. The PLQY of **MHTX** in degassed solution (20 µM in 2-MeTHF) is higher than that of its analogous structures under identical conditions (λ_Ex_ = 310 and 355 nm): **MHTX** achieves PLQYs of 57% and 32% at these respective wavelengths, significantly outperforming **HTX** (32%/21%) and **TX** (14%/1.8%). This enhancement is attributed to the increased structural rigidity of **MHTX** relative to **HTX** and the breaking of C_3h_-symmetry relative to **TX**. The latter effectively mitigates the symmetry-forbidden nature of the S_1_ → S_0_ transition, thereby enhancing radiative rates^36–39^. This improved performance extends to the solid state (1 wt.% in Zeonex), where the PLQY reaches 49% (λ_Ex_ = 310 nm) and 63% (λ_Ex_ = 355 nm). **MHTX** outperforms **HTX** (32%, λ_Ex_ = 355 nm) and **TX** (35%, λ_Ex_ = 355 nm) even in the condensed phase, underscoring the efficacy of our molecular design in modulating the excited-state landscape to favor radiative transitions across multiple singlet states.

### Dual delayed emissions of MHTX

**Fig. 3:**
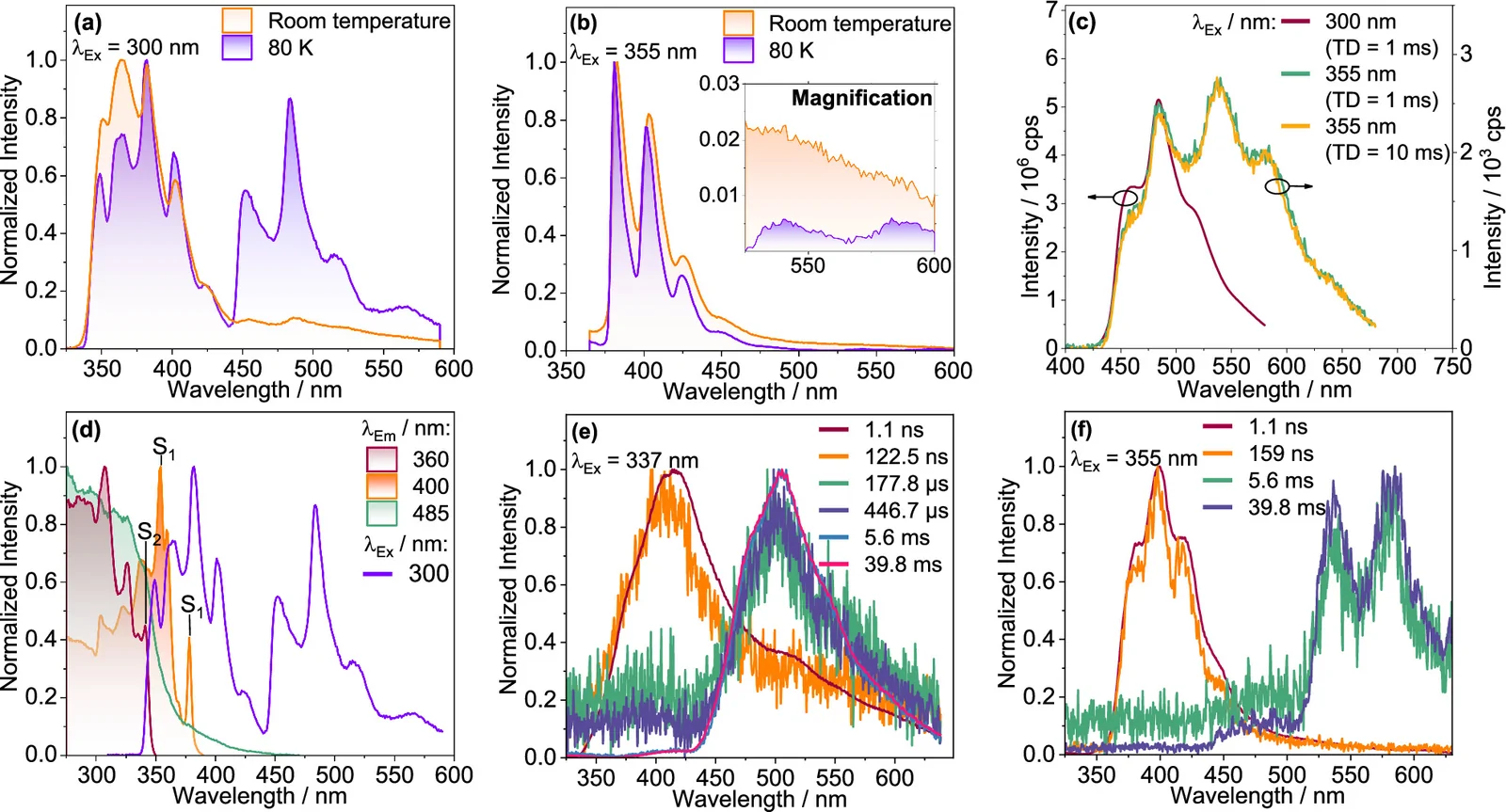
Temperature- and time-dependent photoluminescence of MHTX. **a**,**b** Normalized steady-state emission of **MHTX** (1 wt.% in Zeonex) at room temperature (orange area) and 80 K (purple area) upon excitation at **a** 300 nm and **b** 355 nm. **c** Emission spectra at different time delays upon excitation at 300 nm (red line, left y-axis) and 355 nm (orange and green lines, right y-axis) at 80 K. **d** Combined emission and excitation spectra recorded at different emission wavelengths (λ_Em_), highlighting the exclusion of S_1_ vibrational bands (labeled) when monitoring high-energy phosphorescence. **e**,**f** Time-gated emission spectra at 80 K recorded at the indicated time delays following excitation at **e** 337 nm (0.5 wt.% in Zeonex) and **f** 355 nm (0.1 wt.% in Zeonex).

In addition to the dual PF observed in the UV-to-blue region upon excitation to S_2_, **MHTX** displays a distinct blue emission band under vacuum, with vibrational peaks at 452, 485, and 515 nm and a shoulder around 565 nm (Fig. 3a). The marked increase in intensity of this emission at 80 K, coupled with its complete quenching under air at room temperature (Fig. S18a), suggests a triplet state-mediated mechanism, which we attribute to phosphorescence from monomeric species, a feature that is preserved in the neat film (Fig. S18b). This blue phosphorescence is consistent with previously reported triplet emissions for **TX** and other truxene-derived compounds^39,40^. In contrast, direct excitation of the S_1_ state (λ_Ex_ = 355 nm) yields a very weak, red-shifted orange emission with maxima at 545 and 595 nm, appearing alongside S_1_ monomeric PF (Fig. 3b). This orange component is attributed to phosphorescence from aggregates, a conclusion substantiated by its higher intensity in the neat film compared to a dilute sample (Fig. S18c).

The blue phosphorescence is extremely weak upon S_1_ excitation, compared to excitation to S_2_ (Fig. 3c; λ_Ex_ = 355 and 300 nm, respectively), indicating an inefficient ISC from S_1_ due to weak SOC between S_1_ and an isoenergetic triplet state. This divergence suggests that S_2_ serves as the primary gateway for population of monomeric triplets. To confirm the negligible contribution of S_1_ to the monomeric triplet population, excitation spectra were collected at 80 K while monitoring the monomeric phosphorescence at 485 nm (Fig. 3d, green shaded) and compared to the S_2_ and S_1_ excitation profiles (Fig. 3d, red and orange shaded). The phosphorescence excitation spectrum excludes the sharp transition regions corresponding to the S_1_ state. This selective phosphorescence excitation profile confirms that intersystem crossing is more efficient from the higher-energy S_2_ state than from the S_1,_. potentially indicating that (π,π*) (σ, π*) coupling drives the ISC.

Time-gated photoluminescence measurements on a 1 wt.% Zeonex drop-cast film at 80 K confirm the long-lived nature of the cyan emission relative to the PF (λ_Ex_ = 337 nm, Fig. 3e). While the PF is observable at early TDs of 1.1 to 122.5 ns, the delayed emission becomes predominant at a delay of 178 μs and persists through the experimental detection limit (40 ms). The phosphorescence spectral profile overlaps with the strongly coupled PF from aggregates centered at approximately 500 nm, in the nanosecond regime. Conversely, excitation to S_1_ (λ_Ex_ = 355 nm, Fig. 3f) yields the weaker combination of monomeric and aggregate phosphorescence that emerges only after longer TDs (5.6 ms). This kinetic behavior is consistent with the 1 wt.% drop-cast film at room temperature (Fig. S20 and Table S3), frozen solution (Fig. S21), and neat films at both room temperature and 80 K (Fig. S22).

To verify the contribution of TTA to the delayed emissions of **MHTX**, as previously reported for C_3_-symmetric **HTX**^29^, power-dependent photoluminescence analyses were performed on a drop-cast 1-wt.%-in-Zeonex film under vacuum. At both room temperature and 80 K (Fig. S23a–d), the emission intensity exhibits a non-linear dependence on the excitation dose, with a slope of 2 that changes to 1.1 in the high-power regime. This kinetic behavior is characteristic of a bimolecular TTA process^41,42^ and confirms that at the early stage, delayed emission is dominated by TTA-DF within the strong aggregates. In contrast, in frozen dilute solution (Fig. S23e–f), the slope was found to be 0.9, indicating the dominance of monomolecular phosphorescence under dilute conditions, where the spatial separation of triplet states precludes the bimolecular interactions required for TTA. Lifetime of aggregate TTA at room temperature is 213.2 μs (Fig. S17c). A 1 wt.% Zeonex film of **TX** exhibited a consistent power-dependence slope of approximately 1.0 across the entire excitation range at room temperature, confirming that its delayed emission is governed exclusively by long-lived RTP^40^ (Fig. S24).

**Fig. 4:**
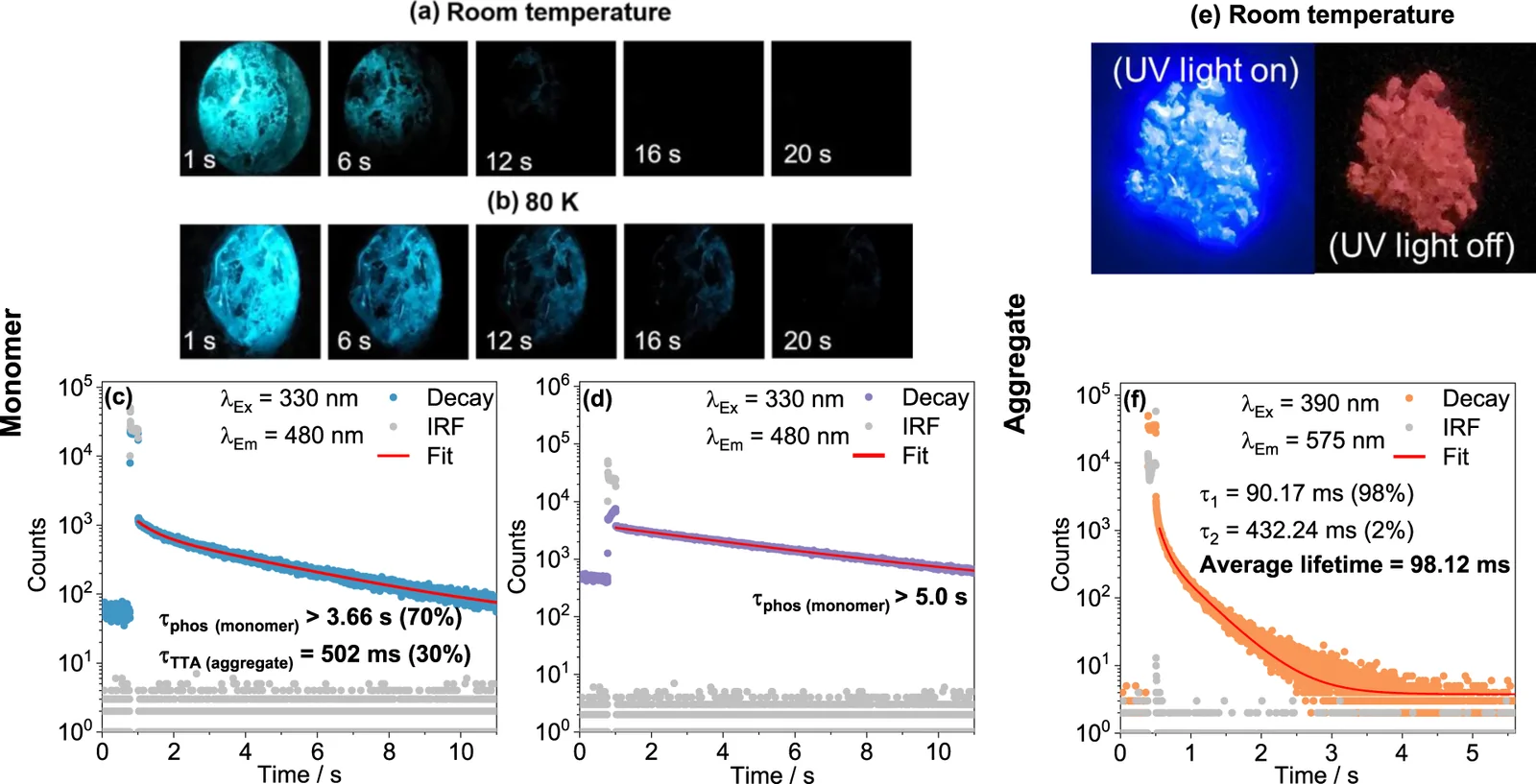
Long-lived luminescence of MHTX. **a**,**b** Photos showing the persistent cyan luminescence of a film of 1 wt.% **MHTX** in Zeonex after the removal of the excitation source (λ_Ex_ = 337 nm) at **a** room temperature and **b** 80 K. **c**,**d** Time-resolved luminescence decay profiles recorded via time-resolved single photon counting (11 s time window) for **c** 1 wt.% **MHTX** in Zeonex at 80 K and **d** a 10 µM solution of **MHTX** in 2-methyltetrahydrofuran at 80 K (λ_Ex_ = 330 nm, λ_Em_ = 480 nm). The red solid lines are numerical fits and the gray dots are the instrument response function (IRF). **e** Photos of **MHTX** powder at room temperature under UV irradiation (λ_Ex_ = 365 nm, left) and after the excitation source is turned off (right), showing the orange luminescence. **f** Time-resolved luminescence decay profile of the aggregate emission recorded for 1 wt.% **MHTX** in Zeonex (λ_Ex_ = 390 nm, λ_Em_ = 575 nm). The red solid line is the numerical fit and the gray dots are the instrument response function (IRF).

The delayed emission of **MHTX** is long-lived; upon excitation to the S_2_ state (λ_Ex_ = 337 nm), a cyan emission remains visible to the naked eye for more than 12 s at room temperature (Fig. 4a) and extends to 20 s at 80 K (Fig. 4b). To our knowledge, this is amongst the longest blue phosphorescence lifetimes observed with PAHs. TCSPC measurements at 80 K with an 11 s time window on the 1 wt.% Zeonex film reveal a biexponential decay (Fig. 4c). The primary component (τ_phos_ > 3.66 s) accounts for 70% of the total emission and is assigned to ultra-long phosphorescence, while the secondary component (τ_TTA_ = 502 ms), contributing 30%, is attributed to TTA-DF. In contrast, the frozen dilute solution (10 μM in 2-MeTHF; Fig. 4d) shows a monoexponential decay with τ_phos_ > 5.0 s. Due to the exceptionally long-lived emission, which prevents the acquisition of a complete decay profile within the experimental window, these lifetime values represent lower-end estimates rather than absolute determinations. The orange aggregate phosphorescence from a powder sample is also long-lived, remaining visible to the naked eye after the UV excitation (λ_Ex_ = 365 nm) is turned off (Fig. 4e). This emission persists even in the presence of oxygen, indicating low oxygen permeability of the powder. TCSPC measurements of a 1 wt.% drop-cast film under vacuum at 80 K reveal a lifetime of 98.1 ms for this aggregate emission (Fig. 4f). This value is shorter than both the monomeric phosphorescence and the aggregate TTA-DF (τTTA = 502 ms), suggesting efficient triplet-triplet interactions via enhanced triplet migration within the aggregate species.

### Theoretical investigations

To confirm the proposed excited states for the many observed emissions, DFT calculations were performed. The geometry of the S_0_ state was first optimized to assign the photon absorption states. The calculated UV/Vis absorption spectrum shows that the experimentally observed intense absorption band with a maximum at 271 nm (Fig. 2a) corresponds to the S_0_ → S_4_, S_0_ → S_3_, and S_0_ → S_2_ transitions, with large oscillator strengths (*f* > 0.3500, Fig. S25 and Table S5). The weak absorption feature between 315 and 355 nm arises from the symmetry‑forbidden S_0_ → S_1_ transition (*f* = 0.0012). The low oscillator strength of this transition reflects its symmetry‑forbidden character, a behavior also observed in the parent *C*_*3*_-symmetric **TX**, where the S_0_ → S_2_ and S_0_ → S_1_ transitions are symmetry-forbidden (*f* = 0.0000)^40^. This demonstrates that the dissymmetry of **MHTX** allows the weak S_1_ transition (see Fig. S26 for the molecular orbital degeneracy). Photoexcitation at 355 nm and 300 nm is therefore considered to populate S_1_ and S_2_, respectively.

**Fig. 5:**
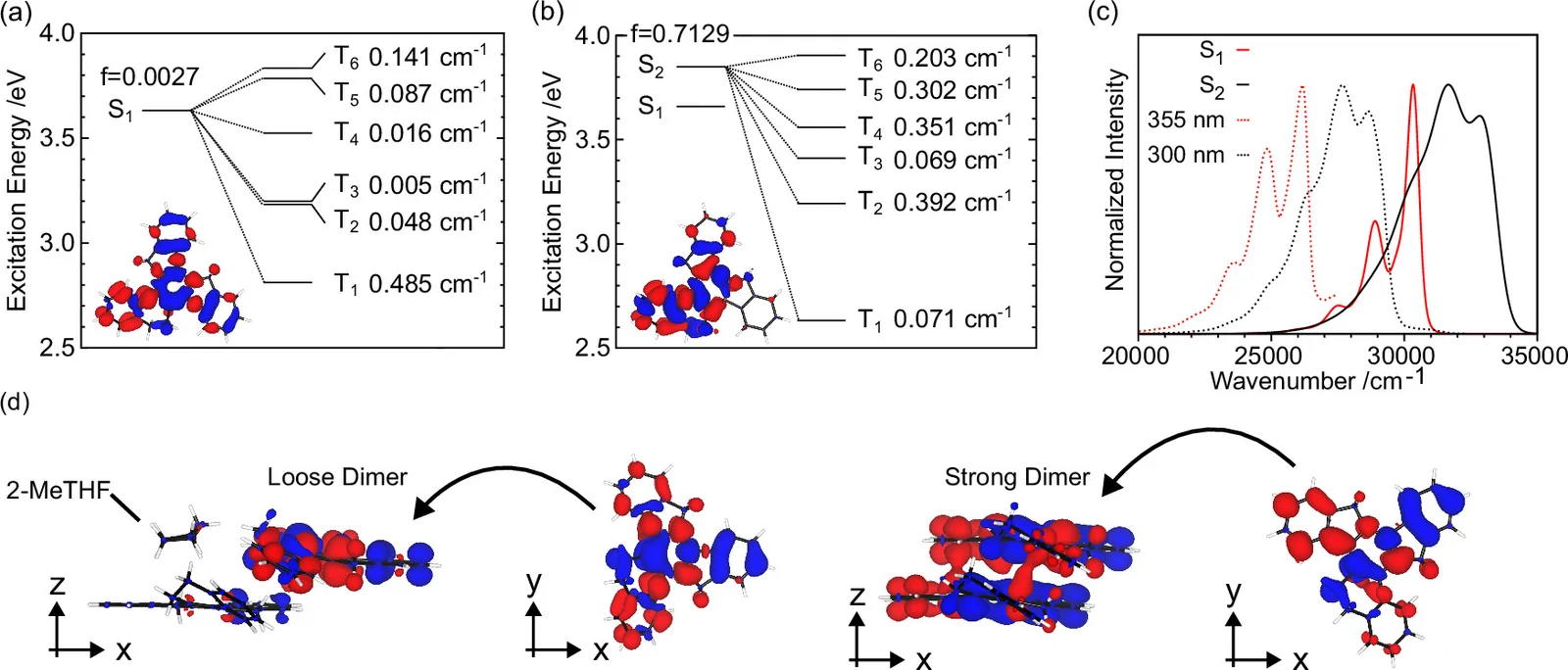
Theoretical calculation on the monomeric and aggregated properties of MHTX. Energy levels of excited states at the **a** S_1_ and **b** S_2_ optimized structures. Inset shows the electron density difference of S_1_-S_0_ and S_2_-S_0_ (isosurface value: 5 × 10^−4^ a. u.). Red and blue regions indicate positive and negative values. Values represent the spin-orbit coupling (SOC) matrix element between singlets and triplets. *f* represents the oscillator strength of S_1_-S_0_ and S_2_-S_0_. **c** Calculated S_1_ and S_2_ fluorescence spectra (solid lines), compared with the experimental spectra under 355 nm and 300 nm excitations (Fig. 2b). The density of final vibronic states is expressed using the Gaussian function with a linewidth of 100 cm^−1^. **d** Electron density difference between S_1_ and S_0_ at the S_0_ optimized structure for the loose and strong dimers (isosurface value: 5 × 10^−4^ a. u.). (a. u. = atomic units).

The S_1_ and S_2_ states were geometry-optimized (Fig. 5a, b) to investigate the relaxation processes following photon absorption. The calculated fluorescence spectra from S_1_ and S_2_, which have different electronic wavefunction distributions, reproduce the experimental spectral lineshapes observed for 355 and 300 nm excitations, respectively (Fig. 5c), suggesting that the emissions originate from distinct excited electronic states. The calculated spectral wavelengths differ from the experimental values because the excitation energies are overestimated, as is common in DFT calculations. The reorganization energy of S_0_@S_2_ → S_0_@S_0_ (0.27 eV) is larger than that of S_0_@S_1_ → S_0_@S_0_ (0.14 eV). In other words, S_0_@S_2_ exhibits stronger vibronic coupling than S_0_@S_1_, leading to the complex vibronic progression of S_2_ fluorescence (Fig. S27a, b). The vibrational mode responsible for the vibronic progression of both S_1_ and S_2_ fluorescence is the C=C stretching vibration with a frequency of around 1650 cm^−1^ (Fig. S27c, d). The potential energy curves connecting the S_1_ and S_2_ geometries show that the S_1_/S_2_ crossing point is geometrically separated from the S_2_ optimized geometry (Fig. S28). This spatial separation, together with an activation energy of 0.17 eV, may partially hinder S_2_→S_1_ internal conversion. At the S_1_ optimized structure, S_1_ and its energetically nearby triplets, particularly T_4_, exhibit weak SOC (0.02 cm^−1^, Fig. 5a), reflecting that their electronic wavefunctions are similar (El-Sayed’s rule^43^) and show small distribution over the non-planar six-membered ring (Fig. S29a). In contrast, the SOC between S_2_ and its energetically close T_5_ is strong (0.30 cm^−1^) at the S_2_ optimized structure (Fig. 5b), attributed to the differences in their wavefunction spatial distributions (Fig. S29b). Also, the distribution of the S_2_ wavefunction over the non-planar six-membered ring induces the σ and π orbitals mixing, thereby contributing to the strong SOC. These results suggest that S_2_ excitation enhances triplet production and therefore phosphorescence, compared to S_1_ excitation, through fast ISC from S_2_ to triplet states, as shown by the experiments.

To understand the emission from the aggregates, the excited electronic structures of loose and strong dimers were calculated. The loose and strong dimers were modeled using the **MHTX** dimer with and without explicit consideration of 2-MeTHF, respectively (Fig. S30a, b). At the S_1_ optimized geometry, the presence of 2-MeTHF induces J-type aggregation of **MHTX** with large intermolecular distances. Therefore, the electronic wavefunction is mostly localized on one monomer, with a small distribution on the other, leading to a slight redshift and broadening of the fluorescence spectrum compared to the monomer (Fig. 5d and Fig. S30c–e). In contrast, in the absence of 2-MeTHF, strong intermolecular interactions induce dimer formation with a delocalized excited-state electronic wavefunction, resulting in a further red-shifted and broadened fluorescence spectrum (Fig. 5d and Fig. S30c–e). Thus, increasing the water fraction in the solvent, as examined by the anti-solvent experiment, promotes the progression from the loose to strong dimer formation.

The T_1_ states of the monomer and strong dimer were geometry-optimized to investigate their phosphorescence properties. For the monomer, T_1_ exhibits a strong SOC with S_3_ (0.58 cm^−1^) which has a large oscillator strength (*f* = 0.8650, Fig. S31a, b). Therefore, phosphorescence from T_1_ is considered to occur via intensity borrowing from the S_3_-S_0_ transition dipole moment. The calculated phosphorescence spectrum reproduces the experimental spectral lineshape observed on 355 nm excitation (Figure S31c). The reorganization energy of S_0_@T_1_ → S_0_@S_0_ is 0.33 eV. S_0_@T_1_ provides strong vibronic coupling for several vibrational modes, particularly the C = C stretching mode with a frequency of 1559 cm^−1^ (Figure S31d), resulting in intricate vibronic spectral patterns. For the strong dimer, the electronic wavefunction is primarily localized on one monomer, with slight delocalization onto the other (Fig. S31e, f).

## Discussion

**Fig. 6:**
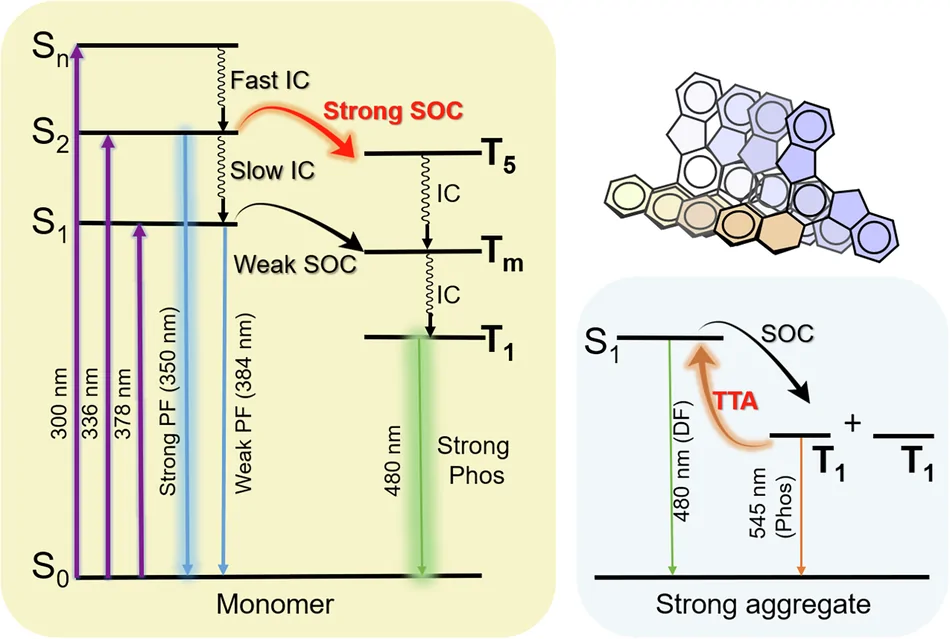
Summary of the excited-state mechanisms of MHTX. Simplified Jablonski diagram summarizing the rich photophysics of monomeric and aggregate species of **MHTX**.

The observation of dual ultra‑long delayed emissions, visible to the naked eye, from a small non-symmetric twisted hydrocarbon represents an achievement in molecular photophysics. This underscores the effectiveness of our molecular design in promoting three key outcomes: the formation of dual-emissive species (monomers and aggregates), efficient triplet harvesting, and exceptionally long‑lived emissions. The decay mechanisms for the multiple emissions of **MHTX** are summarized in Fig. 6. Upon excitation to the S_2_ state, the monomeric species preferentially emit directly from the S_2_ level at 350 nm, in a clear example of anti-Kasha prompt fluorescence. The fraction that effectively relaxes to S_1_ emits at 384 nm. The PLQY on exciting into S_2_ is much higher than that from exciting into S_1_, indicating slow IC from S_2_→S_1_. Concurrently, spin-orbit coupling facilitates intersystem crossing from S_2_ to T_5_, which subsequently populates the long-lived monomeric T_1_ state via fast internal conversion between the triplet states. This process leads to a combination of TTA from aggregates and long-lived delayed RTP at 484 nm. Persistent phosphorescence linked to dual fluorescence holds potential for the application in the development of anti-counterfeiting techniques^44^. On the other hand, direct monomeric S_1_ excitation yields weak monomeric and predominant aggregate phosphorescence due to poor spin-orbit coupling between S_1_ and its isoenergetic triplet state.

In summary, we have demonstrated that reducing the structural symmetry of a homotruxene core from three twisted arms to one in monohomotruxene maintained the complex photophysical properties of **HTX** gave a significant enhancement of photoluminescence quantum yields and phosphorescence lifetimes. The photophysical behavior of **MHTX** is rich and characterized by a strong violation of Kasha’s rule due to an inefficient internal conversion between S_2_ and S_1_ states. In addition, upon excitation to the S_2_ state, the monomeric species phosphoresces in the cyan-colored spectral region, where an efficient intersystem crossing from an upper excited state (S_2_ → T_5_) leads to an ultralong-lived high-energy triplet state. This results in intense phosphorescence, visible to the naked-eye persisting for over 12 s at room temperature (298 K) and 20 s at 80 K. Conversely, excitation to the S_1_ state selectively probes the S_1_ prompt fluorescence monomeric emission and TTA in the neat film. Aggregate species of **MHTX** phosphoresces in the orange region and emits by TTA in the cyan region. Overall, this work establishes the non-symmetric monohomotruxene core as a highly promising scaffold. The insights gained into its complex, tunable decay pathways provide a fundamental blueprint for the development of new non-symmetric PAHs with tailored optoelectronic properties.

## Methods

### Photophysical measurements

Absorption spectra in solution were recorded on a Shimadzu 2600i spectrophotometer. Steady-state spectra were acquired for solutions (aerated and degassed) and drop-cast films using Jobin Yvon Horiba Fluorolog and Fluoromax spectrofluorometers. For low-temperature measurements, a liquid nitrogen cryostat (Janis Research), equipped with optical windows, was used. All solutions were prepared immediately before measurement from the solid compound. Degassed solutions were prepared in a sealed quartz cuvette (equipped with a screw cap and silicon septum) by purging with argon for 10 min through a needle. The dispersed drop-cast films were fabricated from blended chloroform solutions of **MHTX** (0.5 mg/mL) and Zeonex® (100 mg/mL) at various ratios. A 100 µL aliquot of the mixture was deposited onto a quartz substrate, and the film was formed by solvent evaporation at 40 °C on a heating plate. Neat films were prepared by depositing 100 µL of the chloroform solution of **MHTX** (0.5 mg/mL) directly onto the quartz substrate, followed by solvent evaporation at 40 °C.

Time-resolved photoluminescence spectra were measured using pulse excitation (10 Hz) from both a Nd:YAG laser, with excitation wavelength at 355 nm, pulse length 150 ps (SL312, EKSPLA), and from a Nitrogen laser with excitation wavelength at 337 nm. Emission is captured by a sensitive gated intensified charge-coupled device (iCCD) camera from Stanford Computer Optics 4Picos gated intensified CCD camera, with a temporal resolution of 200 ps. Emission decays were constructed by varying the CCD gate delay time relative to the laser trigger and monitoring the change in the integrated spectral area (normalized by the gate time). Time delays (TDs) ranged from 1.1 ns to 39.8 ms. Decay curves were collected by time-correlated single photon counting (TCSPC) with a Horiba DeltaFlex system and Horiba DeltaDiode (405 nm) and SpectraLED (330 nm) as excitation sources. The quantum yields were determined both in thin films and solutions using a Hamamatsu Photonics Absolute Quantum Yield Measurement System model c9920–02 G with an integrating sphere, upon excitation at 310 and 355 nm.

### Theoretical calculations

DFT calculations were performed at the B3LYP/6-31 G(d,p) level for the ground state and TD-B3LYP/6-31 G(d,p) within the Tamm-Dancoff approximation for the excited states. For the dimer calculations, dispersion correction was applied using the DFT-D3 method with Becke-Johnson damping (GD3BJ). The solvent effect of 2-MeTHF was included through the polarizable continuum model (PCM) with a static dielectric constant of 6.97 and an optical dielectric constant of 1.98. The excited-state geometries of the monomer and dimer were fully optimized, and their stability was confirmed by vibrational frequency analysis. The S_1_ and S_2_ excited states of the monomer were also computed using a more recent method, PBE0/def2-TZVP with GD3BJ^45^, yielding results comparable to those obtained at the B3LYP/6-31 G(d,p) level (Fig. S32); therefore, B3LYP/6-31 G(d,p) was employed in the present study. The above calculations were performed using Gaussian 16 Rev. C02^46^. The spin-orbit coupling (SOC) constants between excited states were computed using Multiwfn^47^. The emission spectrum, accounting for all vibrational modes, was computed within the displaced harmonic oscillator approximation^48,49^. For S_1_ and S_2_ fluorescence of the monomer and S_1_ fluorescence of the dimer, the displacement vector between the initial (S_1_ or S_2_) and final (S_0_) electronic states was determined within the crude adiabatic approximation using the energy gradient of the final state at the equilibrium geometry of the initial state^50^. In contrast, for T_1_ phosphorescence of the monomer, the displacement vector was obtained within the Born-Oppenheimer approximation due to the substantial structural reorganization^51^.

## Supplementary information

Supplementary Information

Transparent Peer Review file

## Source data

Source Data 1

Source Data 2

## Data Availability

The data generated in this study are provided in the Data Source file. Data required to support the findings of this study are available from the corresponding author(s) upon request. Source data are provided with this paper.
