# Supplementary material for: Anti-Kasha fluorescence and persistent room-temperature phosphorescence from a nearly flat non-symmetric polycyclic hydrocarbon
Source: Nat Commun. 2026 Oct 2;17:11300. doi: 10.1038/s41467-026-78107-6 (PMC13724719; doi:10.1038/s41467-026-78107-6)
Supplement: Supplementary file 1 [file 41467_2026_78107_MOESM1_ESM.pdf]

Supplementary Information

Anti-Kasha fluorescence and persistent room-temperature phosphorescence from a nearly flat non-symmetric polycyclic hydrocarbon

Monike da Silva Kutz[1,2#], Carolina Francener[3#], Wataru Ota[4,5], Manuela Santos Corrêa[1], Daniel Crane[3], Emiko Fujiwara[4], Tohru Sato[4,5*], Ivan H. Bechtold[6], Andrew P. Monkman[3*], Eduard Westphal[1*], Harald Bock[2]

[1] Department of Chemistry, Universidade Federal de Santa Catarina, Florianópolis, Brazil
[2] Centre de Recherche Paul Pascal, Université de Bordeaux/Centre National de la Recherche Scientifique, Pessac, France
[3] Department of Physics, Durham University, Durham, United Kingdom
[4] Fukui Institute for Fundamental Chemistry, Kyoto University, Takano Nishibirakicho 34-4, Sakyo-ku, Kyoto 606-8103, Japan
[5] Department of Molecular Engineering, Graduate School of Engineering, Kyoto University, Nishikyo-ku, Kyoto 615-8510, Japan
[6] Department of Physics, Universidade Federal de Santa Catarina, Florianópolis, Brazil

[#]These authors contributed equally: Monike da Silva Kutz, Carolina Francener

[*]Correspondence should be addressed to T.S. (email: tsato@scl.kyoto-u.ac.jp), A.P.M. (email: a.p.monkman@durham.ac.uk) or to E.W. (email: eduard.w@ufsc.br)

Content

1. **Synthesis and Structural Characterization** 2
   1.1. *Synthesis of 2-(1-indanylidene)-1-indanone (**2**)* 2
   1.2. *Synthesis of monohomotruxene (**MHTX**)* 2
   1.3. *Optimization of reaction conditions for the synthesis of **MHTX*** 3
   1.4. *Synthesis of homotruxene (**HTX**)* 3
   1.5. *Synthesis of truxene (**TX**)* 4
2. **NMR Spectra** 5
3. **High Resolution Mass Spectrometry** 9
4. **Photophysical Investigations** 10
   4.1. *Singlet quenching* 11
   4.2. *Antisolvent experiment* 14
   4.3. *Room-temperature time-resolved photoluminescence in solid-state films* 18
   4.4. *Low-temperature time-resolved photoluminescence in dilute solution* 19
   4.5. *Time-resolved photoluminescence of MHTX as a neat film* 20
5. **DFT Calculations** 23
6. **References** 29

1. Synthesis and Structural Characterization

Commercial reagents and solvents were used as supplied (Merck, Sigma-Aldrich, Fluka and Acros Organics). Dichloromethane (DCM) was dried using an Innovative Technology PS-MD-5 Solvent Purification System (SPS). Reactions were monitored by thin-layer chromatography (TLC) on aluminum plates coated with silica gel and UV254 nm indicator. Purifications were performed by column chromatography on silica gel (60–200mesh, 60Å); by Gel Permeation Chromatography (GPC), using a JAI FC-3480 Recycling Preparative HPLC a JAIGEL-2.5HR-40 column, with HPLC-isocratic grade chloroform stabilized with ethanol as eluent; and recrystallizations using commercial-grade solvents. Melting points were measured in a polarized optical microscope Olympus BX53 coupled to a Mettler Toledo FP-82 hot stage. ^{1}H and ^{13}C NMR spectra were recorded with a JEOL ECS-400 spectrometer operating at 400 MHz and 100.6 MHz. Infrared (IR) spectra were obtained in a Bruker spectrometer, model Alpha, using KBr pellets. Elemental analysis was performed using a Perkin-Elmer series II CHNS/O Analyzer (model 2400) coupled to a Perkin-Elmer Autobalance AD 6000. Mass spectrometry analyses were performed using a Bruker Autoflex Speed matrix-assisted laser desorption/ionization time-of-flight (MALDI-TOF) mass spectrometer (Bruker Daltonics), equipped with a 2 kHz Smartbeam-II laser system. The instrument was operated in the reflectron mode, detecting positive ions.

*1.1. Synthesis of 2-(1-indanylidene)-1-indanone (**2**)*

Under argon atmosphere and strong stirring, a solution of 1-indanone (**1**, 3.3 g, 25 mmol) in anhydrous dichloromethane (DCM) (25 mL) was rapidly added at 0 °C via an addition funnel to a solution of $TiCl_4$ (0.13 mL, 1.2 mmol) in hexane (5 mL) . Subsequently, a second equivalent of 1-indanone (**1**, 3.3 g, 25 mmol), along with triethylamine (3.8 mL, 27.5 mmol) in anhydrous DCM (25 mL), was added dropwise over a period of 20 minutes. After complete addition, the dark-green mixture was stirred at 0 °C for 30 minutes and then at 298 K for 16 hours[1]. Afterward, the mixture was slowly poured into acetone (75 mL), the precipitate was filtered and washed with hot ethanol (100 mL). The light-yellow remaining solid was purified by recrystallization from toluene (150 mL).

Yield: 2.46 g (246.3 g mol^{-1}, 10 mmol, 40%) of a light-yellow solid. **R_f** = 0.5 (50% DCM:petroleum ether (PE)); **m.p.**: 144 – 146 ºC (lit. 143 – 144 °C)[2,3]; **^{1}H NMR** ($CDCl_3$, 400 MHz, 22 °C) δ ppm: 7.86 (d, J = 7.8 Hz, 1H, Ar-H), 7.83 (d, J = 7.8 Hz, 1H, Ar-H), 7.60 – 7.52 (m, 2H, Ar-H), 7.46 – 7.32 (m, 4H, Ar-H), 4.05 (s, 2H, $-CH_2-$), 3.61 – 3.55 (m, 2H, $-CH_2-$), 3.18 – 3.13 (m, 2H, $-CH_2-$). **^{13}C NMR** ($CDCl_3$, 100.6 MHz, 22 °C) δ ppm: 195.4, 155.2, 151.9, 148.7, 141.0, 139.7, 133.7, 130.6, 127.4, 127.0, 126.4, 126.1, 126.0, 125.9, 123.7, 33.1, 31.7, 31.0.

*1.2. Synthesis of monohomotruxene (**MHTX**)*

Under argon atmosphere, a mixture of the 1-indanone dimer (**2**, 1.0 g, 4.1 mmol), commercial 1-tetralone (**3**, 2.4 g, 16.4 mmol), propionic acid (0.53 g, 7.2 mmol) and dried *para*-toluenesulfonic acid (1.4 g, 7.1 mmol) was stirred at 140 °C for 24 hours in *ortho*-dichlorobenzene (5 mL). The reaction flask was kept wrapped in aluminum foil to protect it from light. After cooling down to room temperature, cold methanol (50 mL) was added to the flask and the acid was neutralized with excess 10% aqueous NaOH solution. The resulting precipitate was collected by filtration and washed with additional methanol (20 mL). The light-yellow remaining solid was initially purified by silica gel column chromatography (eluent: 10% DCM in petroleum ether). A second purification by gel permeation chromatography (GPC), using chloroform as the eluent (HPLC isocratic standard, stabilized with ethanol), was necessary to separate **MHTX** from impurities of similar

polarity and solubility[4]. The GPC-purified chloroform solution was concentrated to 10 mL and the product was precipitated by adding methanol (50 mL). The solid was collected by filtration, washed with methanol (10 mL) and recrystallized from acetone.

Yield: 0.57 g (356.2 g mol^{-1}, 1.6 mmol, 39%) of a white solid. R_f = 0.5 (10% DCM in PE); **m.p.**: >300 °C; **^{1}H NMR** ($CDCl_3$, 400 MHz, 22 °C) δ ppm: 8.05 (d, J = 7.7 Hz, 1H, Ar-H), 8.04 (d, J = 7.7 Hz, 1H, Ar-H), 7.95 (d, J = 7.7 Hz, 1H, Ar-H), 7.70 (d, J = 7.3 Hz, 1H, Ar-H), 7.63 (d, J = 7.3 Hz, 1H, Ar-H), 7.49 – 7.27 (m, 7H, Ar-H), 4.37 (s, 2H, $-CH_2-$), 4.30 (s, 2H, $-CH_2-$), 3.49 – 3.41 (m, 2H, $-CH_2-$), 2.93 – 2.85 (m, 2H, $-CH_2-$). **^{13}C NMR** ($CDCl_3$, 100.6 MHz, 22 °C) δ ppm: 144.1, 143.8, 142.2, 141.6, 138.85, 138.82, 138.4, 136.9, 136.4, 135.2, 133.1, 131.2, 127.8, 127.0, 126.9, 126.8, 126.6, 126.6, 126.4, 126.2, 125.3, 124.7, 123.5, 121.8, 39.5, 36.6, 29.5, 26.2. **Elemental analysis** calcd (%) for $C_{28}H_{20}$: C 94.34%; H 5.66%; found: C 93.98%; H 5.55%. **HRMS** (MALDI) m/z: $[M]^{+\cdot}$ calcd for $C_{28}H_{20}$: 356.1560; found: 356.1587.

*1.3. Optimization of reaction conditions for the synthesis of **MHTX**.*

The reaction proceeded best in *ortho*-dichlorobenzene (ODCB) as a solvent for 24 h at 140 °C, due to its high polarity, polarizability, and boiling point. Extended reaction times (48 h) did not contribute significantly to increasing the reaction yield, whereas higher temperatures (180 °C) promoted a reduction in yield, possibly due to the partial evaporation of propionic acid (boiling point = 141.2 °C), which is proposed to be the direct catalyst after protonation by PTSA[5]. Furthermore, higher catalyst concentrations proved detrimental, likely by promoting over-protonation and excessive enol formation of the ketone substrates.

Table S1. Optimization of reaction conditions for the synthesis of **MHTX**.

**Entry**	**Catalysts** / equiv.[a]	**1-tetralone** / equiv.[b]	**Solvent**[c]	**Temperature** / °C	**Time** / h	**Yield** / %[d]
**1**	1	4	ODCB	140	24	10
**2**	1.75	4	ODCB	140	24	39
**3**	3	4	ODCB	140	24	5
**4**	1.75	2	ODCB	140	24	15
**5**	1.75	4	Toluene	105	24	traces
**6**	1.75	4	ODCB	140	48	40
**7**	1.75	4	ODCB	180	24	26

[a] Molar equivalents of PTSA and propionic acid, considering 1 equivalent of the dimer.
[b] Molar equivalents of 1-tetralone, considering 1 equivalent of the dimer.
[c] The concentration of the reactions was maintained at 0.7 mol/L related to the dimer.
[d] Yield of the pure product.

*1.4. Synthesis of homotruxene (**HTX**).*

Under anhydrous conditions and argon atmosphere, a solution of 1-tetralone (**3**) (17.6 g, 0.12 mmol) and DABCO (13.6 g, 0.12 mol) in DCM (80 mL, anhydrous) was rapidly added via an addition funnel to a solution of $TiCl_4$ (32 mL, 0.30 mol) in DCM (80 mL, anhydrous). The reaction is exothermic, and immediately turns dark purple, releasing vapors that were collected in a bubbler glassware filled with ODCB. Subsequently, 80 mL of anhydrous DCM was added. The mixture was then sonicated for 2 minutes to break bigger aggregates, and vigorously stirred at room temperature for 3 hours. Afterward, the flask content was slowly poured into ice/water (300 mL), the product was extracted with DCM (2 x 500 mL) and the organic phase was dried over $MgSO_4$. After removal of solvent under vacuum, the remaining dark oil was purified by chromatography column of silica gel (100/400 μm) with a mixture of 10% of DCM in PE as eluent. The yellowish remaining

solid was solubilized in DCM (6 mL) and the product was precipitated by the addition of cold ethanol (150 mL). The product was collected by filtration and washed with methanol (20 mL).

Yield: 3.21 g (384.19 g mol^{-1}, 8.3 mmol, 21%) of a white solid. $\boldsymbol{R_f}$ = 0.46 (25% DCM in hexane). **m.p.**: > 300 °C. **^{1}H NMR** ($CDCl_3$, 400 MHz, 22 °C) δ ppm: 7.46 (dd, J = 2 Hz, J = 8 Hz, 3H), 7.32 (dd, J = 2 Hz, J = 8 Hz, 3H), 7.28 (dt, J = 2 Hz, J = 8 Hz, 3H), 7.26 (dt, J = 2 Hz, J = 8 Hz, 3H), 3.35 (broad, 3H), 2.94 (broad, 3H), 2.73 (broad, 3H), 2.63 (broad, 3H). **^{13}C NMR** ($CDCl_3$, 100.6 MHz, 22 °C) δ ppm: 140.2, 136.1, 135.1, 133.3, 129.1, 126.9, 126.8, 125.7, 30.6, 30.5 [6].

*1.5. Synthesis of truxene (**TX**)*

A mixture of 1-indanone (**1**, 0.4 g, 3 mmol), propionic acid (0.7 g, 9 mmol) and dried *para*-toluenesulfonic acid (1.5 g, 9 mmol) was stirred at 140 °C for 24 hours in *ortho*-dichlorobenzene (5 mL) under argon atmosphere. The reaction flask was kept wrapped in aluminum foil to protect it from light exposure. After cooling down to room temperature, cold methanol (50 mL) was added to the flask and the acid was neutralized with 10% aqueous NaOH solution. The precipitate was collected by filtration and washed with additional methanol (20 mL). The light-yellow remaining solid was initially purified by silica gel column chromatography (eluent: 10% DCM in petroleum ether) and then boiled in toluene, followed by hot filtration to separate the product from the mother liquor.

Yield: 0.2 g (342.4 g mol^{-1}, 0.6 mmol, 58%) of a light-yellow solid. $\boldsymbol{R_f}$ = 0.53 (25% DCM in hexane) **m.p.**: >300 °C. **^{1}H NMR** ($CDCl_3$, 400 MHz, 22 °C) δ ppm: 7.91 (d, J = 7.4 Hz, 3H, Ar-H), 7.67 (d, J = 7.4 Hz, 3H, Ar-H), 7.48 (t, J = 7.4 Hz, 3H, Ar-H), 7.38 (td, J= 7.4 Hz, J = 0.8 Hz, 3H, Ar-H), 4.20 (s, 6H, -CH_2-). **^{13}C NMR** ($CDCl_3$, 100.6 MHz, 22 °C) δ ppm: 143.8, 141.7, 137.1, 135.3, 126.9, 126.3, 125.1, 121.9, 36.5 [7].

2. NMR Spectra

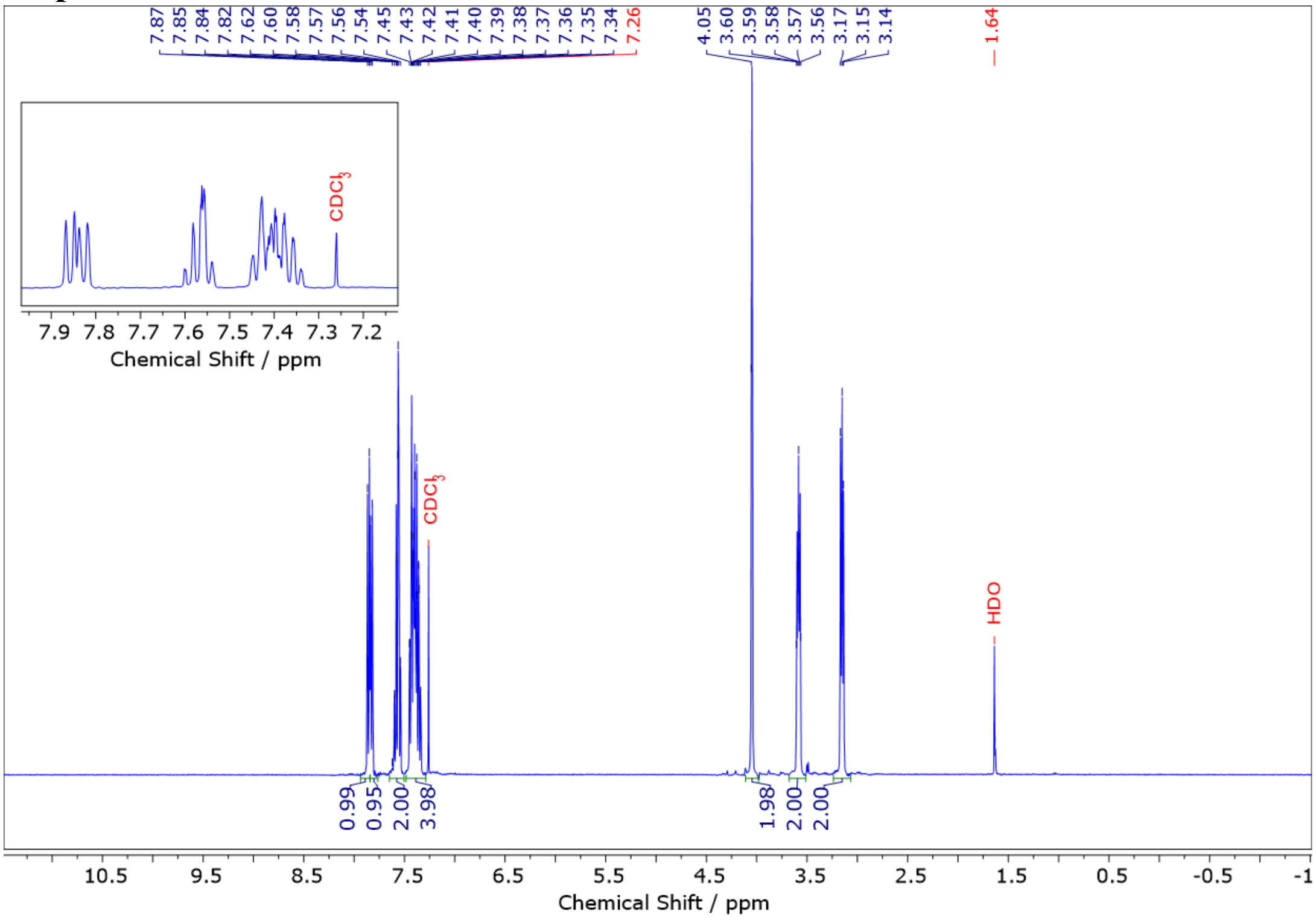

Figure S1. ^{1}H NMR spectrum of dimer (**2**) in $CDCl_3$ (400 MHz). The inset is the magnification of the aromatic region.

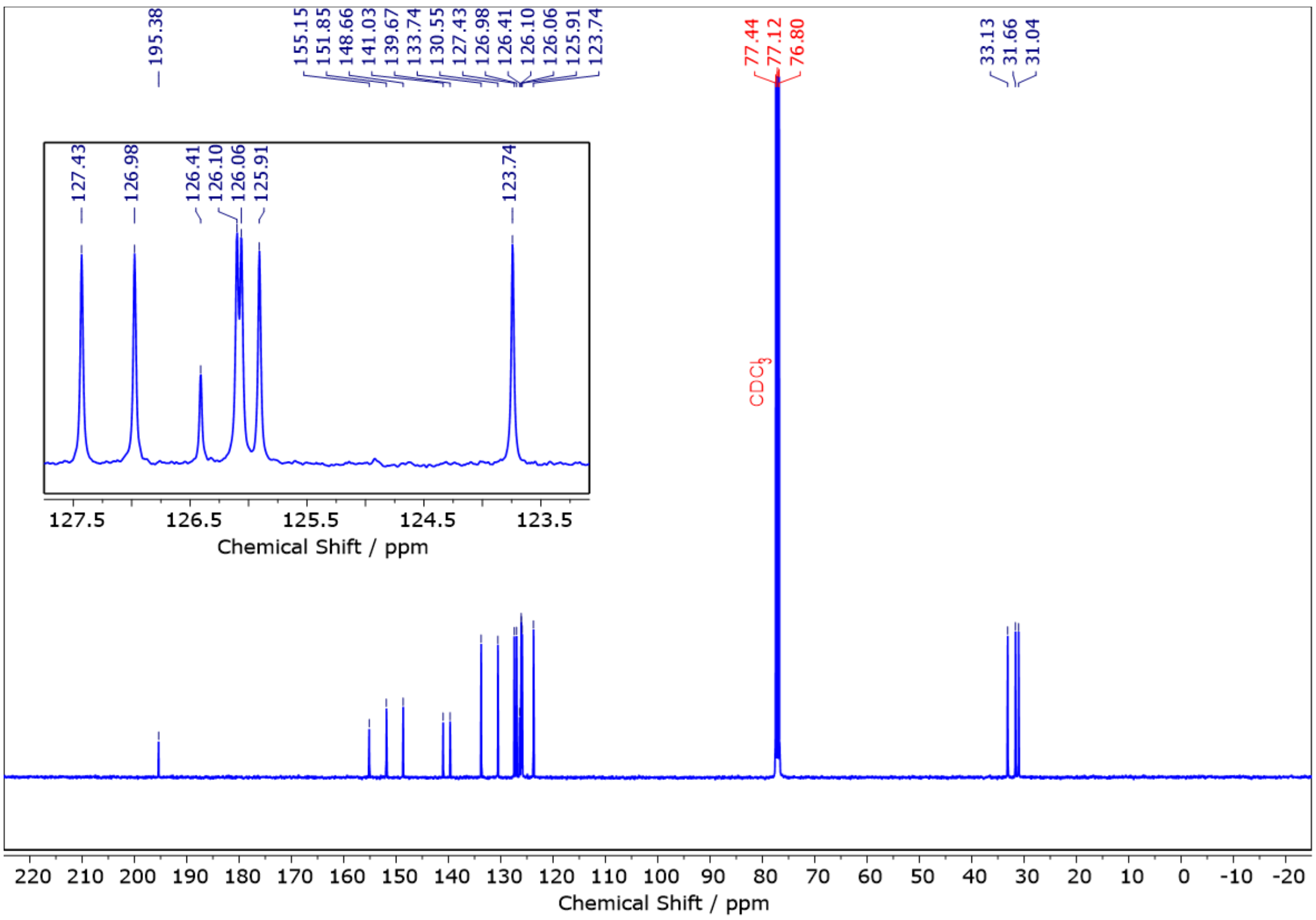

Figure S2. ^{13}C NMR spectrum of dimer (**2**) in $CDCl_3$ (100.6 MHz). The inset is the magnification of part of the aromatic region.

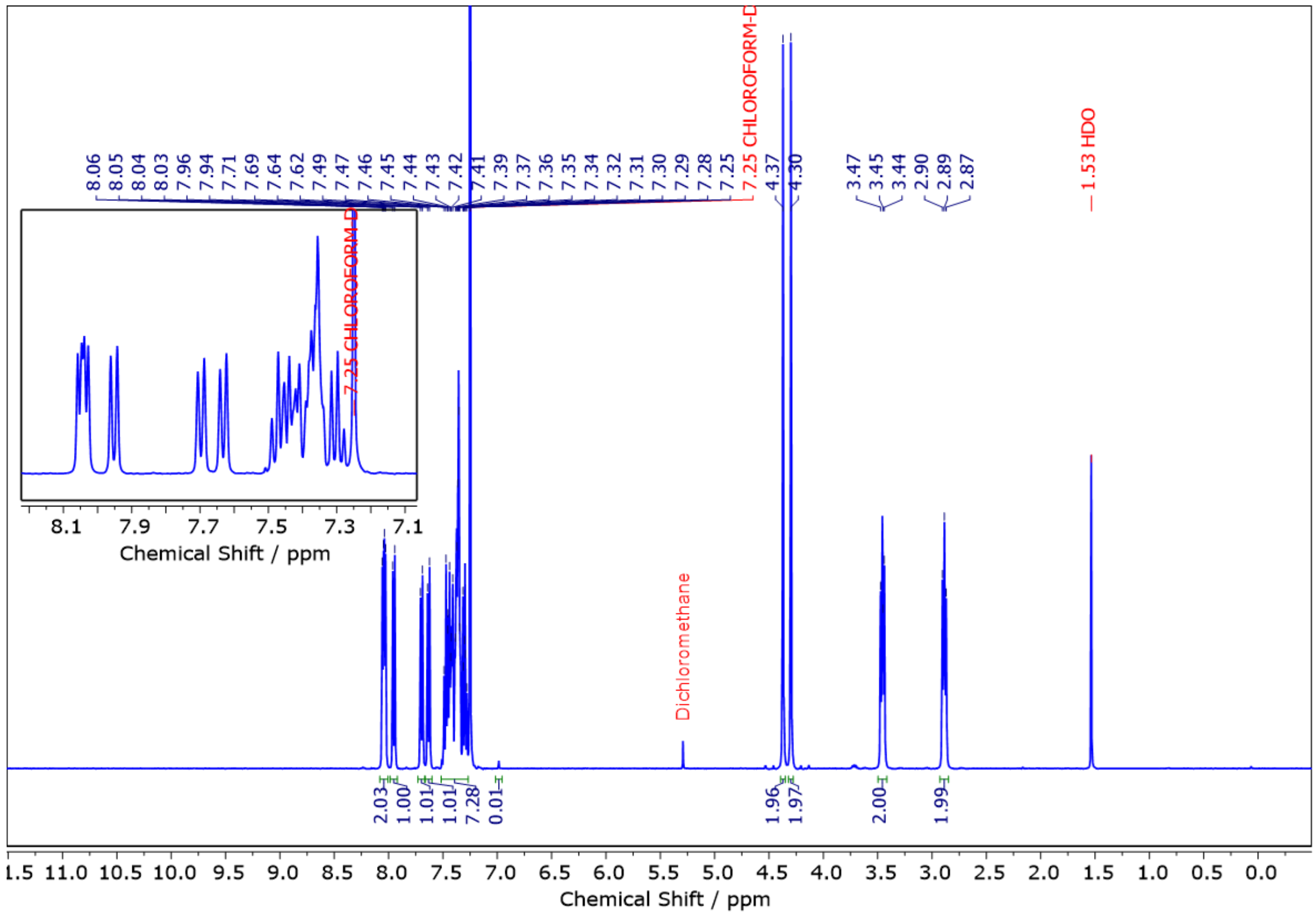

Figure S3. ^{1}H NMR spectrum of **MHTX** in $CDCl_3$ (400 MHz). The inset is the magnification of the aromatic region.

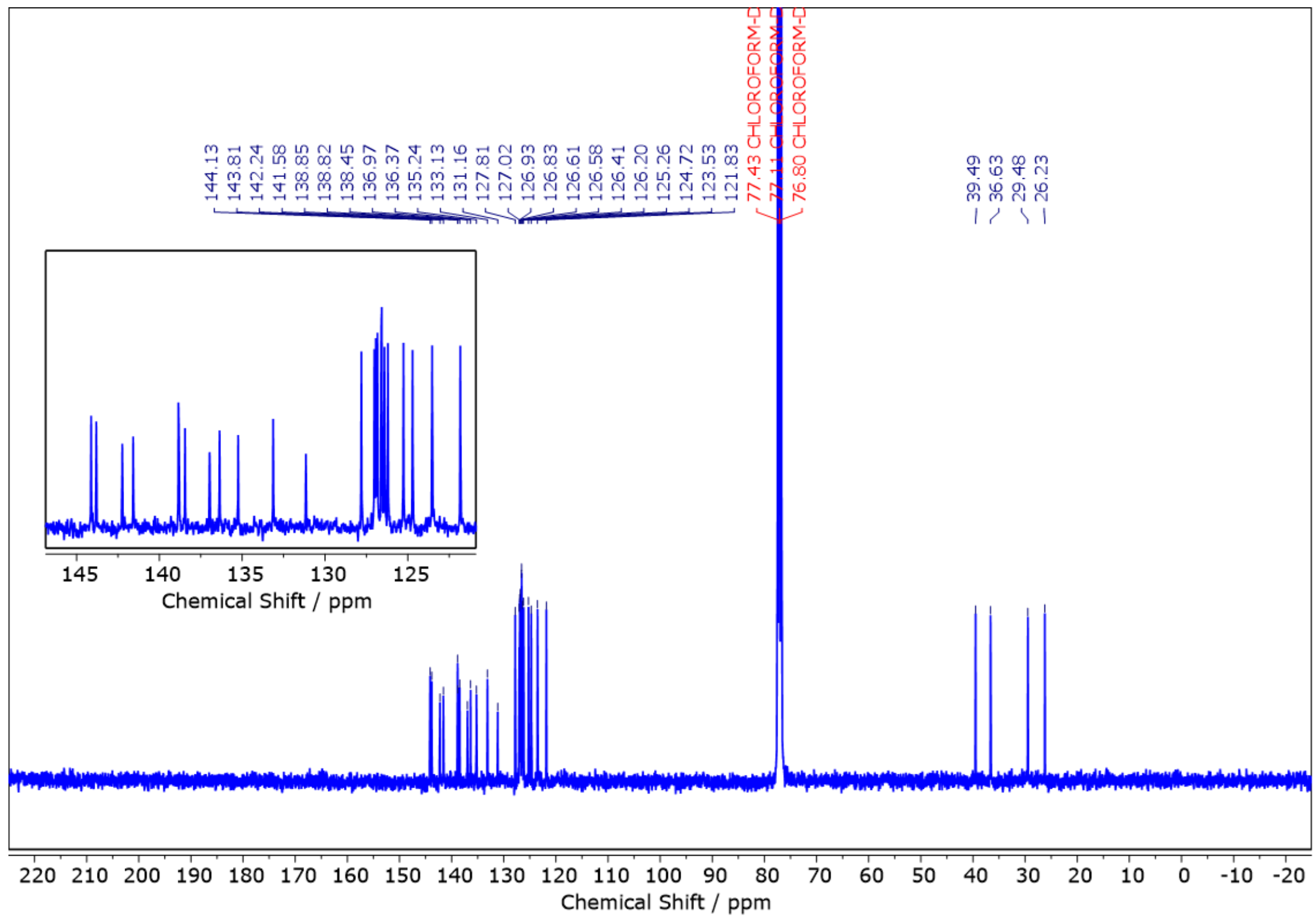

Figure S4. ^{13}C NMR spectrum of **MHTX** in $CDCl_3$ (100.6 MHz). The inset is the magnification of the aromatic region.

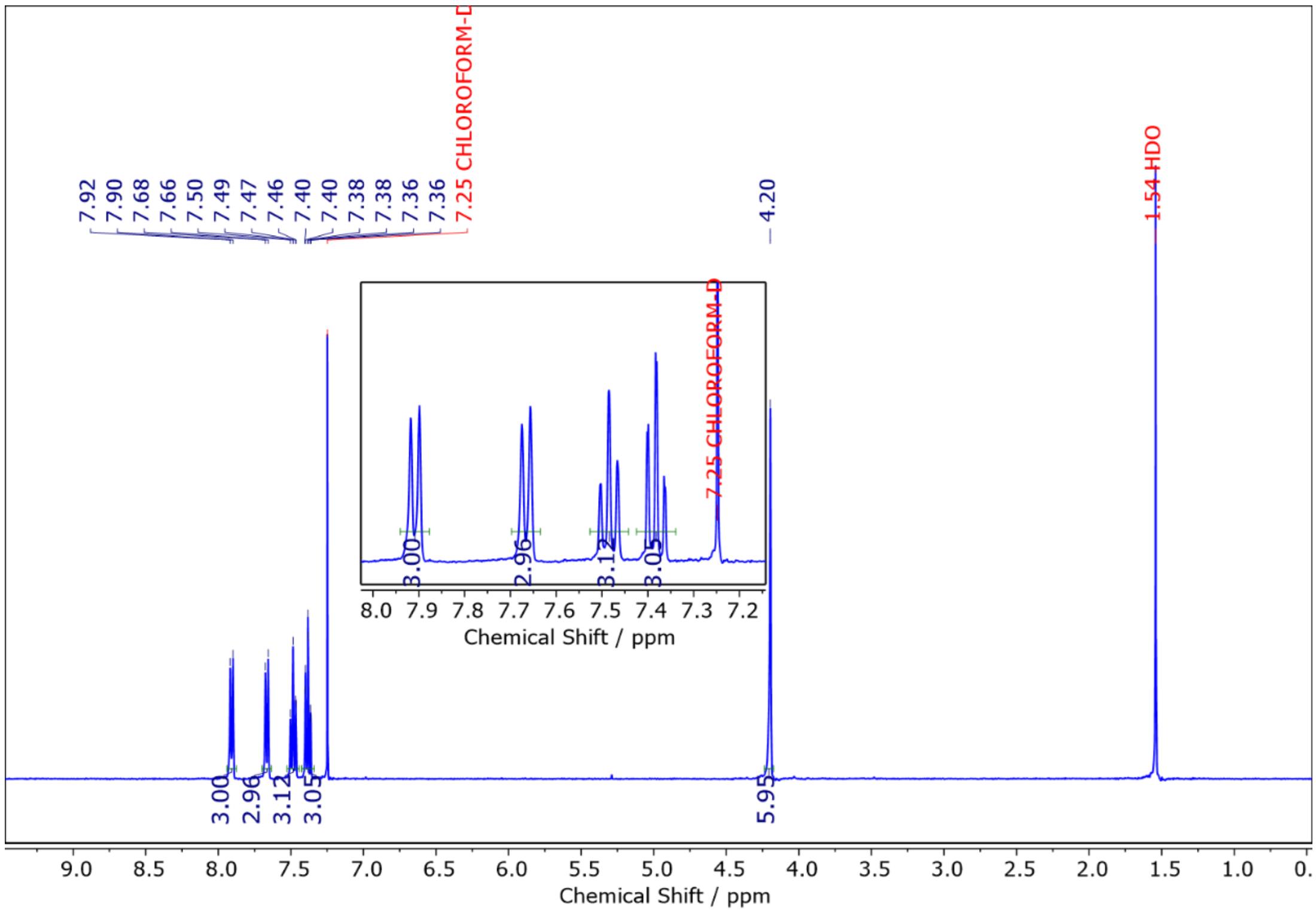

Figure S5. ^{1}H NMR spectrum of **Truxene** (**TX**) in $CDCl_3$ (400 MHz). The inset is the magnification of the aromatic region.

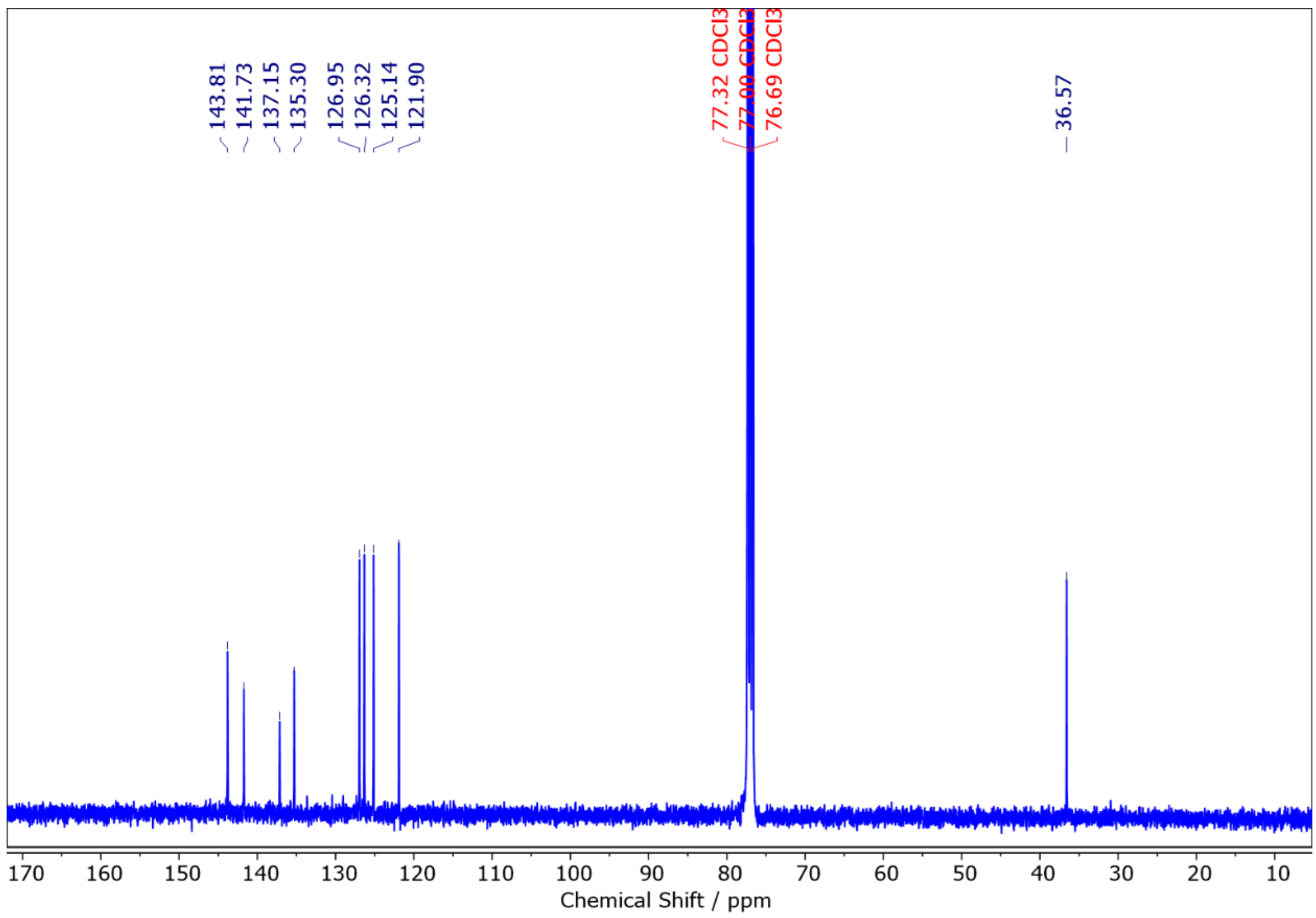

Figure S6. ^{13}C NMR spectrum of **Truxene** (**TX**) in $CDCl_3$ (100.6 MHz).

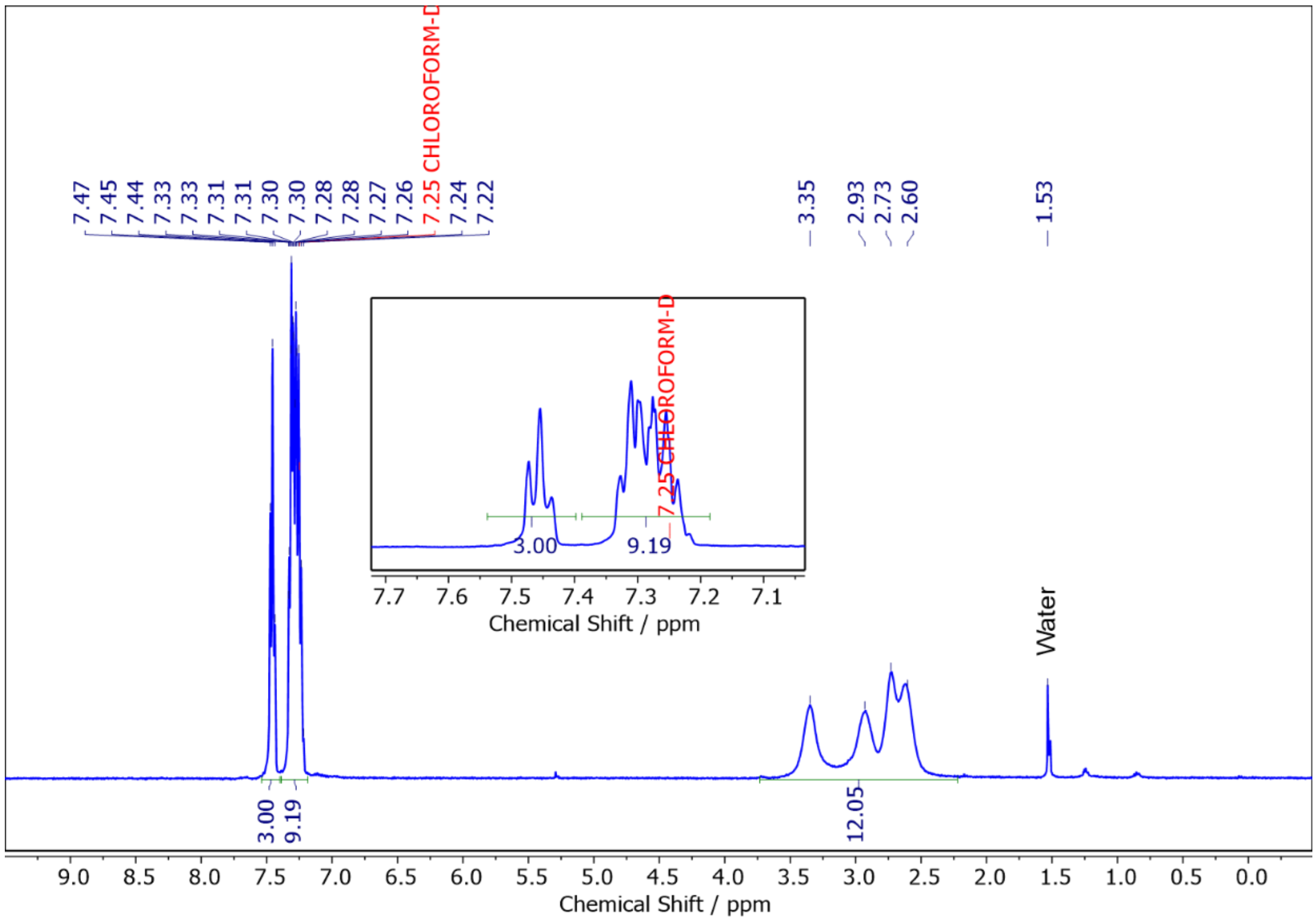

Figure S7. ^{1}H NMR spectrum of **Homotruxene** (**HTX**) in $CDCl_3$ (400 MHz). The inset is the magnification of the aromatic region.

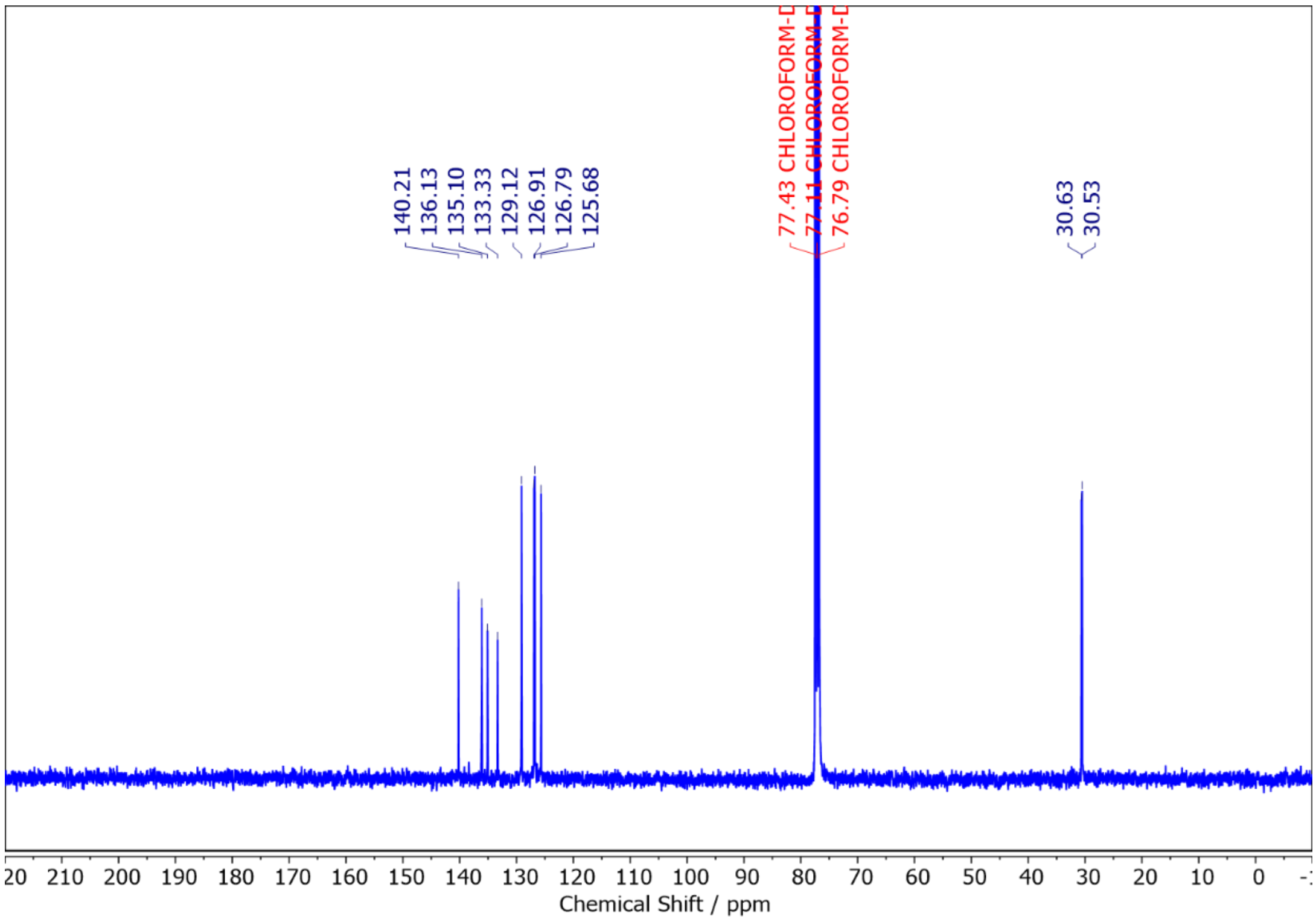

Figure S8. ^{13}C NMR spectrum of **Homotruxene** (**HTX**) in $CDCl_3$ (100.6 MHz).

3. High Resolution Mass Spectrometry

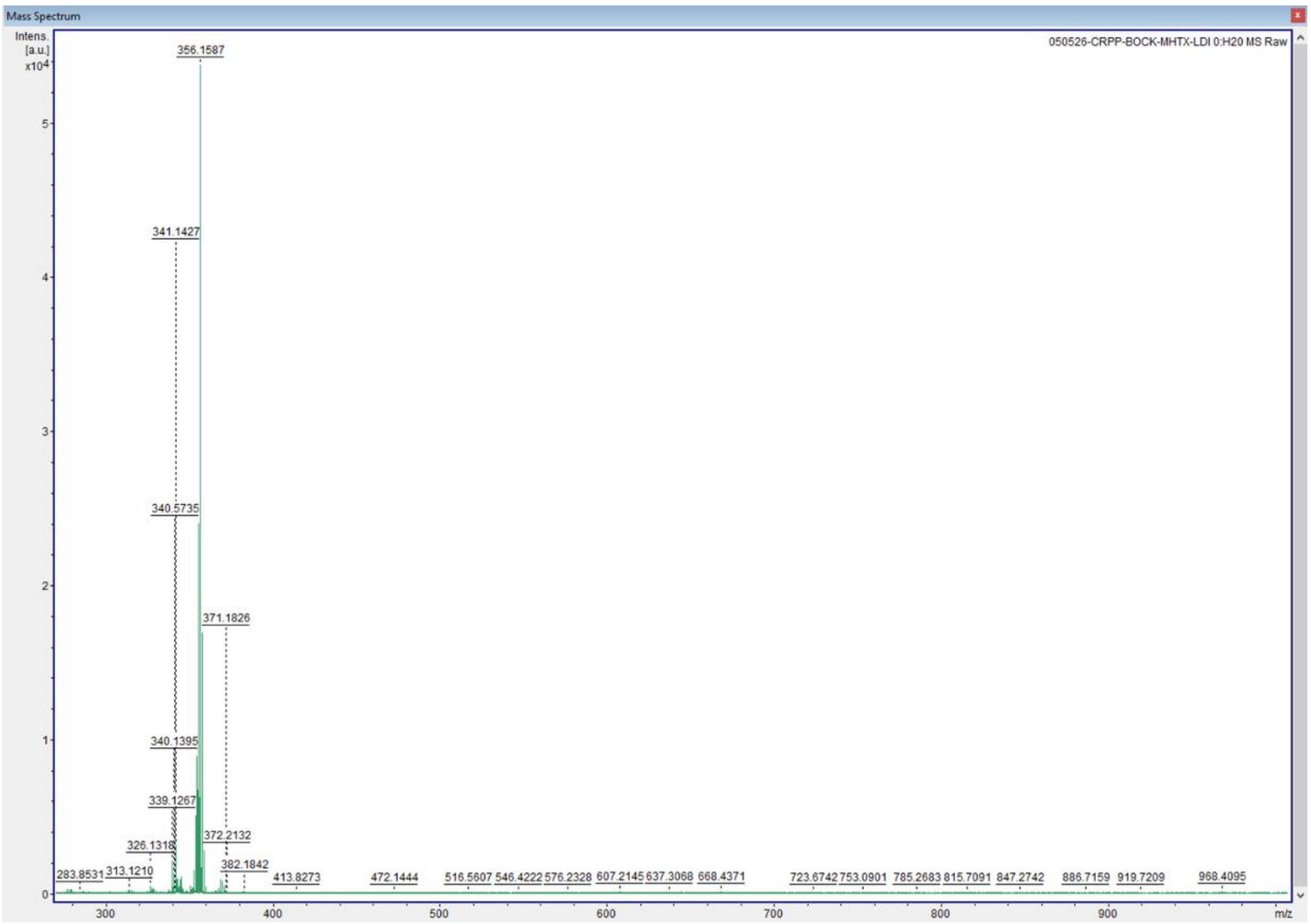

Figure S9. High Resolution Mass Spectrometry (HRMS) spectrum. HRMS spectrum of **MHTX** recorded on MALDI-type mass spectrometer equipped with a ToF analyzer via laser desorption ionization (LDI) in positive ion mode. The principal peak observed at m/z = 356.1587 corresponds to the expected molecular ion (M^+).

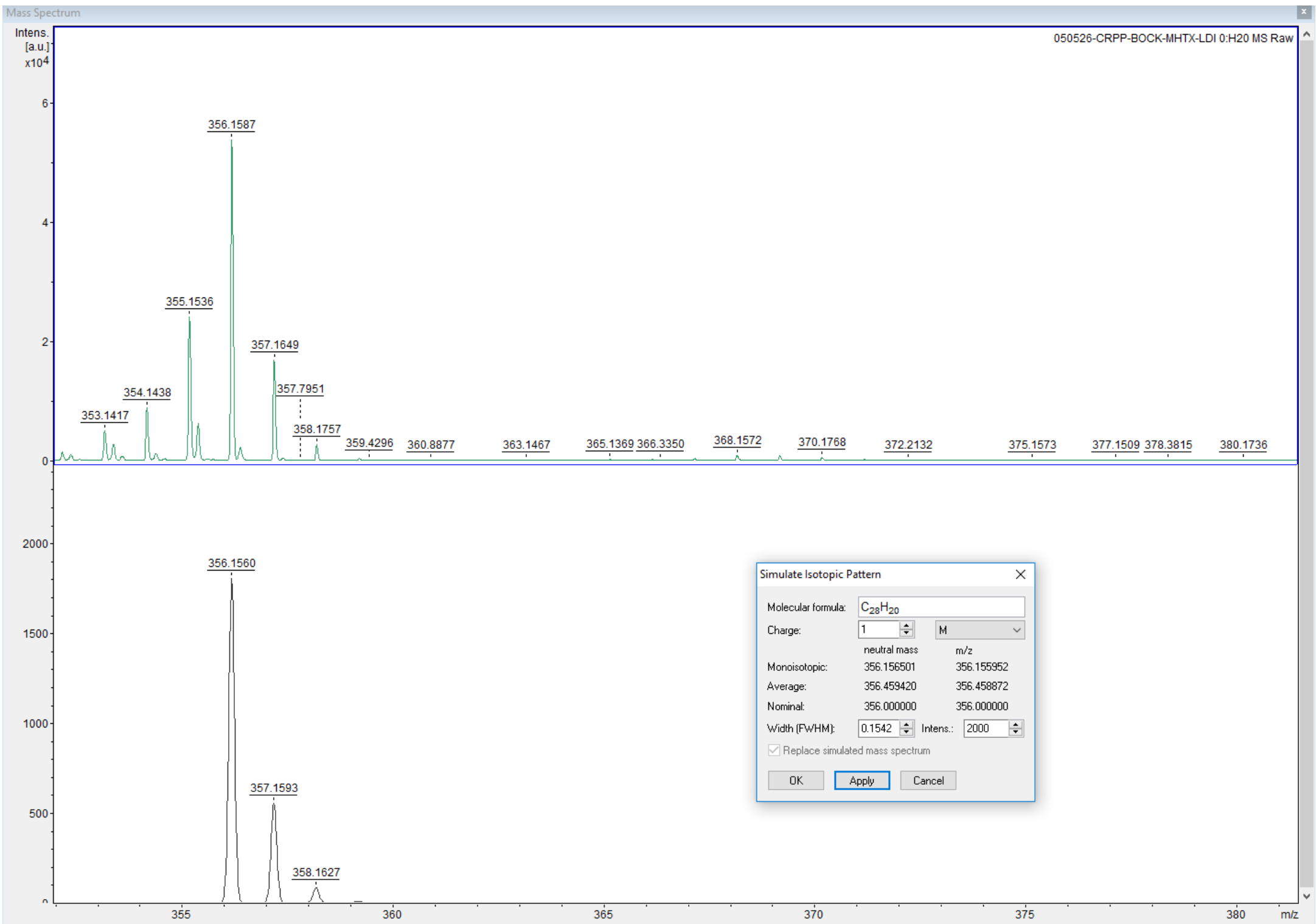

Figure S10. High-resolution mass spectrometry (HRMS) isotopic pattern confirmation. Comparison between the experimental (top) and simulated (bottom) HRMS isotopic patterns for **MHTX**. The simulated pattern was generated based on the molecular formula $[C_{28}H_{20}]^+$ (monoisotopic m/z = 356.1560).

4. Photophysical Investigations

Anti-Kasha fluorescence of MHTX

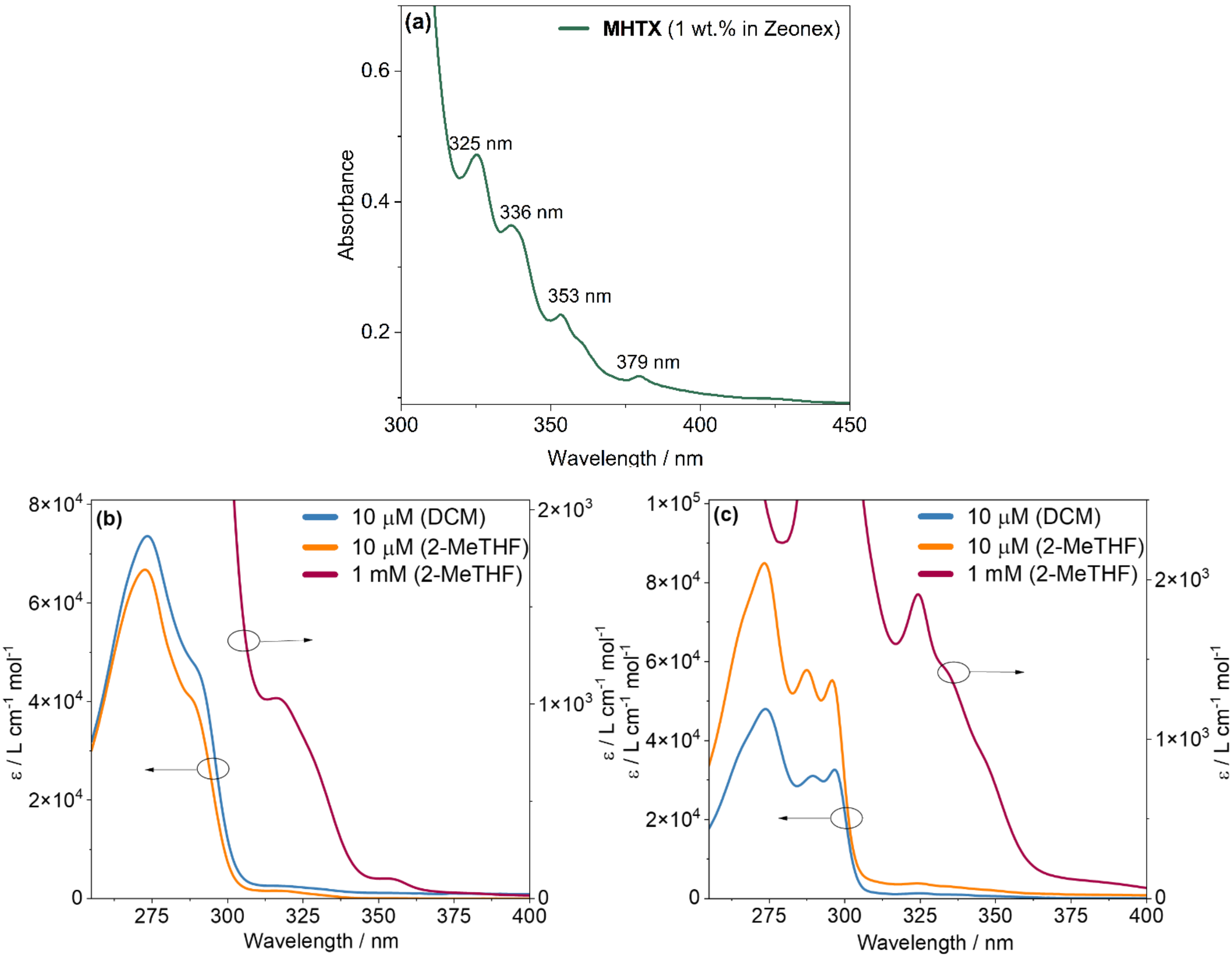

Figure S11. Steady-state absorption spectra. (a) Absorption spectrum of **MHTX** in the solid state (1 wt% in Zeonex). Absorption spectra of **(b) HTX** and **(c) TX** recorded as 10 μM solutions in 2-MeTHF (orange) and DCM (blue), alongside a 1 mM solution in 2-MeTHF (dark red).

Table S2. Steady-state photophysical properties of **MHTX** in solution and solid-state environments.

Conditions	λ_{AB} / nm ($\varepsilon \times 10^4$ / L cm^{-1} mol^{-1})	λ_{Em} (S_1)[a] / nm	λ_{Em} (S_2)[b] / nm	Stokes shift ($\Delta\nu$)[c] / eV	PLQY[d]
Solution	271 (7.6)<br>288 (4.9)<br>297 (4.5)<br>325 (0.14)<br>336 (0.1)<br>353 (0.04)<br>379 (0.006)	350, 360, 380, 400, 425	384, 404, 425	0.15 (S_2)<br>0.05 (S_1)	57% (310 nm) and 32% (355 nm)
1 wt.% Zeonex film	325, 336, 353. 379	350, 360	384, 404, 425	0.15 (S_2)<br>0.05 (S_1)	49% (310 nm) and 63% (355 nm)

[a] and [b] Excited at 300 nm and 355 nm, respectively. Data related to the absorption spectrum of a dilute solution in 2-methyltetrahydrofuran, 2-MeTHF (10 μM). [c] Estimated from the wavelengths of the longest λ_{AB} and the shortest λ_{PF} from each decay state, as indicated in parentheses. [d] Absolute quantum yield determined by using an integrating sphere system, upon excitation at 310 nm and 355 nm (degassed 20 μM in 2-MeTHF).

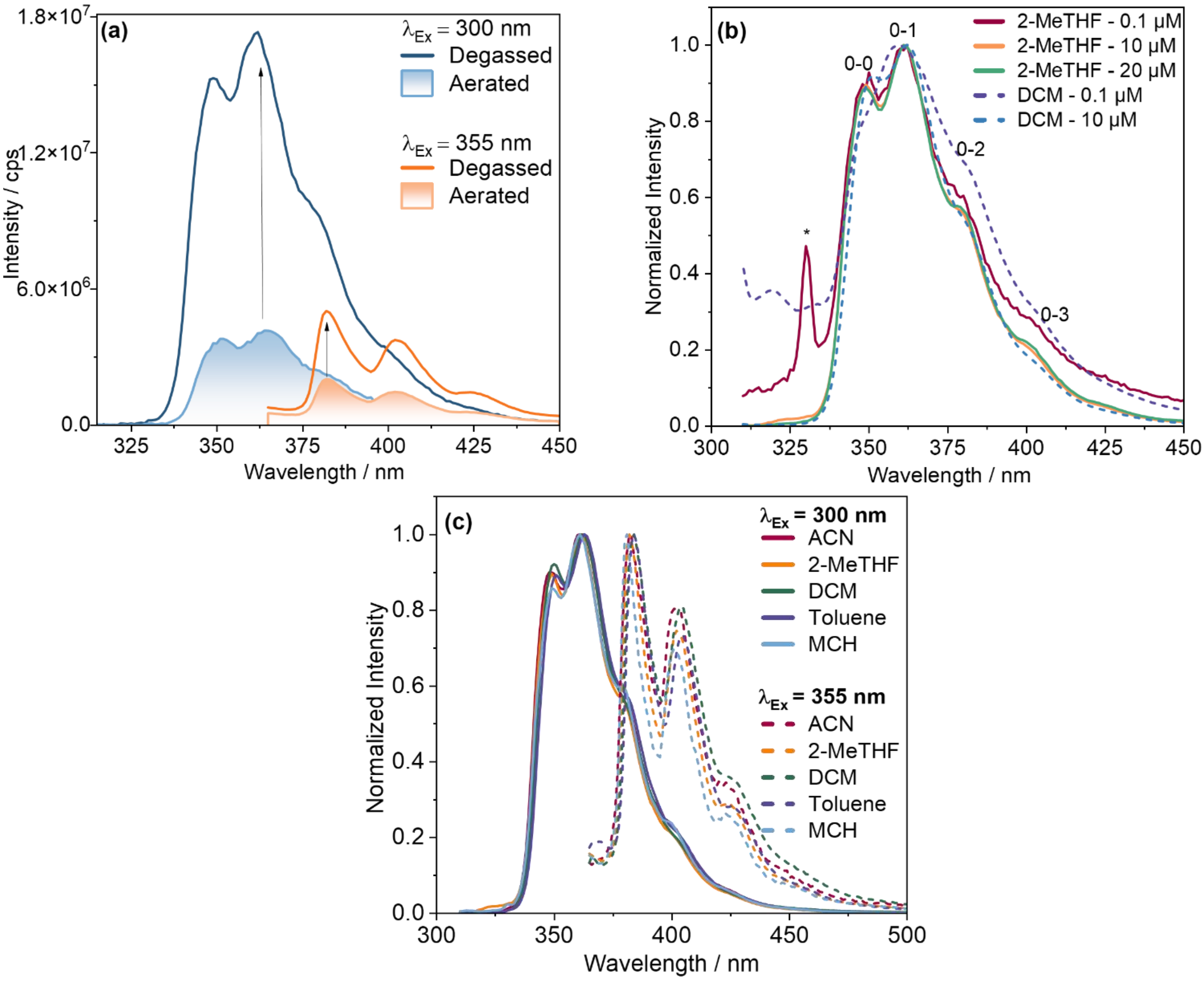

Figure S12. Photophysical properties of MHTX as a function of environment and concentration. (a) **Monomeric dual emission and sensitivity to molecular oxygen**. Steady-state emission spectra of **MHTX** (10 µM in 2-methyltetrahydrofuran, 2-MeTHF) at 298 K under aerated (shaded) and degassed (solid lines) conditions. Selective excitation at λ_{Ex} = 300 nm (blue) and 355 nm (orange) reveals intensity enhancement (indicated by arrows) upon deaeration for both S_2 and S_1 manifolds. (b) **Concentration- and solvent-dependent emission profiles**. Normalized emission spectra in 2-MeTHF (solid lines) and dichloromethane, DCM (dashed lines) across a range of concentrations. Vibronic progressions (0 → ν') are labeled for the monomeric bands. The emergence of lower-energy features at high concentrations indicates the onset of intermolecular coupling. Asterisk (*) denote solvent Raman scattering. **(c) Solvent-dependent spectral shifts (solvatochromism).** Normalized emission spectra of **MHTX** (10 µM) in solvents of varying polarity upon excitation at λ_{Ex} = 300 nm (solid lines) and 355 nm (dashed lines). MCH, methylcyclohexane; ACN, acetonitrile.

4.1. Singlet quenching (Figures S13 – S14)

To elucidate the excited-state kinetics of **MHTX**, time-resolved photoluminescence (TRPL) measurements were performed on diluted solutions (10 µM in 2-MeTHF) under both aerobic and anaerobic conditions (Fig. S13). The obtained lifetimes are summarized in Table S3. Time-gated measurements at room temperature for aerated and degassed solutions reveal a broad prompt fluorescence (PF) band, with maxima at 390 and 405 nm upon excitation at 337 and 355 nm, respectively (Figure S13a–d). The observed red shift in the S_2 emission maxima compared to steady-state measurements is attributed to an internal filter effect, which attenuates the high-energy UV region of the emission band. In the lower-energy spectral region (500–600 nm), a broad and

weak emission feature is observed; we attribute this to the radiative decay from the S_1 state of higher-order aggregated species.

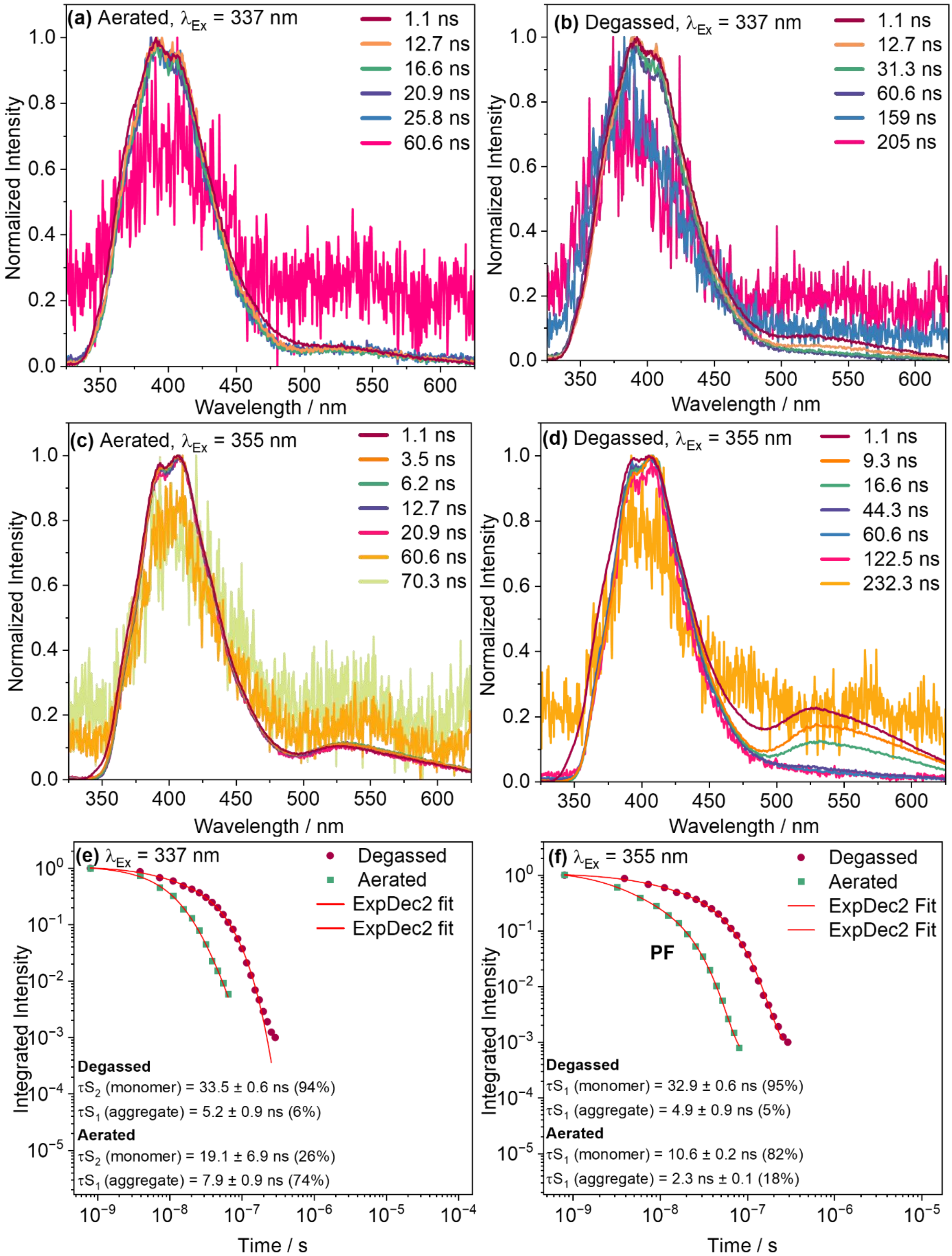

Figure S13. Time-resolved photoluminescence of MHTX in solution. (a–d) Time-gated emission spectra of **MHTX** in 2-MeTHF (10 μM) recorded under various atmosphere and excitation conditions: (a and c) Aerated solutions excited at 337 nm and 355 nm, respectively; (b and d) degassed solutions containing dissolved molecular oxygen. **(e–f)** Photoluminescence decay curves obtained from time-gated measurements in 2-MeTHF (10 μM) under degassed (red circles) and aerated (green squares) conditions, recorded upon excitation at (e) 337 nm (S_2 state) and (f) 355 nm (S_1 state).

The prompt fluorescence decay of the S_2 state of monomeric species in degassed solution exhibits a long lifetime (τ_{PF}) of 29.7 ns (λ_{Ex} = 337 nm; Figure S13e). Similarly, the monomeric S_1 decay (λ_{Ex} = 355 nm; Figure S13f) possesses a τ_{PF} of 32.9 ns. Biexponential fitting of the 355 nm excitation data reveals a shorter-lived component attributed to aggregate species, which contributes 5% to the total emission in aerated media and increases to 18% upon deoxygenation. In aerated solutions, the presence of dissolved oxygen effectively quenches both the S_2 and S_1 monomeric states, reducing their lifetimes by a factor of approximately three, while the lifetime of aggregate species is reduced by a factor of two.

To confirm the lifetimes obtained by time-gated experiments, time-correlated single photon counting (TCSPC) measurements were perfomed in dilute solutions in the presence of molecular oxygen by exciting at both the S_2 and S_1 states (Figure S14). The values obtained (τS_2 = 11.9 ns) showed good agreement with previous lifetime measurements in aerobic conditions, reflecting the reliability of the results.

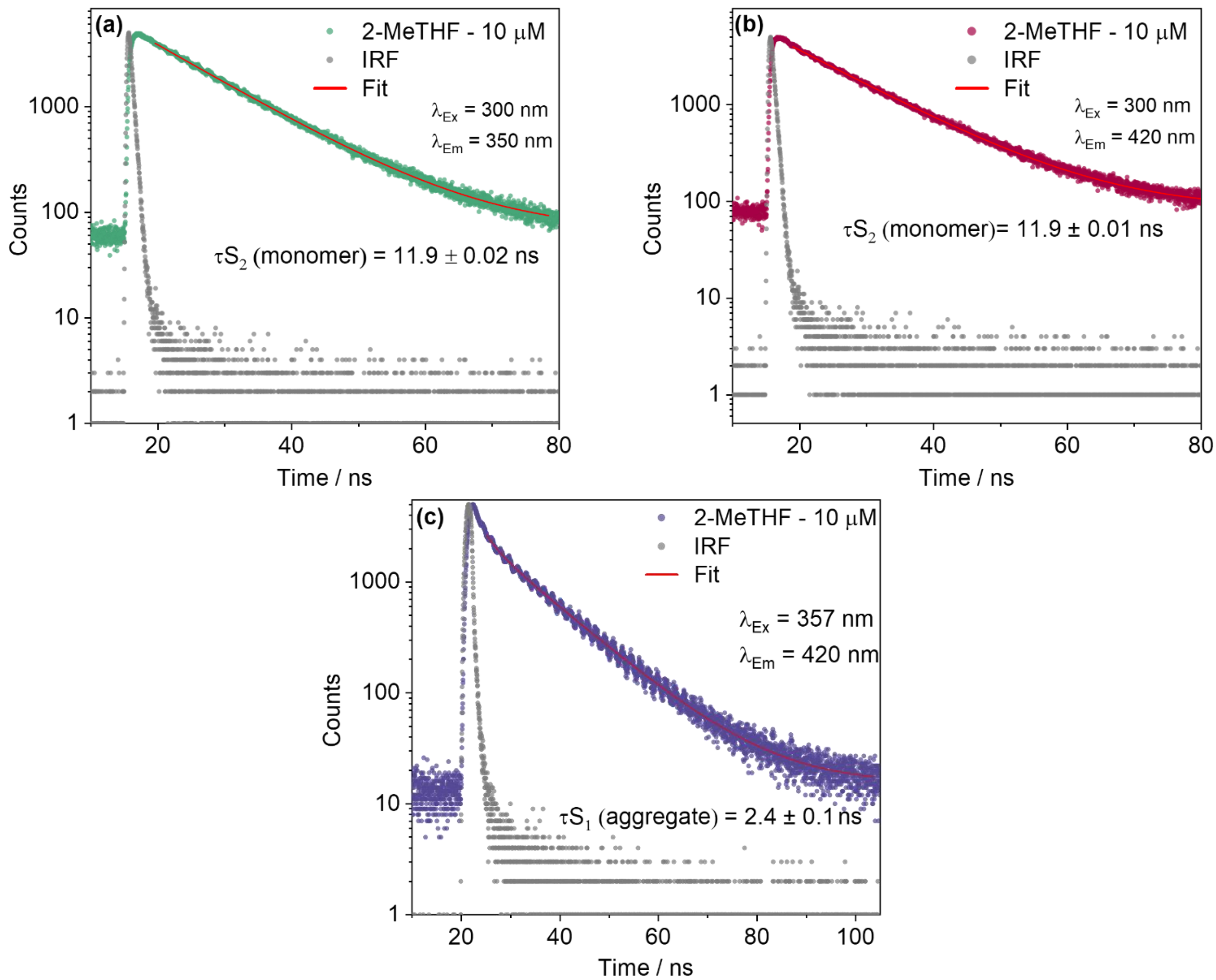

Figure S14. Decay curves of MHTX in 2-methyltetrahydrofuran (2-MeTHF) solution. Prompt fluorescence (PF) decay profiles of **MHTX** (10 µM in 2-MeTHF) at room temperature in the presence of molecular oxygen. **(a)** Decay monitored at λ_{Em} = 350 nm upon excitation of the S2 state (λ_{Ex} = 300 nm). **(b)** Decay monitored at λ_{Em} = 420 nm (λ_{Ex} = 300 nm). **(c)** Decay profile upon selective excitation of the S_1 state (λ_{Ex} = 357 nm) monitored at λ_{Em} = 420 nm. The red solid lines are numerical fits and gray dots indicate the instrument response function (IRF).

4.2. Antisolvent experiment (Figures S15 – S16)

In mixtures with low to moderate water content (1–60%), the S_2 and S_1 emission intensity progressively increases with gradual water addition (Figures S15a and b, respectively). This enhancement is attributed to the formation of a more viscous microenvironment with reduced oxygen solubility, that effectively suppresses the diffusion of molecular oxygen, thereby mitigating singlet quenching in solution (η_{THF}(25 °C) = 0.477 mPa s^{-1} [8]; η_{water}(25 °C) = 0.890 mPa s^{-1} [9]). To validate this assignment, key mixtures were analyzed after purging the solution with argon (Figure S15c), showing no significant change in emission intensity for the 0–60% fractions under deoxygenated conditions. This stability suggests that up to 60%, the strong THF-**MHTX** interaction forms a protective solvation shell, effectively hindering molecular aggregation by water addition. At a critical water fraction of 70%, the monomeric S_2 emission intensity decreases sharply, concurrent with the emergence of a distinct, vibronically resolved fluorescence band (Figure S15a). This new feature exhibits a 4 nm (0.03 eV) bathochromic shift relative to the monomeric S_1 emission (Figure S15b), signaling the onset of formation of a weak intermolecular coupling dimer, herein called "loose dimer" that emits exclusively from its S_1 state. Upon further increasing the water content above 80%, the system enters a fully aggregated state that accompanies the complete quenching of both S_2 and S_1 monomeric fluorescence, as the emissive profile becomes dominated by the loose dimer emission.

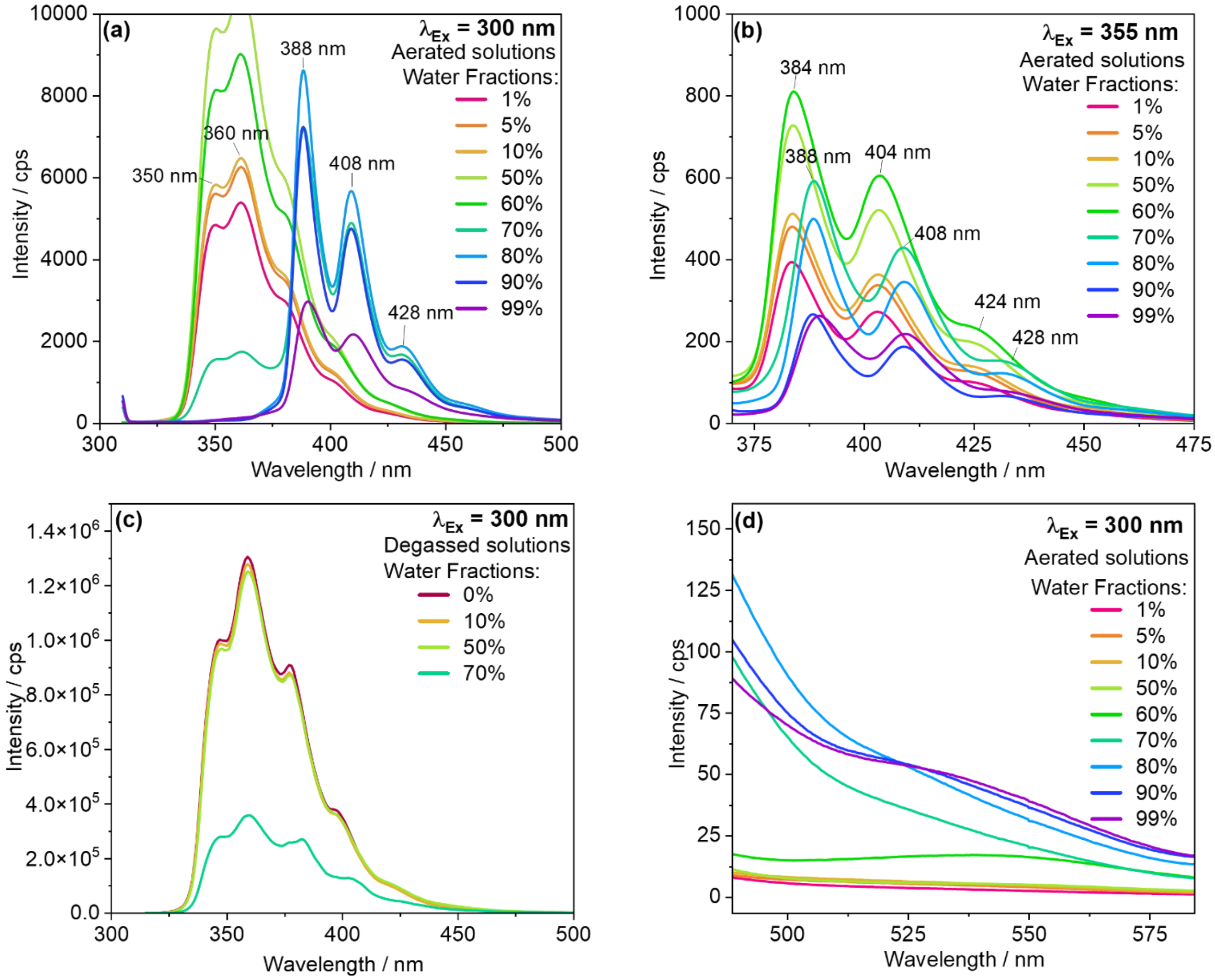

Figure S15. Effect of water content of the photophysics of MHTX. Steady-state photoluminescence and excitation spectra of **MHTX** in THF/water mixtures with varying water fractions (f_w) maintaining a constant total concentration of 20 μM. **(a)** PL spectra (λ_{Ex} = 300 nm) showing the stability of the dual S_2 emission profile up to f_w = 60%. **(b)** Emission spectra (λ_{Ex} = 355 nm) selectively exciting the S_1 state. **(c)** Emission spectra (λ_{Ex} = 300 nm) in degassed solutions, highlighting the intensity enhancement upon oxygen removal. **(d)** PL spectra (λ_{Ex} = 300 nm) showing the emission band from a low-energy aggregate at f_w>50%.

To investigate their absorption characteristics, excitation spectra were collected at various emission wavelengths during the antisolvent experiment. The excitation spectra monitored at 360 nm (Figure S16a) exhibited a substantial decrease in intensity at 70% water content, followed by a complete quenching at fractions above 80%. These observations confirm two key points: first, the higher-energy emission band is solely related to monomeric species, and second, the system reaches a state of complete aggregation above 80% water content. When collected at 400 nm, where both species emit from their S_1 state, (Figure S16b) a slight red-shift is observed above 80% water fraction, similar to the red-shift obtained in the emission spectra (see Figure S12a). The shifted excitation spectrum develops new excitation bands at wavelengths longer than 365 nm, attributable exclusively to aggregate species. Finally, when monitored at 450 nm, a region specific to aggregate emission (Figure S16c), the excitation intensity increased substantially with higher water content, confirming that emission at this wavelength originates directly from the aggregates, and that their absorption profile is similar to monomeric species.

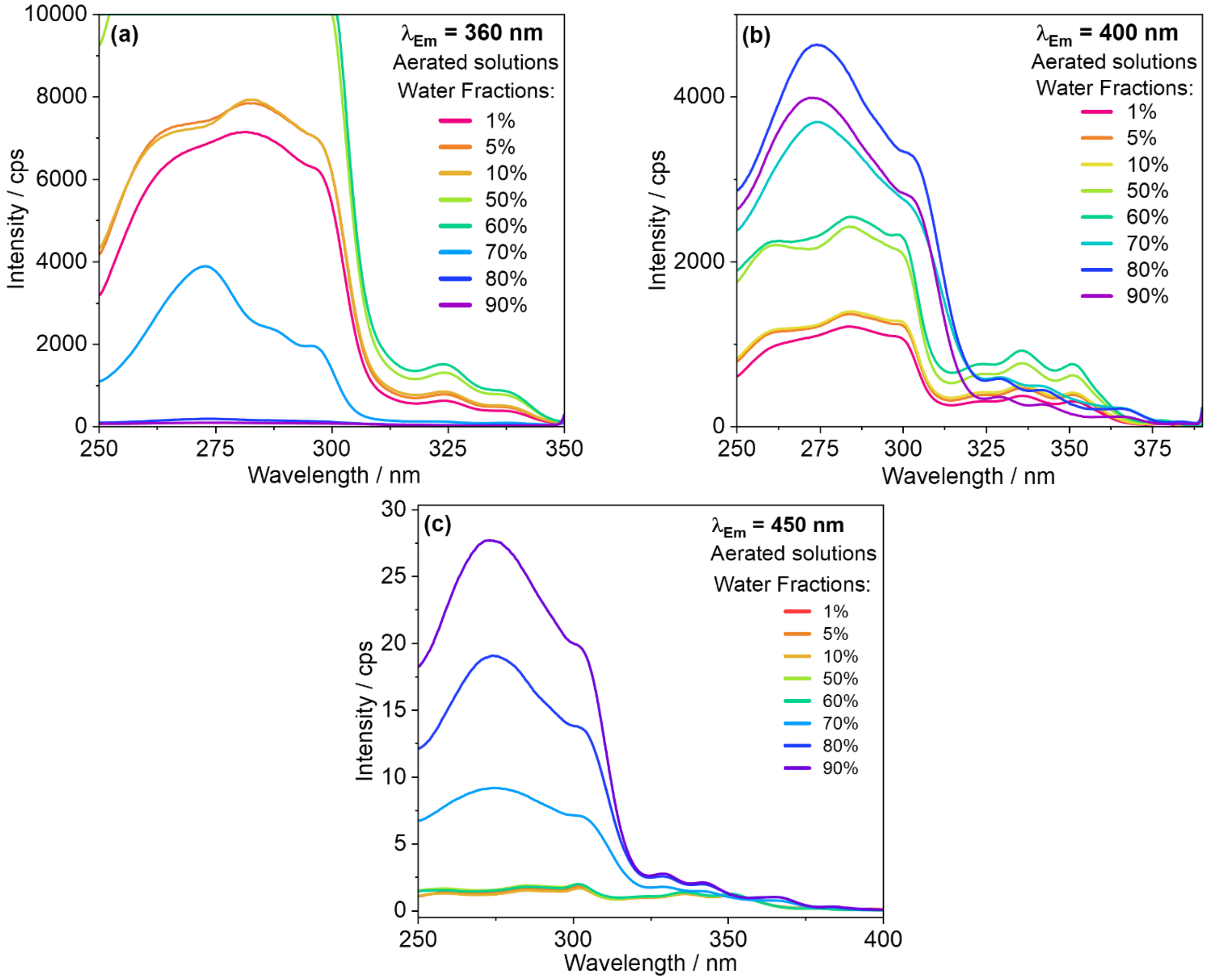

Figure S16. Effect of water content of the photophysics of MHTX. Excitation spectra of **MHTX** in aerated THF/water mixtures with varying water fractions (f_w) maintaining a constant total concentration of 20 μM. Emission collected at **(a)** 360 nm **(b)** 400 nm and **(c)** 450 nm.

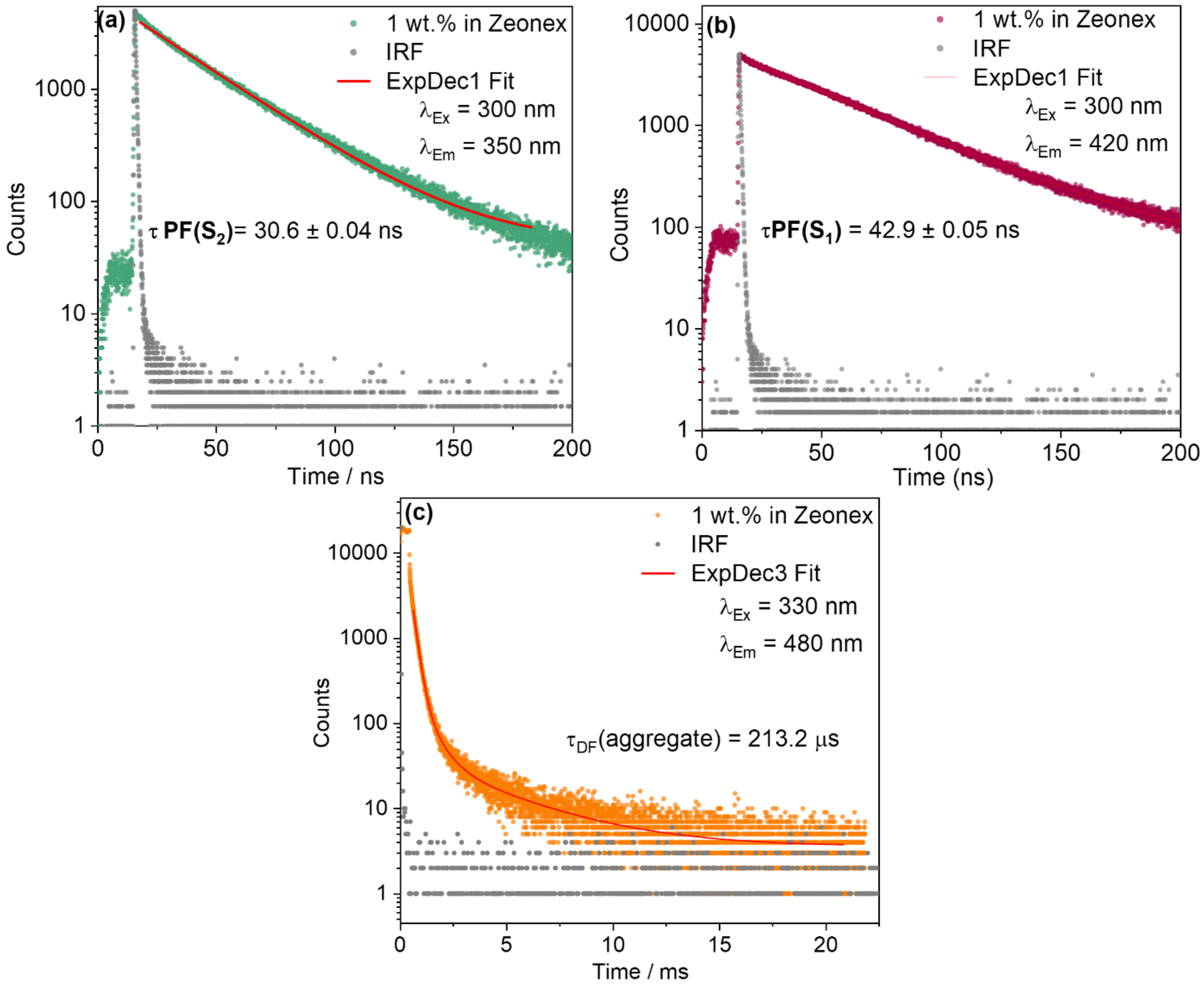

Figure S17. Decay curves of MHTX doped into guest-host film. Excited-state kinetics in the solid state. Time-correlated single photon counting (TCSPC) decay curves of **MHTX** (1 wt.% in Zeonex) in the presence of oxygen (T = 298 K). **(a)** Decay of the monomeric anti-Kasha S_2 emission recorded at λ_{Em} = 350 nm. **(b)** Decay of the S_1 emission recorded at λ_{Em} = 420 nm. Both states were addressed upon excitation at λ_{Ex} = 300 nm. **(c)** Decay of the aggregate TTA recorded at λ_{Em} = 480 nm under 330 nm excitation. Gray scatter points represent the instrumental response function (IRF).

Dual delayed emissions of MHTX

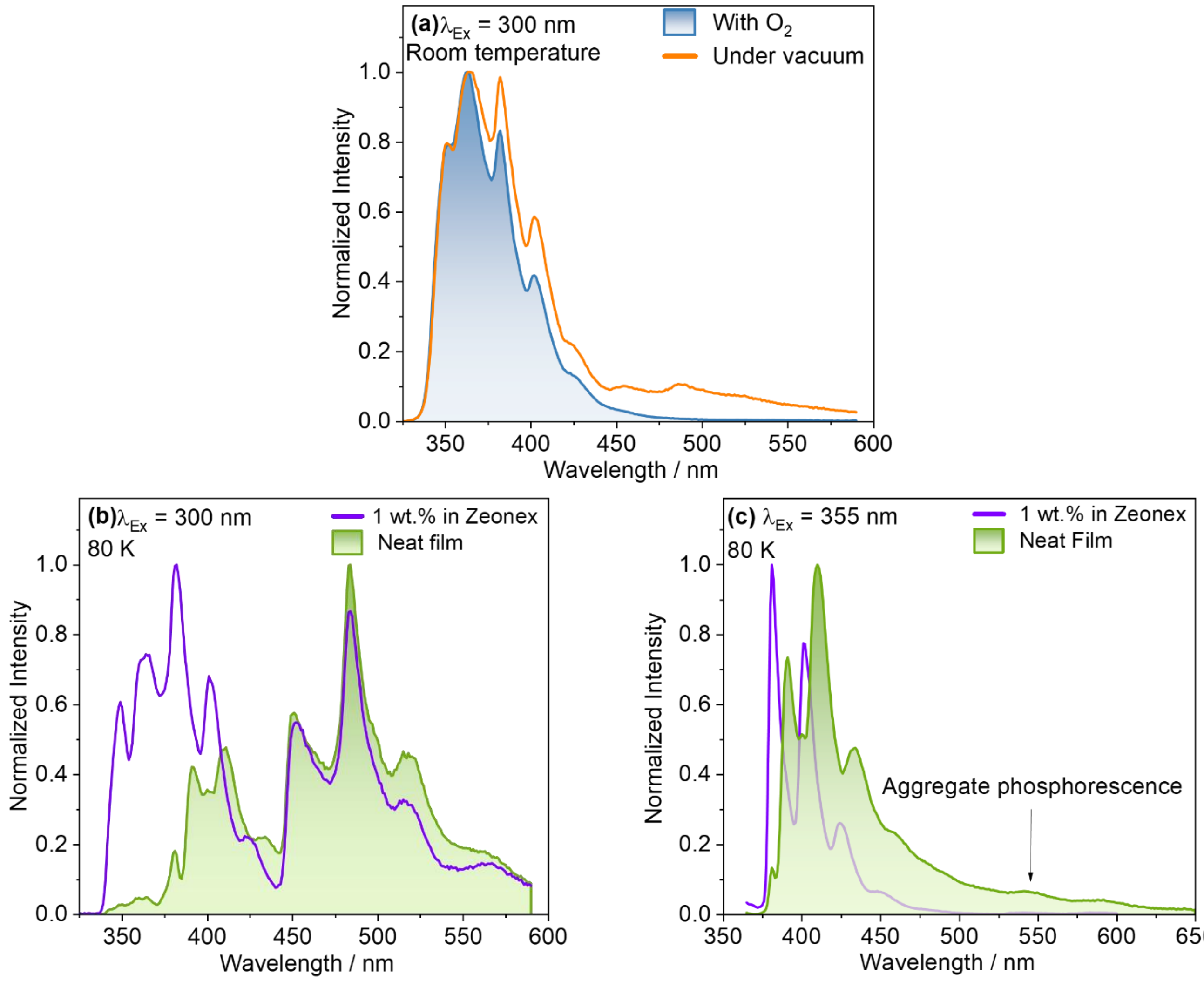

Figure S18. Behavior of MHTX in solid-state. Steady-state emission spectra of **MHTX** in the solid state under varying conditions. (a) Emission at room temperature (298 K) in 1-wt.%-in-Zeonex drop-cast film, under vacuum (orange line) and in air (shaded blue) with excitation at λ_{Ex} = 300 nm. (b and c) Low-temperature (80 K) emission comparing a dilute matrix (1 wt.% in Zeonex, purple line) to a neat film (shaded green) upon excitation at λ_{Ex} = 300 nm (b); and λ_{Ex} = 355 nm (c). The arrow in panel (c) indicates the emergence of a broad aggregate phosphorescence band at longer wavelengths in the neat film.

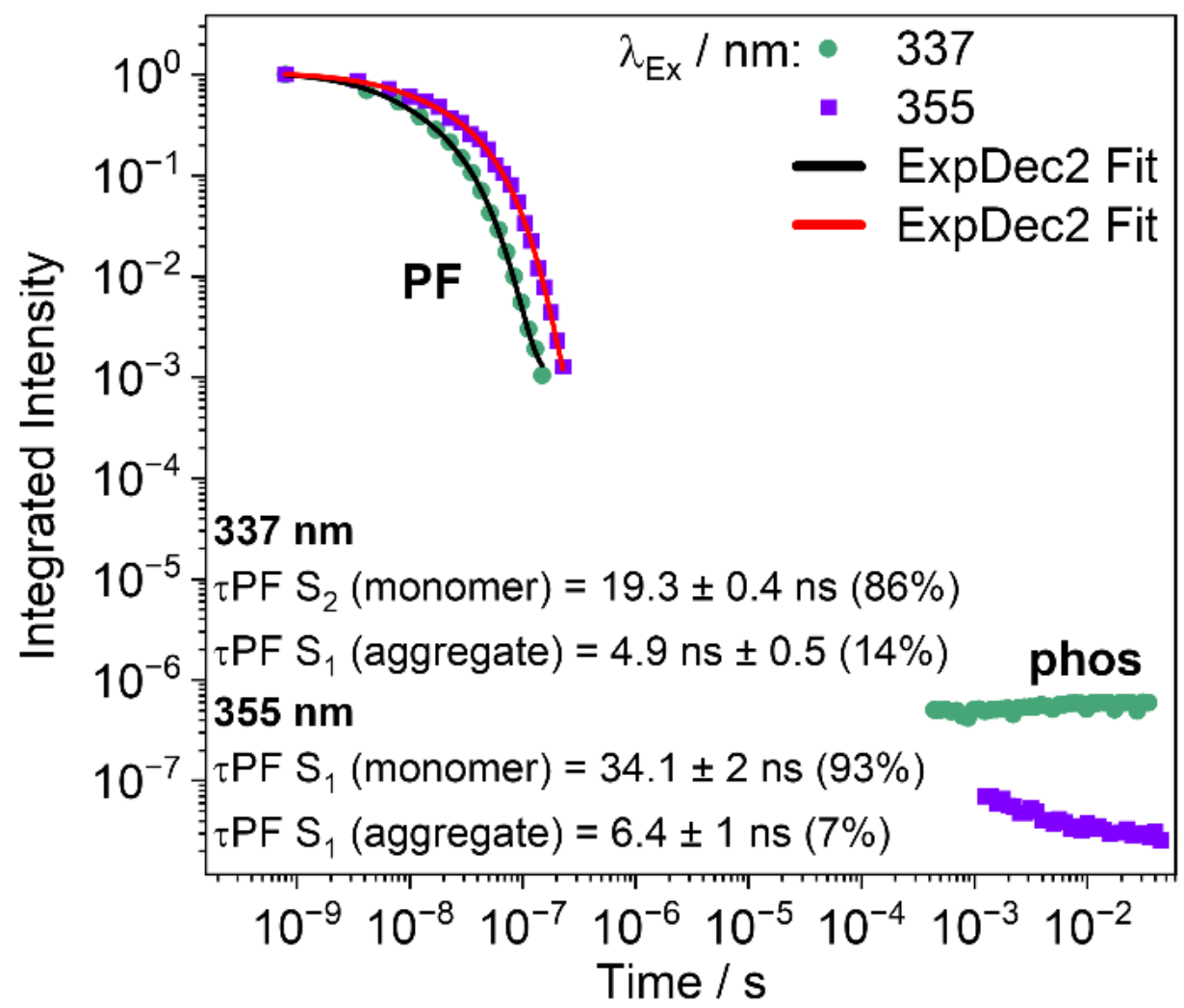

Figure S19. Integrated photoluminescence intensity as a function of time obtained from time-gated emission spectra at 80 K (Figure 3e and f) upon excitation at: 337 nm, green circles, 0.5 wt.% in Zeonex; and 355 nm, purple squares, 0.1 wt.% in Zeonex.

4.3 Room-temperature time-resolved photoluminescence in solid-state films

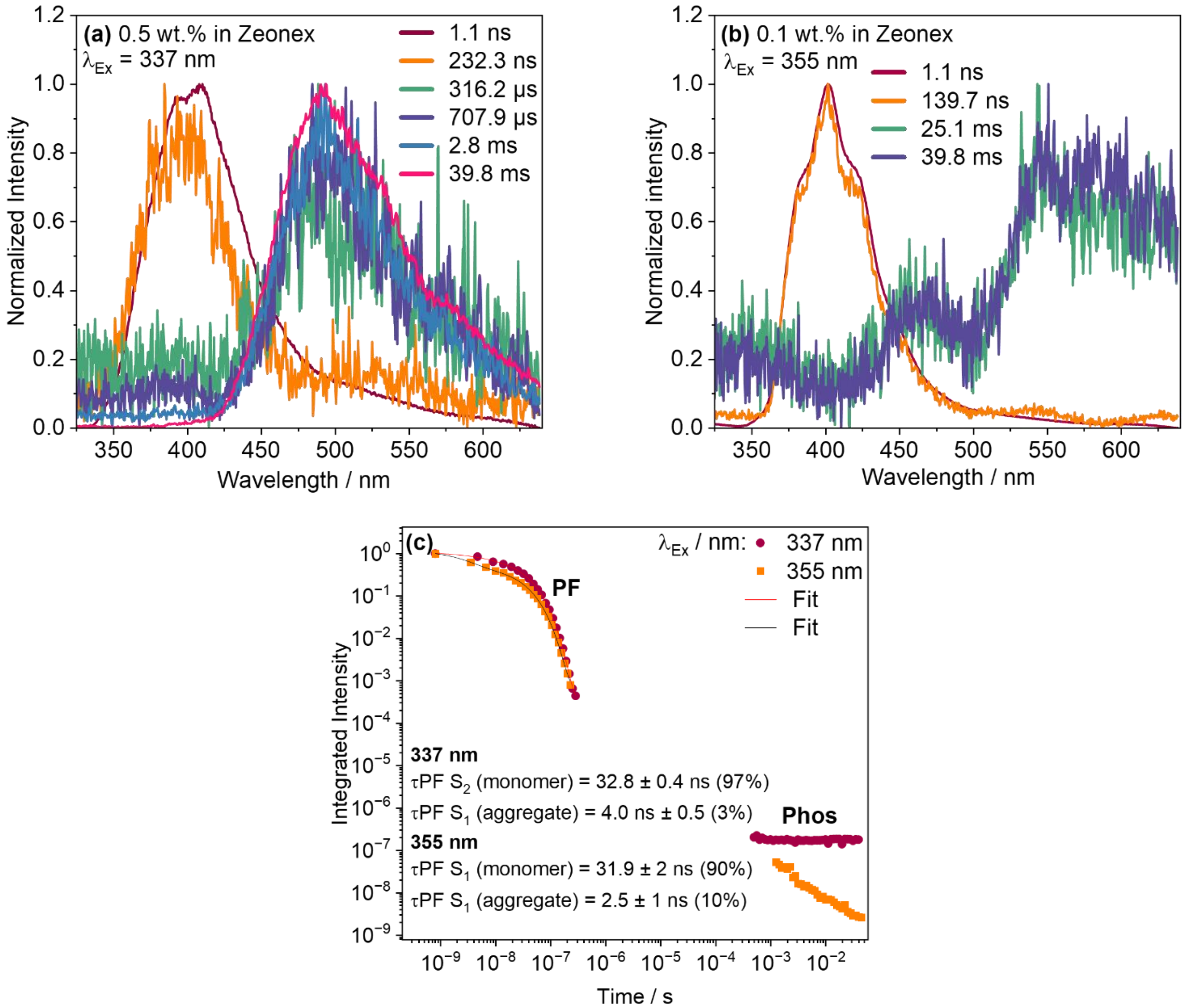

Figure S20. Time-resolved photoluminescence of MHTX doped into guest-host film. Room-temperature time-resolved photoluminescence in solid-state films. (a–b) Time-gated emission spectra of **MHTX** in Zeonex films at room temperature (298 K), recorded at indicated time delays following excitation at **(a)** 337 nm (0.5 wt.% film) and **(b)** 355 nm (0.1 wt.% film). **(c)** Integrated photoluminescence intensity as a function of time for both excitation wavelengths (337 nm, dark red circles; 355 nm, orange squares).

4.4 Low-temperature time-resolved photoluminescence in solution

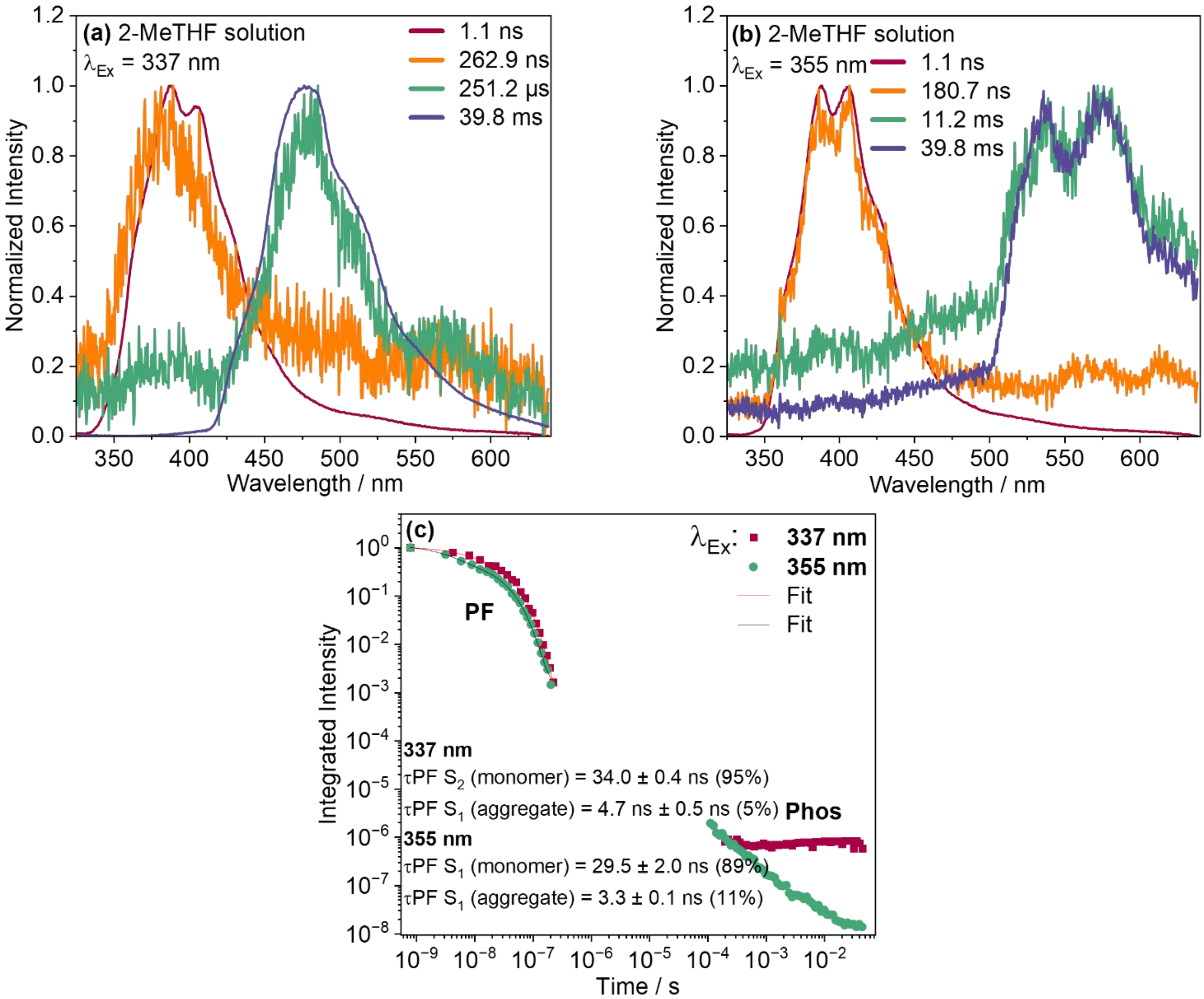

Figure S21. Low-temperature time-resolved photoluminescence in dilute solution. (a–b) Time-gated emission spectra of **MHTX** (10 µM in 2-MeTHF) at 80 K recorded at various delay times following excitation at **(a)** 337 nm and **(b)** 355 nm. **(c)** Integrated photoluminescence intensity as a function of time (decay profile) for both excitation wavelengths (337 nm, dark red squares; 355 nm, green circles).

4.5 Time-resolved photoluminescence of MHTX as a neat film

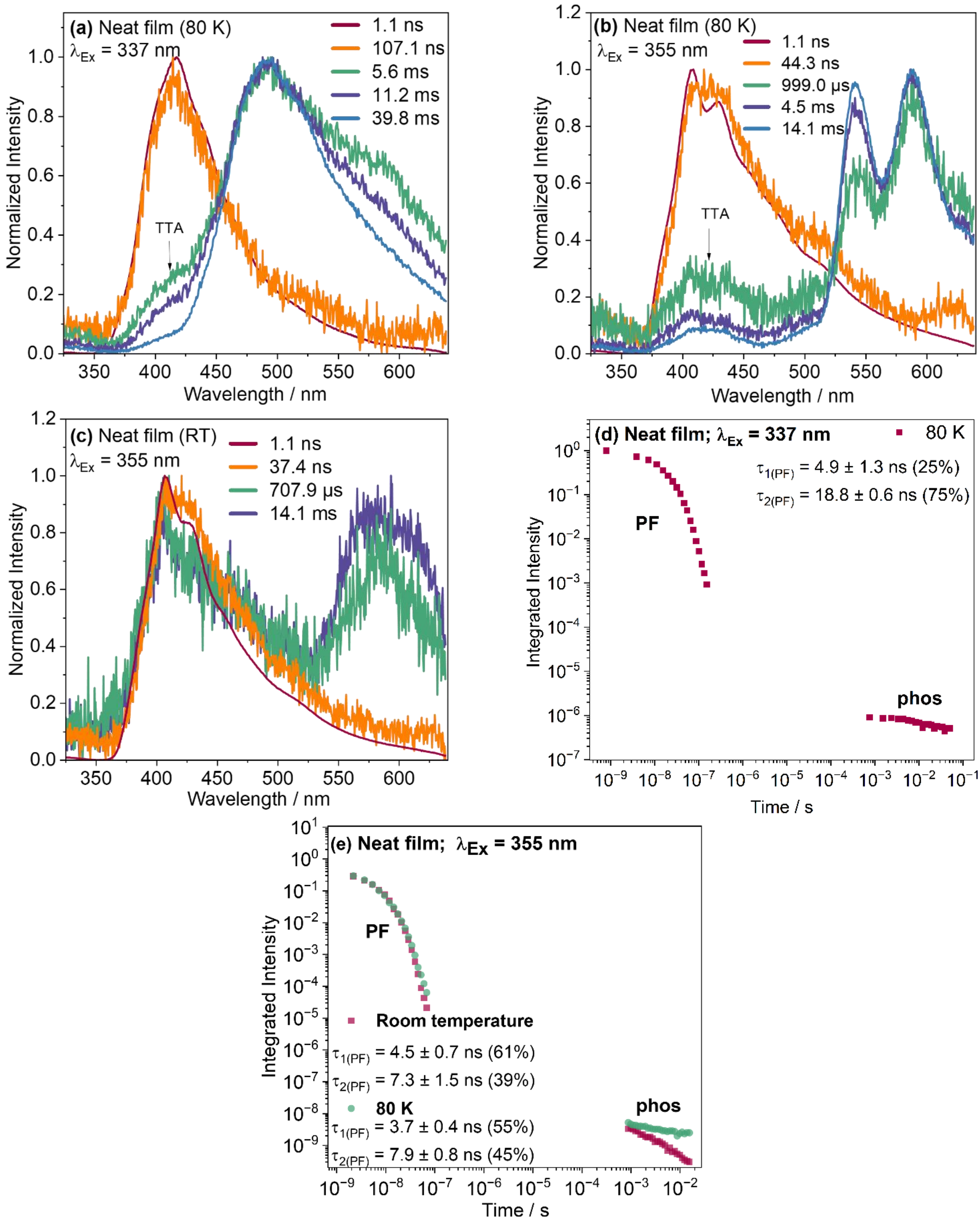

Figure S22. Time-resolved photoluminescence of MHTX in neat film. (a–b) Time-gated emission spectra of a neat film at 80 K upon excitation at (a) 337 nm and (b) 355 nm. Arrows indicate the contribution of triplet-triplet annihilation (TTA). **(c)** Time-gated emission of a neat film at room temperature (298 K) following 355 nm excitation. (d–e) Integrated photoluminescence intensity decays for the neat film upon excitation at: **(d)** 337 nm and **(e)** 355 nm. The later measured at both room temperature (blue squares) and 80 K (green circles).

Table S3. Prompt fluorescence lifetimes (τ) and amplitude-weighted average pre-exponential factors (A) for samples of **MHTX** in solution and drop-cast films, at both room temperature and 80 K. Data were acquired using time-gated measurements upon 337 and 355 nm excitation under vacuum.

Conditions	Lifetime[a]	λ_{Ex} = 337 nm / ns (A)[b]	λ_{Ex} = 355 nm / ns (A)
10 μM in 2-MeTHF (Room temperature) Aerated	τ(monomer)	19.1 (26%)	10.6 (82%)
	τ(aggregate)	7.9 (74%)	2.3 (18%)
10 μM in 2-MeTHF (Room temperature) Degassed	τ(monomer)	33.5 (94%)	32.9 (95%)
	τ(aggregate)	5.2 (6%)	4.9 (5%)
0.1 μM in 2-MeTHF (80 K)	τ(monomer)	34.0 (95%)	29.5 (94%)
	τ(aggregate)	4.7 (5%)	3.3 (6%)
0.1 wt.% in Zeonex (Room temperature)	τ(monomer)	-[c]	31.9 (90%)
	τ(aggregate)		2.5 (10%)
0.1 wt.% in Zeonex (80 K)	τ(monomer)	-[c]	34.1 (93%)
	τ(aggregate)		6.4 (7%)
0.5 wt.% in Zeonex (Room temperature)	τ(monomer)	32.8 (97%)	-[c]
	τ(aggregate)	4.0 (3%)	
0.5 wt.% in Zeonex (80 K)	τ(monomer)	19.3 (86%)	-[c]
	τ(aggregate)	4.9 (14%)	
Neat film (Room temperature)	τ(monomer)	-[c]	7.3 (39%)
	τ(aggregate)		4.5 (61%)
Neat film (80 K)	τ(monomer)	18.8 (75%)	7.9 (45%)
	τ(aggregate)	4.9 (25%)	3.7 (55%)

[a] Obtained by a biexponential fitting of the time-gated decays.
[b] Indicative of the relative contribution of each decay component.
[c] Conditions not measured.

Table S4. Time-correlated single photon counting (TCSPC) measurements for **MHTX** in solution and drop-cast films, at room temperature in the presence of oxygen. Data were acquired according to the conditions mentioned below. Aggregate TTA lifetime was measured in Zeonex film (1 wt.%) under vacuum.

Conditions	λ_{Ex} / nm	λ_{Em} / nm	Lifetime
10 μM in 2-MeTHF (Room temperature) Aerated	300	350	11.9 ns[a]
	300	420	11.9 ns[a]
	357	420	2.4 ns[a]
1 wt.% in Zeonex (Room temperature) Aerated	300	350	30.6 ns[a]
	300	420	42.9 ns[a]
	357	500	110.9 ms[b]

[a] Prompt fluorescence lifetimes
[b] Aggregate TTA lifetime

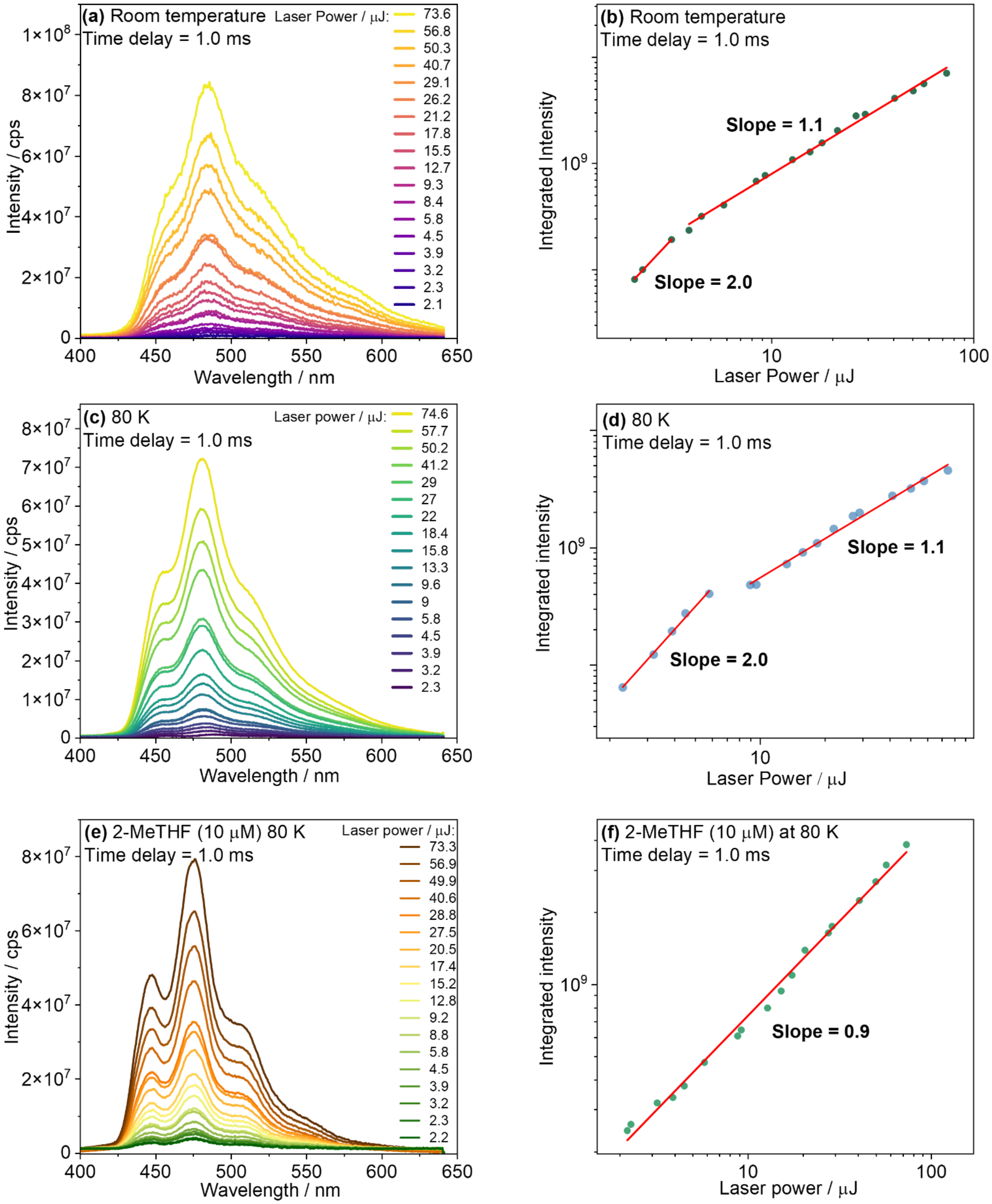

Figure S23. Time-resolved photoluminescence spectra (left column) and power-dependent plots (right column) of **MHTX** under vacuum (λ_{Ex} = 337 nm) and different conditions: **(a)** and **(b)** measurement at 298 K for a 1 wt.% Zeonex drop-cast film; **(c)** and **(d)** at 80 K for a 1 wt.% Zeonex drop-cast film. **(e)** and **(f)** at 80 K for a 10 µM solution in 2-MeTHF. The time-resolved spectra were collected with a time delay (TD) of 1.0 ms and an integration window of 10 ms.

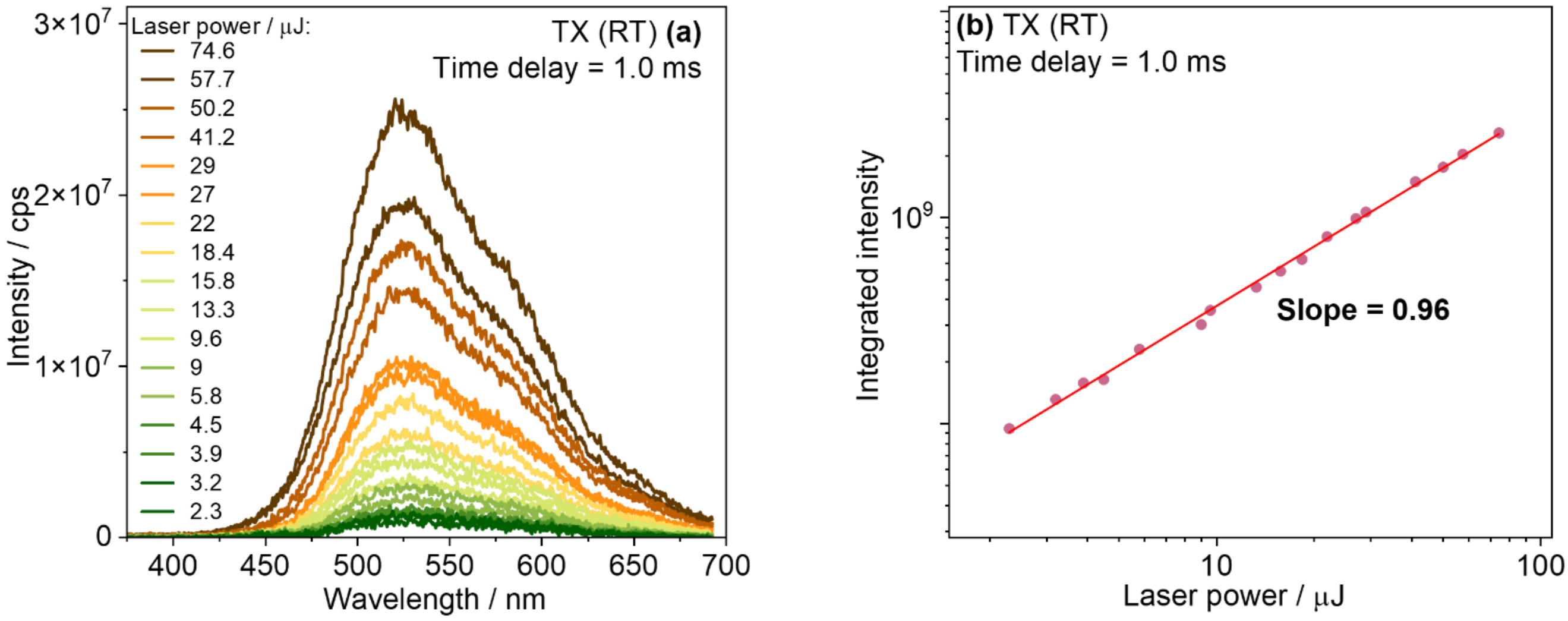

Figure S24. Investigation of long-lived emission of truxene. Time-resolved photoluminescence spectra (left) and power-dependent plot (right) of truxene **(TX)** under vacuum (λ_{Ex} = 337 nm) at 298 K for a 1 wt.% Zeonex drop-cast film.

5 DFT Calculations

Table S5. Franck-Condon excited states at the S_0 optimized structure. Major configurations are shown where CI coefficients exceed 0.3. If no configuration meets this threshold, those with coefficients greater than 0.24 are presented.

State	Excitation Energy /eV	/nm	Oscillator Strength	Major Configurations (CI Coefficients)
T_1	3.071	403.8	-	HO→LU+1 (-0.3464), HO→LU (0.4746)
T_2	3.438	360.6	-	HO-1→LU (-0.3633), HO→LU+1 (0.3526)
T_3	3.451	359.3	-	HO-1→LU (0.3233), HO-1→LU+1 (0.4065), HO→LU (0.3526)
T_4	3.775	328.4	-	HO-1→LU (0.4017), HO→LU+1 (0.4376)
S_1	3.907	317.4	0.0012	HO-1→LU (0.4289), HO→LU+1 (0.4515)
T_5	3.984	311.2	-	HO-2→LU (0.3721), HO→LU+2 (0.3093)
T_6	4.034	307.3	-	HO-2→LU+1 (0.3140), HO-1→LU+2 (-0.3009)
T_7	4.244	292.1	-	HO-5→LU+3 (-0.2391), HO-4→LU+4 (0.2416)
T_8	4.296	288.6	-	HO→LU+3 (0.4260)
T_9	4.338	285.8	-	HO-3→LU (0.2972), HO-1→LU+3 (-0.2497), HO-1→LU+5 (0.2404)
T_{10}	4.358	284.5	-	HO-4→LU (0.2875), HO-3→LU+1 (-0.2846)
S_2	4.413	281.0	0.3503	HO-1→LU (-0.3844), HO→LU (0.5575)
T_{11}	4.446	278.9	-	HO-4→LU+3 (0.3213)
S_3	4.475	277.0	0.6826	HO-1→LU (-0.3653), HO→LU+1 (0.4644)
T_{12}	4.476	277.0	-	HO-3→LU+5 (0.2794), HO-2→LU+1 (-0.2518)
S_4	4.553	272.3	0.4803	HO-1→LU+1 (0.6351)

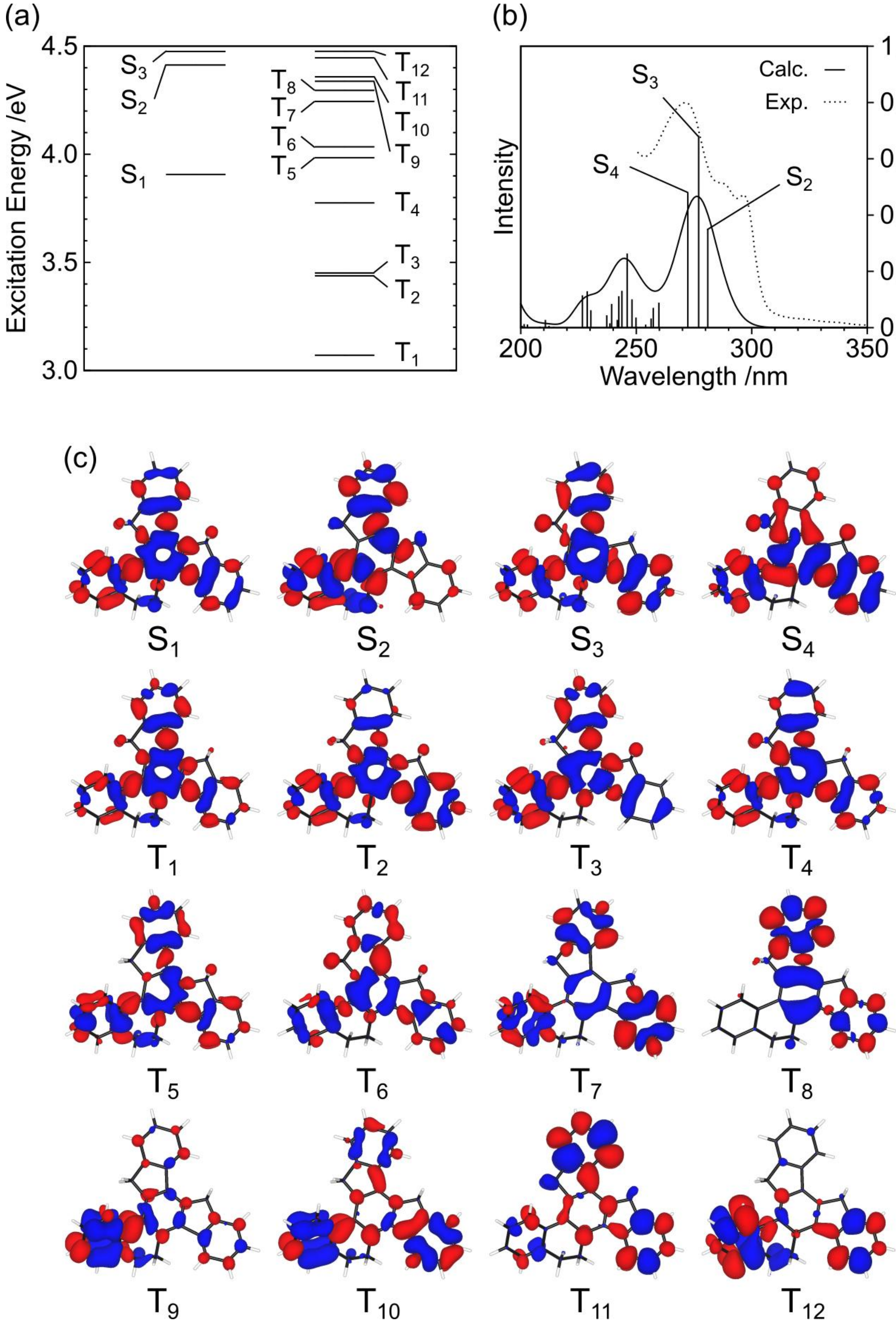

Figure S25. Theoretical calculations of MHTX at ground state. (a) Energy levels of the Franck-Condon excited states at the S_0 optimized structure. (b) Calculated UV/Vis spectrum obtained by broadening the oscillator strengths using a Gaussian function with a linewidth of 1000 cm^{-1}. (c) Electron density differences for S_n-S_0 and T_n-T_0 at the S_0 optimized structure (isosurface value: 5.0×10^{-4} a.u.). Red and blue regions indicate positive and negative values, respectively. (a. u. = atomic units)

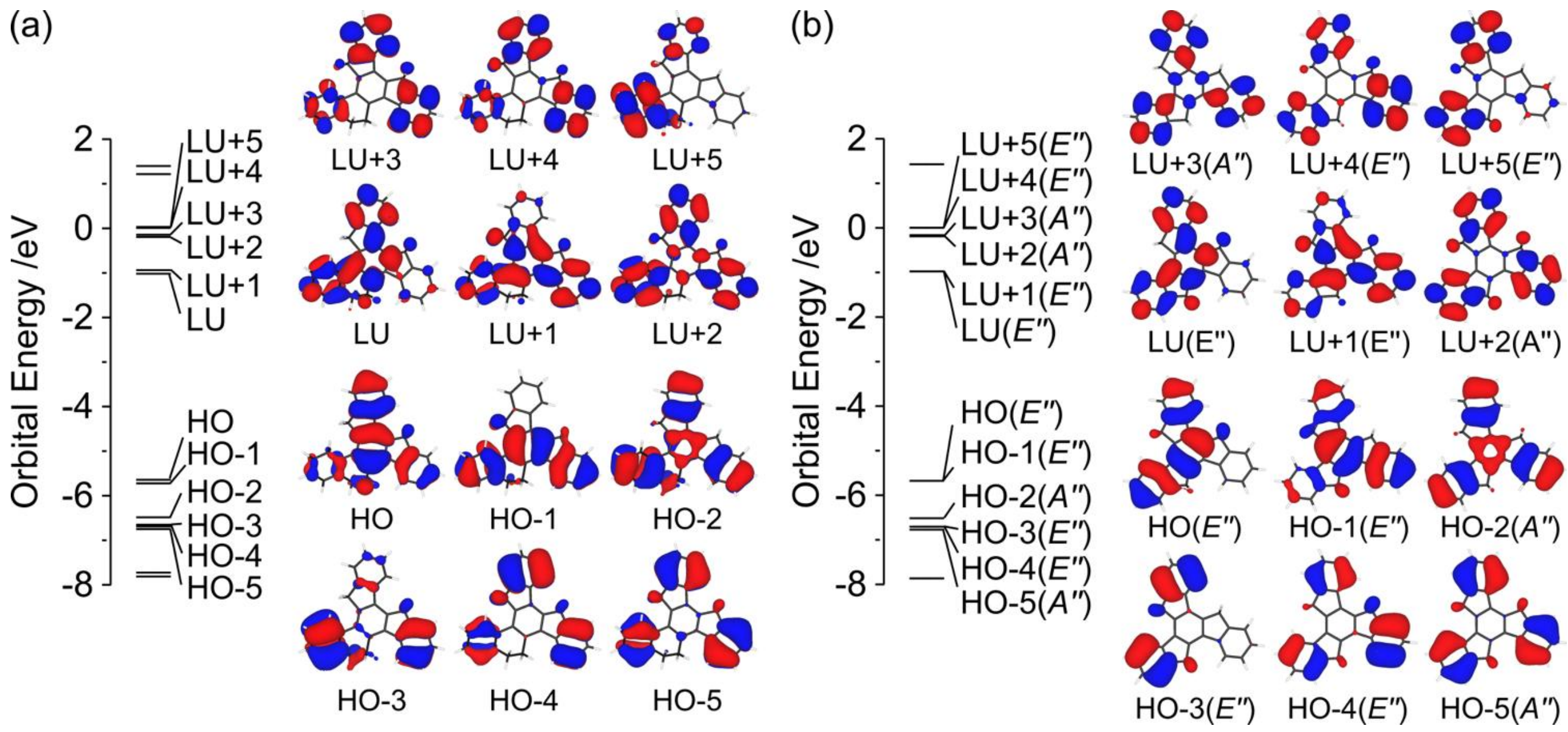

Figure S26. Calculated energy levels and molecular orbitals for MHTX and truxene. Orbital energy levels and molecular orbitals (isosurface value: 2.0×10^{-2} a.u.) at the S_0 optimized structure for (a) **MHTX** (C_1 symmetry) and (b) **TX** (C_{3h} symmetry). (a. u. = atomic units)

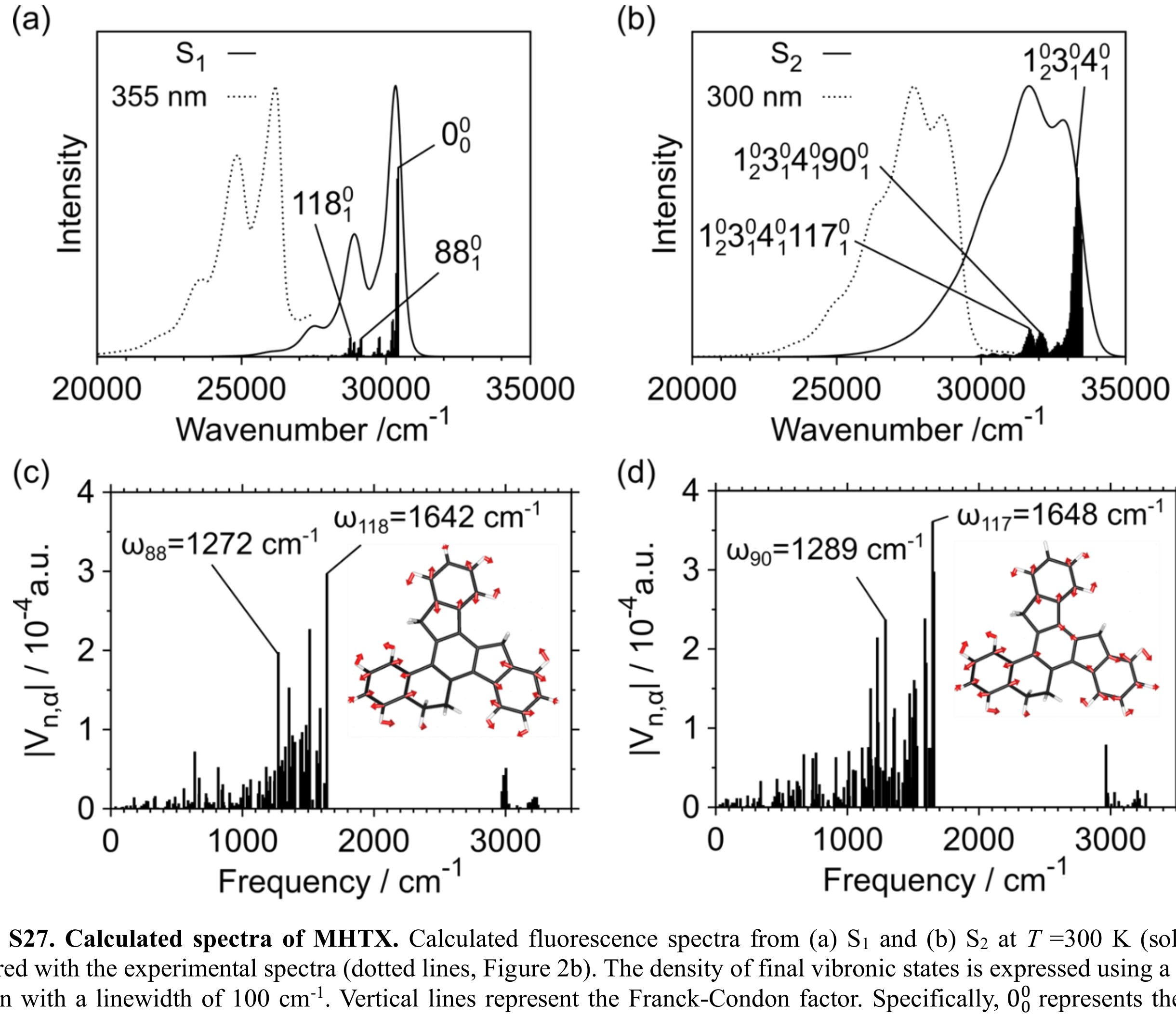

Figure S27. Calculated spectra of MHTX. Calculated fluorescence spectra from (a) S_1 and (b) S_2 at T =300 K (solid lines), compared with the experimental spectra (dotted lines, Figure 2b). The density of final vibronic states is expressed using a Gaussian function with a linewidth of 100 cm^{-1}. Vertical lines represent the Franck-Condon factor. Specifically, 0^0_0 represents the Franck-Condon factor for the 0–0 transition, and $\alpha^{\nu}_{\nu'}$ represents the Franck-Condon factor between initial ν and final ν' vibrational quantum numbers for vibrational mode α. Diagonal vibronic coupling constants of (c) $S_0@S_1$ with vibrational mode 118 and (d) $S_0@S_2$ with vibrational mode 117 calculated within the crude adiabatic approximation [10]. (a. u. = atomic units)

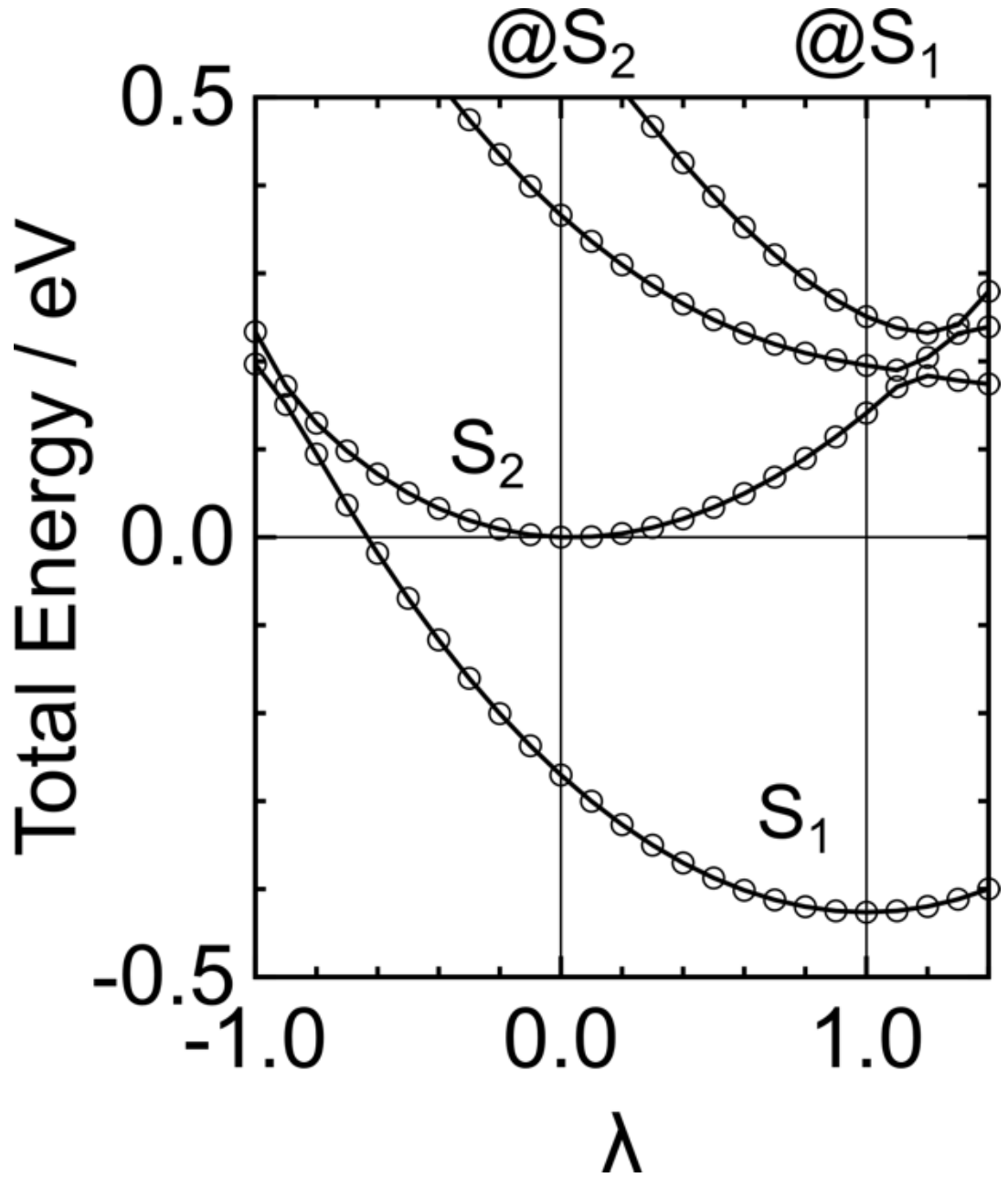

Figure S28. Investigation of S_1 and S_2 state of MHTX. Potential energy curves connecting the optimized S_2 and S_1 geometries: $E(R_\lambda)$ with $R_\lambda = (1-\lambda)R_{@S_2} + \lambda R_{@S_1}$, where $\boldsymbol{R}_{@S_2}$ and $\boldsymbol{R}_{@S_1}$ represent the Cartesian coordinates at the S_2 and S_1 geometries, respectively. The energy reference is the total energy of S_2@S_2 (-1078.9610 a.u.). (a. u. = atomic units)

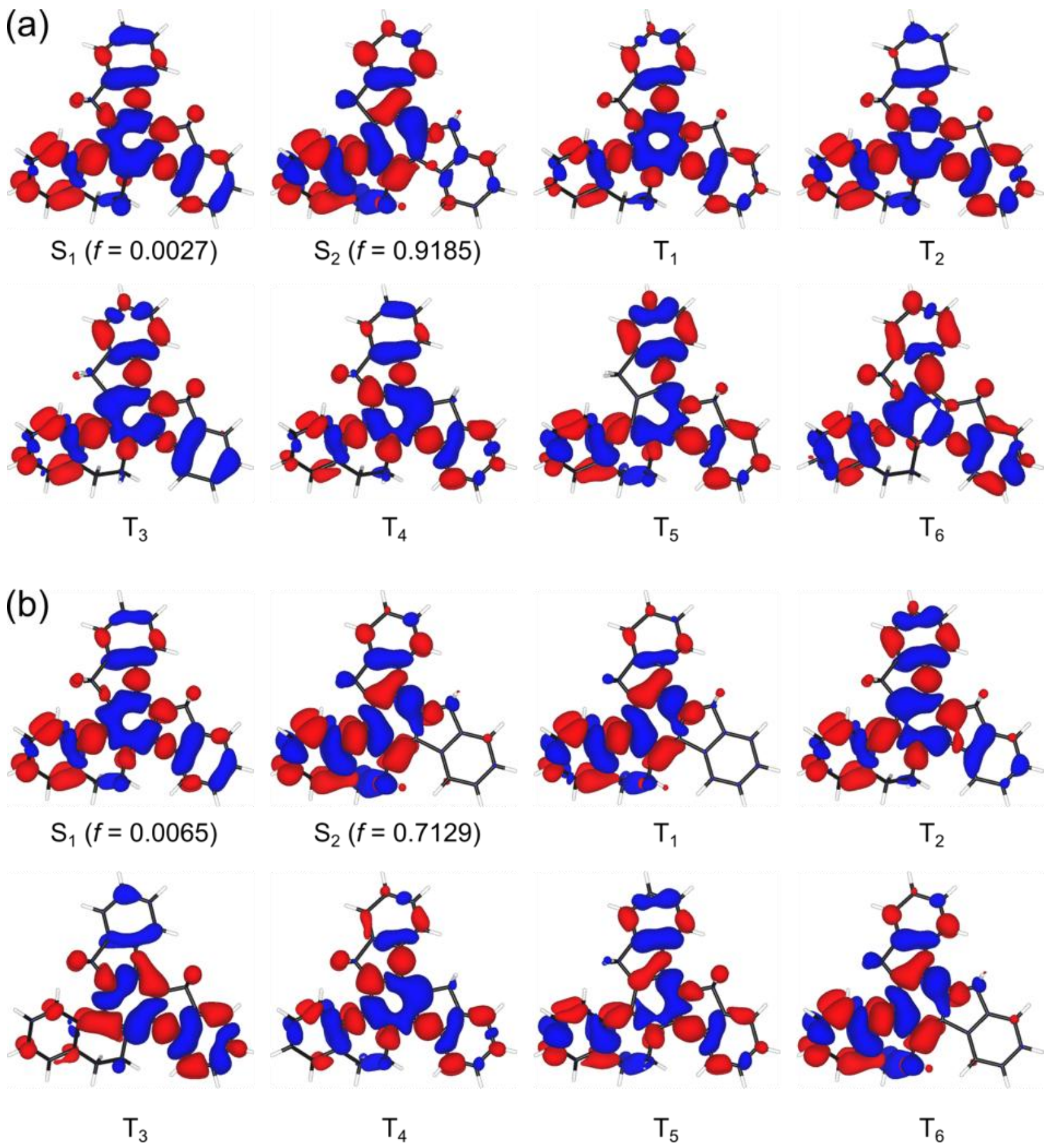

Figure S29. Electronic density of radiative decays of MHTX. Electron density differences for S_m-S_0 and T_m-T_0 at the (a) S_1 and (b) S_2 optimized structures (isosurface value: 5.0×10^{-4} a.u.). Red and blue regions indicate positive and negative values, respectively. (a. u. = atomic units)

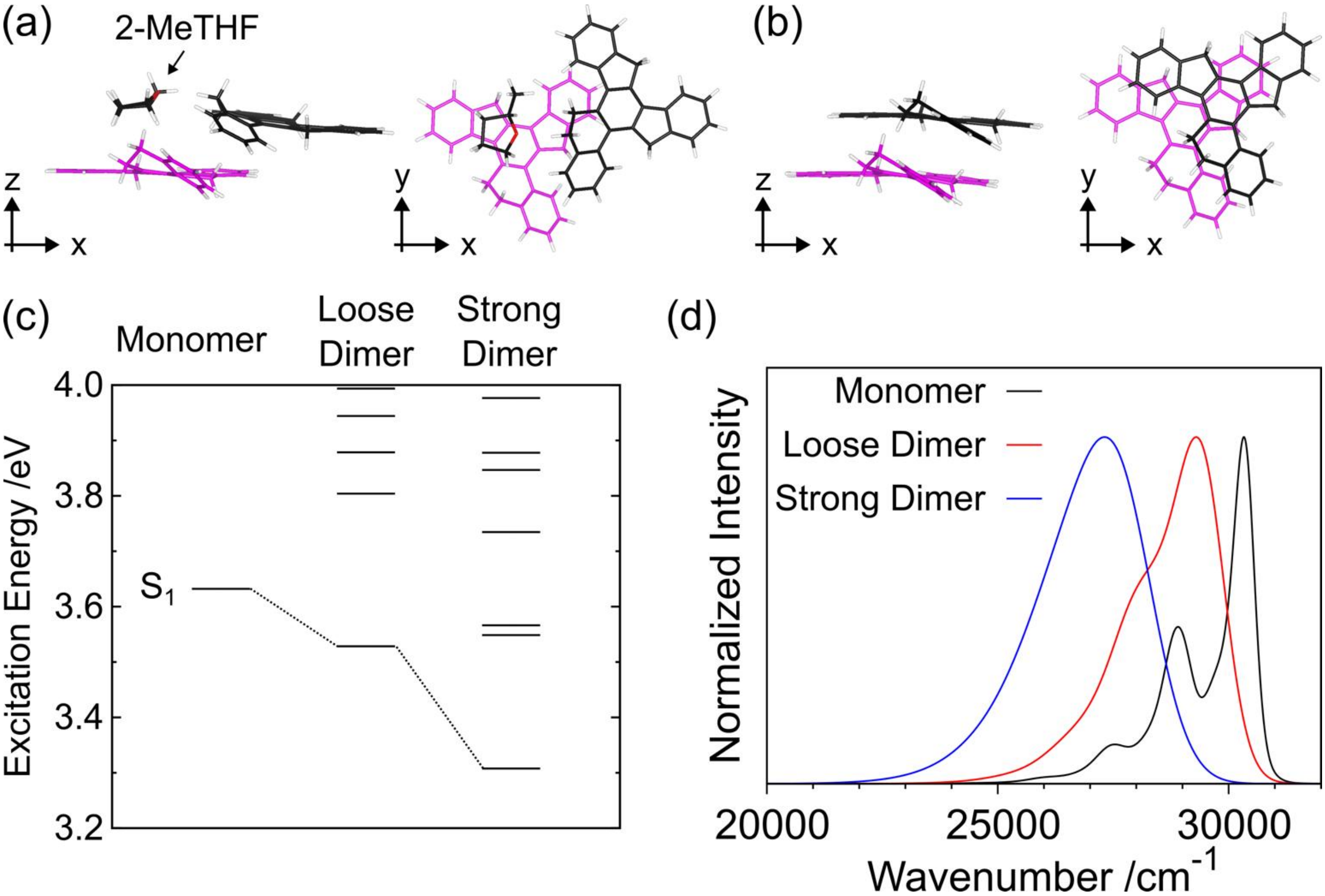

Figure S30. Investigation of dimer formation. S_1 optimized geometries of the (a) loose and (b) strong dimers. (c) Calculated energy levels of excited states at the S_1 optimized structure and (d) S_1 fluorescence spectra for the monomer, loose dimer, and strong dimer. The density of final vibronic states is expressed using a Gaussian function with a linewidth of 100 cm^{-1} for the spectral calculations.

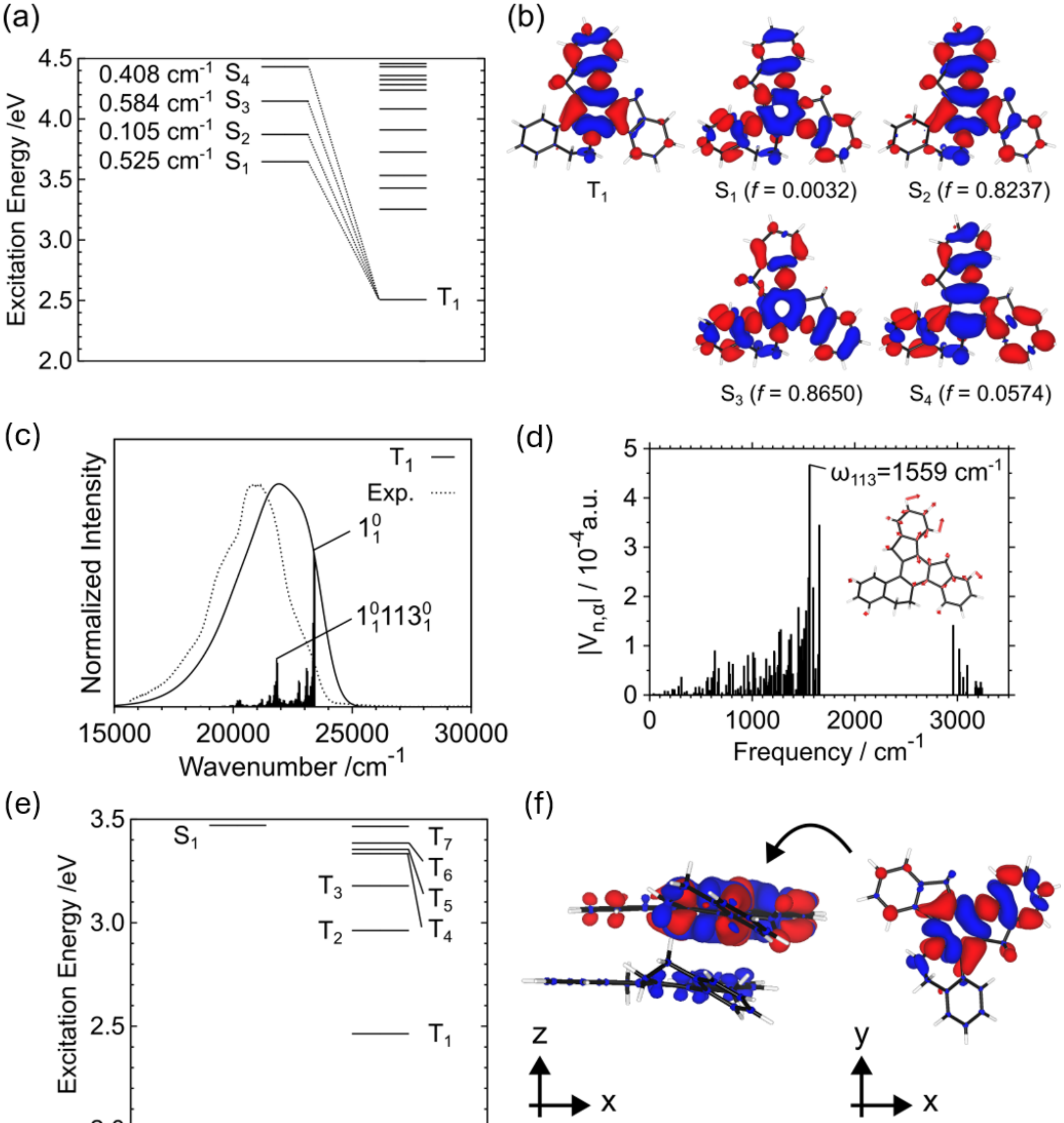

Figure S31. Theoretical study of the triplet states of MHTX. (a) Energy levels of excited states at the T_1 optimized structure for the monomer. Values represent the SOC matrix element between singlets and triplets. (b) Electron density differences for S_m-S_0 and T_m-T_0 at the T_1 optimized structure for the monomer (isosurface value: 5.0×10^{-4} a.u.). Red and blue regions indicate positive and negative values, respectively. f represents the oscillator strength of S_m-S_0. (c) Calculated monomer phosphorescence spectrum from T_1 at T =80 K (solid line), compared with the experimental spectrum (dotted line, Figure S19b). The density of final vibronic states is expressed using the Gaussian function with a linewidth of 600 cm^{-1}. Vertical lines represent the Franck-Condon factor, where $\alpha^{\nu}_{\nu'}$ represents the Franck-Condon factor between initial ν and final ν' vibrational quantum numbers for vibrational mode α. (d) Diagonal vibronic coupling constants of S_0@T_1 with vibrational mode 113 calculated within the Born-Oppenheimer approximation[11]. (e) Excited states at the T_1 optimized structure for the strong dimer. (f) Electron density difference of T_1-S_0 at the T_1 optimized structure for the strong dimer. (isosurface value: 5.0×10^{-4} a.u.). (a. u. = atomic units)

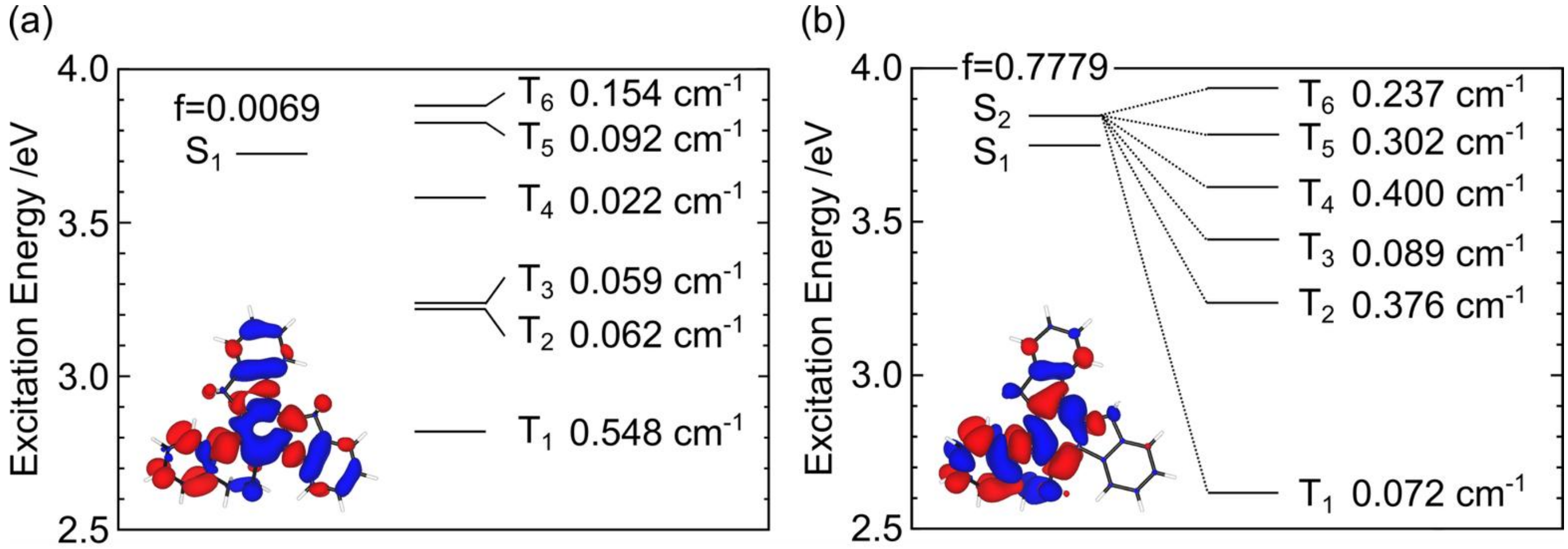

Figure S32. Excited states of MHTX calculated using the PBE0/def2-TZVP method with GD3BJ. Energy levels of excited states at the (a) S_1 and (b) S_2 optimized structures of MHTX, calculated using the PBE0/def2-TZVP method with GD3BJ. Values represent the SOC matrix elements between singlets and triplets. Inset shows the electron density difference of (a) S_1-S_0 and (b) S_2-S_0 (isosurface value: 5×10^{-4} a.u.). (a. u. = atomic units)

6 References

1. Pyrko, A. Self-condensation of 6-Methoxytetralone. *ChemInform* **23**, (1992).
2. Williams, P. cis- and trans-Isomerism in 2-(1-indanylidene)indan-1-one. *ChemComm* 719–720 (1967)
3. Sharma, L. K., Kim, K. B. & Elliott, G. I. A selective solvent-free self-condensation of carbonyl compounds utilizing microwave irradiation. *Green Chemistry* **13**, 1546–1549 (2011).
4. Mueller, A. & Amsharov, K. Y. Synthesis of Robust Precursors for the Controlled Fabrication of (6,6), (8,8), (10,10), and (12,12) Armchair Single-Walled Carbon Nanotubes. *European J. Org. Chem.* **2015**, 3053–3056 (2015).
5. Amick, A. W. & Scott, L. T. Trisannulated Benzene Derivatives by Acid Catalyzed Aldol Cyclotrimerizations of Cyclic Ketones. Methodology Development and Mechanistic Insight. *J. Org. Chem.* **72**, 3412–3418 (2007).
6. Salla, C. A. M. *et al.* Persistent Solid-State Phosphorescence and Delayed Fluorescence at Room Temperature by a Twisted Hydrocarbon. *Angew. Chem. Int. Ed.* **58**, 6982–6986 (2019).
7. Jiang, H. *et al.* Effect of photo-oxidation on the emissive properties of truxene. *J. Photochem. Photobiol. A Chem.* **369**, 195–201 (2019).
8. Chen, F. *et al.* Density, viscosity, speed of sound, excess property and bulk modulus of binary mixtures of γ-butyrolactone with acetonitrile, dimethyl carbonate, and tetrahydrofuran at temperatures (293.15 to 333.15) K. *J. Mol. Liq.* **209**, 683–692 (2015).
9. Sengers, J. V. & Watson, J. T. R. Improved International Formulations for the Viscosity and Thermal Conductivity of Water Substance. *J. Phys. Chem. Ref. Data* **15**, 1291–1314 (1986).
10. Ota, W., Uejima, M. & Sato, T. Role of Vibronic Couplings and Energy Gap in the Internal Conversion Process of a Molecule. *Bull. Chem. Soc. Jpn.* **96**, 582–590 (2023).
11. Santoro, F., Improta, R., Lami, A., Bloino, J. & Barone, V. Effective method to compute Franck-Condon integrals for optical spectra of large molecules in solution. *J. Chem. Phys.* **126**, (2007).
