# Supplementary material for: Anti-Kasha fluorescence and persistent room-temperature phosphorescence from a nearly flat non-symmetric polycyclic hydrocarbon
Source: Nat Commun. 2026 Oct 2;17:11300. doi: 10.1038/s41467-026-78107-6 (PMC13724719; doi:10.1038/s41467-026-78107-6)
Supplement: Supplementary file 2 [file 41467_2026_78107_MOESM2_ESM.pdf]

nature portfolio

Peer Review File

Anti-Kasha fluorescence and persistent room-temperature phosphorescence from a nearly flat non-symmetric polycyclic hydrocarbon

Corresponding Author: Dr Eduard Westphal

This file contains all reviewer reports in order by version, followed by all author rebuttals in order by version.

Attachments originally included by the reviewers as part of their assessment can be found at the end of this file.

Version 0:

Reviewer comments:

Reviewer #1

(Remarks to the Author)
In this manuscript Westphal and coworkers describe the synthesis and thorough optophysical investigation of a monohomotruxene, a compound in which one five-membered ring of truxene is replaced with a six-membered ring. This symmetry-breaking modification was expected to lead to beneficial changes in the optophysical properties.
Especially the optophysical investigations are very extensive and yielded a number of very interesting results, inter alia, the observation of an excitation-dependent dual emission and a very long-lasting phosphorescence. These investigations were performed in solution and in the aggregated state.
I am convinced that these results are of great use for the field and will find the interest of the broad readership of Nature Communications. I recommend publication of the manuscript after minor revisions.
This manuscript is very well written, it needs only negligible language polishing.
Comments:
1) page 2, top line: "However, metal-based phosphors …" I am not aware that compounds showing phosphorescence are generally termed as phosphors. A confusion with the element phosphor should be avoided.
Similarly, an abbreviation of phosphorescence with Ph is not recommended to avoid a confusion with phenyl. I recommend to use the abbreviation Phos or phos.
2) The authors use B3LYP/6-31G(d/p) for calculations, which has for a long time been the standard for quantum-chemical calculations. This functional/level seems to be somewhat outdated. Especially the utilization of dispersion correction (not only for dimer calculations) is now considered to be mandatory. I recommend to read Bursch, M.; Mewes, J.-M.; Hansen, A.; Grimme, S. Best-Practice DFT Protocols for Basic Molecular Computational Chemistry. Angew. Chem. Int. Ed. 2022, 61 (42), e202205735, DOI: 10.1002/anie.202205735.
Grimme and coworkers recommend to use pbe0/def2-TZVP with Grimme's dispersion correction at the D3 level and with Becke-Johnson damping (GD3BJ) for standard calculations, which gives good results at relatively low costs.
I leave it to the authors' decision, to use this for future investigations.
3) Typos: page 2, paragraph 2, last line: coupling *via* out-of-plane modes; Page 4, paragraph 1,line 6: *355 nm results*.

Reviewer #2

(Remarks to the Author)
Westphal et al. report a monohomotruxene (MHTX), an asymmetric polycyclic aromatic hydrocarbon (PAH) rationally designed by lowering the molecular symmetry of homotruxene (HTX) and truxene (TX). MHTX exhibits an unusual anti-Kasha behavior with excitation-wavelength-dependent dual emission, affording an overall higher photoluminescence quantum yield (PLQY) upon higher-energy excitation relative to direct S_1 excitation. Under anaerobic environments, populating the S_2 state generates intense ultralong room-temperature phosphorescence (RTP) spanning 455–480 nm with a persistent lifetime exceeding 12 s at ambient temperature. These observations render MHTX a promising new emissive skeleton and highlight the great capability of asymmetric PAH structures to realize intricate excited-state behaviors and advanced photophysical properties. I would like to suggest for publication of this manuscript after the questions and

suggestions shown below have been taken into consideration.

1. Additional emission-wavelength-dependent excitation experiments should be supplemented. Supplementary lifetime data of RTP and delayed fluorescence should be provided.
2. Could the authors offer additional experimental evidence confirming the $S_4 \rightarrow S_0$ radiative transition?
3. Please check whether the figure correspondence here is correct. In solution, excitation at the edge of the strong absorption region (Ex = 300 nm) yields a vibrationally structured emission band with maxima at 350 and 360 nm and shoulders at 380, 400 and 425 nm (Figure 2a, blue line, and Extended Figure 1a).
4. The authors are suggested to summarize the lifetime parameters of all compounds in a table to facilitate readers' reading and comparative analysis.
5.The authors claim that S_2-state excitation under anaerobic conditions affords strong ultralong room-temperature phosphorescence (RTP) spanning 455–480 nm with a lifetime over 12 s at room temperature, described as the longest phosphorescence among hydrocarbons. Plenty of published works have presented ultralong RTP materials possessing afterglow lifetimes exceeding 12 s, so this sentence requires revision.
6.With regard to the phosphorescence and delayed fluorescence properties of these compounds, the authors may supplement their applications in bioimaging, anti-counterfeiting and other related fields.
7.The authors are suggested to add more recent references focused on anti-Kasha emission and room-temperature phosphorescence, for example: Org. Lett. 2026, 28, 6589-6594. J. Am. Chem. Soc. 2025, 147, 30390–30400. Adv. Funct. Mater. 2025, 35, 2413609. J. Am. Chem. Soc. 2025, 147, 39879-39889. Chem. Sci. 2025, 16, 3107-3113. Chem. Sci., 2025,16, 12051-12057. Chem. Sci., 2025,16, 6755-6762. Chem. Sci., 2024, 15, 12270–12276.

Reviewer #3

(Remarks to the Author)
The manuscript reports the synthesis and photophysical characterization of a novel molecule incorporating structural motifs of truxene and homotruxene. The system exhibits excitation-dependent dual emission, which represents a significant observation in the context of anti-Kasha photophysics and multifunctional organic emitters. In addition, the material displays intense and ultralong room-temperature emission, further underscoring its potential relevance. The complex photophysical processes are reasonably well explained, and overall the topic is timely and of clear interest to the field of photophysics and organic luminescent materials. However, some issues must be addressed before the manuscript can be considered for publication in a high-impact journal such as Nature Communications.

1. To more convincingly demonstrate the advantages of the proposed molecular design, control experiments employing truxene and monohomotruxene would be highly valuable. These compounds have indeed been synthesized and partially studied, however, while some of their photophysical properties are discussed, a more systematic investigation is still needed. In particular, equivalent experiments conducted under identical or closely comparable conditions would enable a more rigorous evaluation of structure–property relationships. A comparative table summarizing the key photophysical parameters would be especially useful to clearly delineate the specific contribution of the hybrid framework.

2. Regarding the aggregation behavior in THF–water mixtures and its interpretation, several concerns arise. The authors attribute the observed dual emission exclusively to a monomeric species based on the apparent invariance of the spectra at low water fractions. However, the emergence of weak intermolecular dimers at higher water fractions ($f_w \geq 70\%$) suggests that intermolecular interactions may already play a subtle role even at lower water contents. The absence of significant spectral shifts alone does not rigorously exclude the presence of weakly bound aggregates or equilibria between multiple emissive species. Furthermore, the decrease of S_2 emission and the concurrent enhancement of S_1 emission when exciting at 300nm at high water fractions also attributed to the formation of weakly interacting dimers is insufficiently explained. It is not clear why the formation of such dimers would selectively suppress S_2 emission while enhancing S_1 emission. A more detailed discussion of how intermolecular interactions influence the excited-state dynamics is required. Although some additional discussion is provided in the Supporting Information, it does not significantly clarify these issues. In fact, Figure S17 appears to be incomplete or inconsistent with its caption.

3. A similar issue arises for the so-called "strong aggregates" observed in 1 wt% drop-cast films in Zeonex. In this case, excitation spectra corresponding to the strongly aggregated species are presented but not sufficiently discussed. A more thorough analysis of these excitation features is encouraged, together with clarification of whether multiple excited states contribute to the observed emission, particularly in the aggregated regime.

4. Finally, the computational approach used to rationalize the behavior of the aggregates should be clarified. It is not clearly stated whether the dimer structures were fully optimized in the excited state, which is a critical point, as excimer formation is inherently an excited-state phenomenon that depends on structural relaxation following excitation. Without explicit evidence of such calculations, the assignment of excimer emission remains uncertain and should therefore be treated with caution.

Version 1:

Reviewer comments:

Reviewer #1

(Remarks to the Author)
My concerns have been addressed. I recommend to publish this manuscript.

Reviewer #2

(Remarks to the Author)
The author adequately addresses the issues of concern, and I recommend publication.

Reviewer #3

(Remarks to the Author)
The authors have satisfactorily responded to my comments and concerns. I believe the manuscript is now suitable for publication

Open Access This Peer Review File is licensed under a Creative Commons Attribution 4.0 International License, which permits use, sharing, adaptation, distribution and reproduction in any medium or format, as long as you give appropriate credit to the original author(s) and the source, provide a link to the Creative Commons license, and indicate if changes were made.
In cases where reviewers are anonymous, credit should be given to 'Anonymous Referee' and the source.
The images or other third party material in this Peer Review File are included in the article's Creative Commons license, unless indicated otherwise in a credit line to the material. If material is not included in the article's Creative Commons license and your intended use is not permitted by statutory regulation or exceeds the permitted use, you will need to obtain permission directly from the copyright holder.
To view a copy of this license, visit https://creativecommons.org/licenses/by/4.0/

Anti-Kasha fluorescence, persistent room-temperature phosphorescence and delayed emission from a nearly flat non-symmetric polycyclic hydrocarbon

(Submitted to Nature Communications – Manuscript NCOMMS-26-047766-T)

We would like to thank the reviewers for their thorough assessment and for the constructive and overall very positive comments they provided.

We have carefully considered all comments and recommendations, and revised the manuscript accordingly. All modifications have been highlighted in yellow in the revised version of the manuscript, and a detailed point-by-point response to the reviewers' comments is provided below, with our responses indicated in blue.

We believe that the revisions have significantly strengthened the manuscript, and we hope that the new version will now be suitable for publication in *Nature Communications*.

Thank you for your time and consideration.

Best regards,
Eduard Westphal

Response to Reviewers

Reviewer #1: In this manuscript Westphal and coworkers describe the synthesis and thorough optophysical investigation of a monohomotruxene, a compound in which one five-membered ring of truxene is replaced with a six-membered ring. This symmetry-breaking modification was expected to lead to beneficial changes in the optophysical properties.
Especially the optophysical investigations are very extensive and yielded a number of very interesting results, inter alia, the observation of an excitation-dependent dual emission and a very long-lasting phosphorescence. These investigations were performed in solution and in the aggregated state.
I am convinced that these results are of great use for the field and will find the interest of the broad readership of Nature Communications. I recommend publication of the manuscript after minor revisions.
We thank the reviewer for the very kind evaluation.

This manuscript is very well written, it needs only negligible language polishing:

1. page 2, top line: "However, metal-based phosphors …" I am not aware that compounds showing phosphorescence are generally termed as phosphors. A confusion with the element phosphor should be avoided.

Similarly, an abbreviation of phosphorescence with Ph is not recommended to avoid a confusion with phenyl. I recommend to use the abbreviation Phos or phos.
R: We agree with the reviewer that the term phosphor may lead to misunderstanding in our introduction. We have replaced it with ‘phosphorescent materials’. Additionally, phosphorescence is now abbreviated as ‘phos’ instead of ‘Ph’ along the text, and on Figures 4, 6, S16, S19 and S20, to enhance the readability of the manuscript.

2. The authors use B3LYP/6-31G(d/p) for calculations, which has for a long time been the standard for quantum-chemical calculations. This functional/level seems to be somewhat outdated. Especially the utilization of dispersion correction (not only for dimer calculations) is now considered to be mandatory. I recommend to read Bursch, M.; Mewes, J.-M.; Hansen, A.; Grimme, S. Best-Practice DFT Protocols for Basic Molecular Computational Chemistry. Angew. Chem. Int. Ed. 2022, 61 (42), e202205735, DOI: 10.1002/anie.202205735.
Grimme and coworkers recommend to use pbe0/def2-TZVP with Grimme’s dispersion correction at the D3 level and with Becke-Johnson damping (GD3BJ) for standard calculations, which gives good results at relatively low costs.
I leave it to the authors’ decision, to use this for future investigations.
R: For a consistency check, we recomputed the excited states of the monomer at the S_1 and S_2 optimized geometries using the suggested method (PBE0/def2-TZVP with GD3BJ), which provided results very comparable to those obtained at the B3LYP/6-31G(d,p) level (Fig. S29). Thus for this work the older B3LYP functional is sufficient. In any case, we thank the reviewer for this valuable suggestion. We have an ongoing collaboration with Dr. Jan Mewes, and we will certainly consider employing the recommended PBE0 method in our future studies, exploring this aspect further with his valuable input in future studies.

3. Typos: page 2, paragraph 2, last line: coupling *via* out-of-plane modes; Page 4, paragraph 1,line 6: *355 nm results*.
R: We thank the reviewer for the observation. The typos have been fixed on the revised version of the manuscript.

<u>Reviewer #2</u>: Westphal et al. report a monohomotruxene (MHTX), an asymmetric polycyclic aromatic hydrocarbon (PAH) rationally designed by lowering the molecular symmetry of homotruxene (HTX) and truxene (TX). MHTX exhibits an unusual anti-Kasha behavior with excitation-wavelength-dependent dual emission, affording an overall higher photoluminescence quantum yield (PLQY) upon higher-energy excitation relative to direct S_1 excitation. Under anaerobic environments, populating the S_2 state generates intense ultralong room-temperature phosphorescence (RTP) spanning 455–480 nm with a persistent lifetime exceeding 12 s at ambient temperature. These observations render MHTX a promising new emissive skeleton and highlight the great capability of asymmetric PAH structures to realize intricate excited-state behaviors and advanced photophysical properties. I would like to suggest for publication of this manuscript after the questions and suggestions shown below have been taken into consideration.
We thank the reviewer for the positive assessment.

1. Additional emission-wavelength-dependent excitation experiments should be supplemented. Supplementary lifetime data of RTP and delayed fluorescence should be provided.

R: We have recorded the excitation profile of MHTX in zeonex films with collection at several wavelengths, in 15 nm steps (see figure below). To avoid presenting the data in a cluttered plot which is difficult to follow, we have only shown the curves collected at 365 nm, 410 nm and 500 nm in the manuscript (Figure 2d), since they comprehensively show the excitation regimes of the $S_0 \rightarrow S_1$ and $S_0 \rightarrow S_2$ transitions and the ground state dimer absorption, as explained in the manuscript. Additionally, we have provided the excitation profile of MHTX in dilute THF solution with different fractions of water in Figure S17. Therefore, we believe that we have found a good compromise to provide sufficient yet still comprehensive experimental excitation data in our manuscript in order to corroborate with our conclusions.

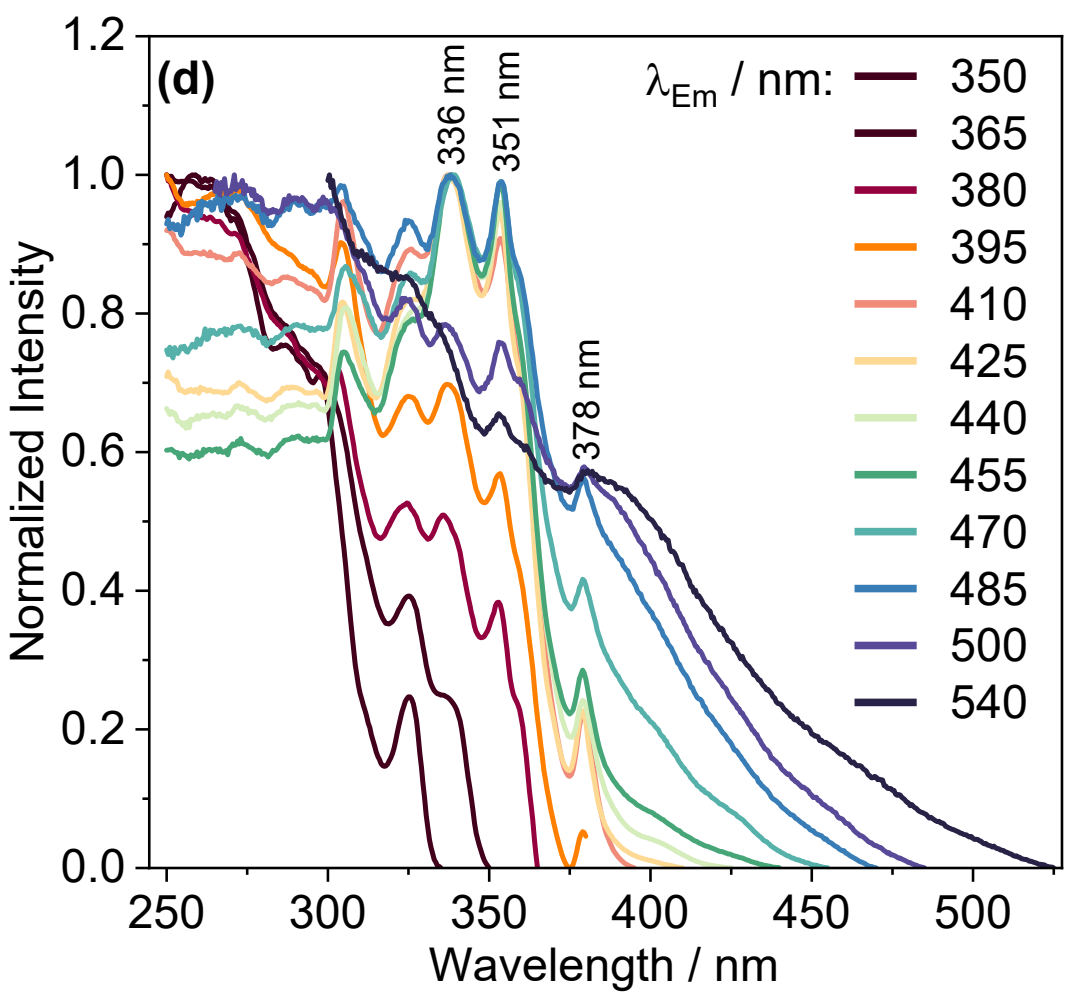

Figure 1 – Normalized excitation profile of a 1 wt.% drop-cast film in Zeonex under collected in different wavelengths.

Regarding the lifetime data of RTP, despite our efforts, we were not able to obtain a sensible RTP decay curve due to limitations in our time-resolved system, i.e. our lowest laser repetition rate is 1 Hz. Therefore, our approach was to report the afterglow duration, which is widely reported in the literature for other ultra-long phosphorescent systems. Moreover, our emphasis here is on the duration of the persistent RTP, not on its dynamics. However, we were able to collect an aggregate delayed fluorescence decay curve from MHTX doped into zeonex matrix at room temperature. This additional data was added to the revised Supplementary Information (Figure S14c), depicting an aggregate τ_{TTA} = 213 µs. Finally, since the phosphorescence is, expectedly, stronger at 80 K, a decay curve was collected (Figure 4d), even though the measurement window of 11 s does not allow capturing the full decay.

2. Could the authors offer additional experimental evidence confirming the $S_4 \rightarrow S_0$ radiative transition?

R: While the intense absorption band below 300 nm, present in Figure 2a and in the theoretical calculations, have partially been attributed to a $S_0 \rightarrow S_4$ transition, there is no mention of a $S_0 \leftarrow S_4$ radiative decay in the manuscript. In case this is a typo and the reviewer was refering to '$S_0 \leftarrow S_2$' instead, we have attributed the high-energy prompt fluorescence (blue curve on Figure 2b) to an anti-Kasha emission from S_2 due to the observation of a lower-energy band (orange curve) and to the excitation dependence of the fluorescence. Additionally, this assignement is backed by the theoretical calculations, as thoroughly explained in the manuscript.

3. Please check whether the figure correspondence here is correct. In solution, excitation at the

edge of the strong absorption region (λEx = 300 nm) yields a vibrationally structured emission band with maxima at 350 and 360 nm and shoulders at 380, 400 and 425 nm (Figure 2a, blue line, and Extended Figure 1a).

R: We thank the reviewers for the observation. The correct correspondence is to Figure 2b, instead of 2a. This has been fixed in the revised version of the manuscript.

4. The authors are suggested to summarize the lifetime parameters of all compounds in a table to facilitate readers' reading and comparative analysis.

R: We thank the reviewer for this excellent suggestion. A compendium table summarizing the extensive lifetime data has been included in the Supplementary Information, Pg. 20 (Table S3).

5. The authors claim that S_2-state excitation under anaerobic conditions affords strong ultralong room-temperature phosphorescence (RTP) spanning 455–480 nm with a lifetime over 12 s at room temperature, described as the longest phosphorescence among hydrocarbons. Plenty of published works have presented ultralong RTP materials possessing afterglow lifetimes exceeding 12 s, so this sentence requires revision.

R: We agree with the reviewers comment. Longer-lived RTP has been reported for other hydrocarbons, such as coronene and chrysene. Therefore, we made the following change to the sentence:

"To our knowledge, this is <u>amongst</u> the longest blue phosphorescence lifetimes observed with PAHs."

We would like to reiterate that monohomotruxene exhibits 12 s of afterglow of blue phosphorescence and that no rigidity-enhancing approaches were employed for reaching this ultra-long RTP, highlighting the relevance of our findings.

6. With regard to the phosphorescence and delayed fluorescence properties of these compounds, the authors may supplement their applications in bioimaging, anti-counterfeiting and other related fields.

R: We thank the reviewer for the suggestion on futher elaborating on the potential application of MHTX. Accordingly, these applications have been mentioned in the Introduction, and the following sentence has been added to the Discussion section (Pg 11):

"Persistent phosphorescence linked to dual fluorescence holds potential for the application in the development of anti-counterfeiting techniques."

7. The authors are suggested to add more recent references focused on anti-Kasha emission and room-temperature phosphorescence, for example: Org. Lett. 2026, 28, 6589-6594. J. Am. Chem. Soc. 2025, 147, 30390−30400. Adv. Funct. Mater. 2025, 35, 2413609. J. Am. Chem. Soc. 2025, 147, 39879-39889. Chem. Sci. 2025, 16, 3107-3113. Chem. Sci., 2025,16, 12051-12057. Chem. Sci., 2025,16, 6755-6762. Chem. Sci., 2024, 15, 12270−12276.

R: We thank the reviewer for the suggestion. The following most relavant and comprehensive reference to this work has been added:

Wan, L., Ling, S., Yang, L. & Li, B. Tuning the energetics of carbonyl-bridged triarylamines: from thermally activated delayed fluorescence to anti-Kasha dual-emission and room temperature phosphorescence materials. *Chem. Sci.* **16**, 12051–12057 (2025).

<u>Reviewer #3</u>: The manuscript reports the synthesis and photophysical characterization of a novel molecule incorporating structural motifs of truxene and homotruxene. The system

exhibits excitation-dependent dual emission, which represents a significant observation in the context of anti-Kasha photophysics and multifunctional organic emitters. In addition, the material displays intense and ultralong room-temperature emission, further underscoring its potential relevance. The complex photophysical processes are reasonably well explained, and overall the topic is timely and of clear interest to the field of photophysics and organic luminescent materials. However, some issues must be addressed before the manuscript can be considered for publication in a high-impact journal such as Nature Communications.

1. To more convincingly demonstrate the advantages of the proposed molecular design, control experiments employing truxene and monohomotruxene would be highly valuable. These compounds have indeed been synthesized and partially studied, however, while some of their photophysical properties are discussed, a more systematic investigation is still needed. In particular, equivalent experiments conducted under identical or closely comparable conditions would enable a more rigorous evaluation of structure–property relationships. A comparative table summarizing the key photophysical parameters would be especially useful to clearly delineate the specific contribution of the hybrid framework.
R: We thank the reviewer for the comment. We understand that the next step from the work presented in this manuscript is to do a systematic comparison between truxene, homotruxene and monohomotruxene. We are currently working on this investigation in view of a next contribution on the topic. However, this will be a very extensive and detailed work in its own right, and will necessarily have to be published separately. Including such results in the present manuscript would considerably expand its scope, making it unsuitable for a Communication, whereas the primary focus and novelty of the current work lie in the presentation of monohomotruxene. However, we have added some initial comparison results that are relatively simple to understand and highlight the advantages of monohomotruxene compared to the parent truxene and homotruxene, which add to the messages of this present work without becoming excessively complex and detailed.

2. Regarding the aggregation behavior in THF–water mixtures and its interpretation, several concerns arise. The authors attribute the observed dual emission exclusively to a monomeric species based on the apparent invariance of the spectra at low water fractions. However, the emergence of weak intermolecular dimers at higher water fractions (fw ≥ 70%) suggests that intermolecular interactions may already play a subtle role even at lower water contents. The absence of significant spectral shifts alone does not rigorously exclude the presence of weakly bound aggregates or equilibria between multiple emissive species. Furthermore, the decrease of S_2 emission and the concurrent enhancement of S_1 emission when exciting at 300 nm at high water fractions also attributed to the formation of weakly interacting dimers is insufficiently explained. It is not clear why the formation of such dimers would selectively suppress S_2 emission while enhancing S_1 emission. A more detailed discussion of how intermolecular interactions influence the excited-state dynamics is required. Although some additional discussion is provided in the Supporting Information, it does not significantly clarify these issues. In fact, Figure S17 appears to be incomplete or inconsistent with its caption.
R: At low water fraction, or in pure THF / 2-MeTHF, we have assigned the dual emission to $S_2 \rightarrow S_0$ and $S_1 \rightarrow S_0$ radiative decays (Figure 2b, blue and orange curves, respectively). We do not believe that the low-energy resolved band is attributed to a non-monomeric species in the absence of high water content, considering (i) the dilute concentration of 10 μM used for the solution measurements, (ii) the observations from the absorption spectra and excitation profiles, (iii) the high PLQY of this emission (63%) and (iv) the results from the theoretical calculations. However, we do observe that the peaks of the $S_1 \rightarrow S_0$ undergo a small but clear red-shift of 4 nm at f_w > 60%. (Extended Data Figure 2b), which we then attribute to the formation of weakly bound dimers induced by their low solubility in highly polar

environments. Additionally, we observe the emergence of a weak band between 500-550 nm at f_w>80% (Extended Data Figure 2d), assigned to strong aggregate states, in accordance with the data obtained from 1wt. % zeonex films (Figure 2c, λ_{ex} = 420 nm), where aggregate content is expected to the elevated.

We agree with the reviewer that it is intriguing how S_2 emission is quenched at $f_w > 60\%$, however we do not agree with the statement that S_1 emission in enhanced at high f_w under 300 nm excitation. S_2 emission is lost due to the formation of loose dimers (tentatively through enhanced vibronic coupling assisted internal conversion to the weak dimer intermediate state, Figure S27c), which then emit with 1st peak at 388 nm, a 4 nm red shift compared to the monomeric $S_1 \rightarrow S_0$ emission (Extended Data Figure 2a). The small energy shift caused by the weak dimer interaction indicates that the aggregate states are similar to the monomeric species and that intermolecular interactions are weak. Similar behavior is observed under 355 nm excitation (Extended Data Figure 2b), where directly excited monomeric S_1 fluorescence is quenched and the radiative decay of the loose dimers is observed at $f_w \geq 70\%$. Given that this may not have been explained sufficiently clearly in the manuscript, we have reformulated part of the 3rd paragraph on Pg. 4 to the following to rectify this:

"The dual-emission remained remarkably unchanged at $f_w \leq 60\%$, indicating a strong THF–**MHTX** interaction that forms a protective solvation shell, effectively hindering molecular aggregation despite the increasing presence of water. In contrast, at $f_w \geq 70\%$, the S_2 emission strongly decreases, and an emission band similar to S_1 emission appears when exciting at 300 nm (into S_2), however the peaks are red-shifted by 4 nm (0.03 eV). Exciting into S_1 (355 nm) gives very weak S_1 emission from 0 to 70% water content, followed by a 4 nm (0.03 eV) red-shift of the band at higher water fractions. This coincides perfectly with the emergence of the strong S_1 emission and decline of S_2 emission when exciting at 300 nm. We suggest this red-shifted band emerges due to the formation of a weak intermolecular dimer as the water content starts to overcome the stabilizing THF solvation shell, where the molecular orbitals remain predominantly localized on individual molecules and intermolecular interactions are weak."

Finally, we thank the reviewer for the observation that the caption of Figure S17 is incorrect. This has been corrected in the revised version of the manuscript.

3. A similar issue arises for the so-called "strong aggregates" observed in 1 wt% drop-cast films in Zeonex. In this case, excitation spectra corresponding to the strongly aggregated species are presented but not sufficiently discussed. A more thorough analysis of these excitation features is encouraged, together with clarification of whether multiple excited states contribute to the observed emission, particularly in the aggregated regime.
R: We agree with the reviewer that the excitation profiles depicted in Figure 2d are not satisfactorily discussed in the manuscript. We observe that a broad, low-energy excitation band appears around 400 nm when emission from the strong aggregate is collected, at 500 nm. Moreover, we would like to reiterate how the excitation profile of the strong aggregate also shows contribution from $S_0 \rightarrow S_1$ and $S_0 \rightarrow S_2$ absorptions, which indicates that there is energy transfer (possibly FRET) from the monomeric excited states to the aggregate. This process could be mediated by the intermediate states of the aggregate, which are closer in energy to S_1 and S_2. Therefore, we have included the following sentence to the paragraph in Pg. 5:

"A broad, low-energy excitation band associated with this non-monomeric species appears around 400 nm (λ_{Em} = 500 nm, Figure 2d), confirming that it has a ground state and is, indeed, an aggregate and not an excimer. Additionally, sharp excitation features associated with $S_0 \rightarrow S_1$ (351 and 378 nm) and $S_0 \rightarrow S_2$ (336 nm) are clearly present, as in the absorption spectra in

solution, indicating energy transfer from monomeric excited states to the aggregate, likely through enhanced vibronic coupling assisted internal conversion to the aggregate intermediate state (Figure S27c)."

Lastly, regarding the origin of the aggregate state emission, theoretical calculations tell us that the radiative decay from both the loose and the strong dimer arises from the lowest-energy excited state from the dimerized species (Figure S27). However, they both have intermediate state that could be pivotal for the energy tranfer between monomer and aggregate, as mentioned above. Additionally, strong dimer phosphorescence also has a single origin at the lowest excited state T_1 (Figure S28).

4. Finally, the computational approach used to rationalize the behavior of the aggregates should be clarified. It is not clearly stated whether the dimer structures were fully optimized in the excited state, which is a critical point, as excimer formation is inherently an excited-state phenomenon that depends on structural relaxation following excitation. Without explicit evidence of such calculations, the assignment of excimer emission remains uncertain and should therefore be treated with caution.
R: Thank you for the comment. We fully optimized the excited-state geometries of the dimer species and confirmed their stability through vibrational frequency analysis. We have added this explanation to the Methods section, Pg 12:

"The excited-state geometries of the monomer and dimer were fully optimized, and their stability was confirmed by vibrational frequency analysis. The S_1 and S_2 excited states of the monomer were also computed using a more recent method, PBE0/def2-TZVP with GD3BJ [40], yielding results comparable to those obtained at the B3LYP/6-31G(d,p) level (Fig. S29); therefore, B3LYP/6-31G(d,p) was employed in the present study."

Further modifications:

- The abstract and the manuscript have been revised and reformatted in accordance with the requirements and recommendations of *Nature Communications*.

- We have noticed the lack of introduction to the topic of anti-Kasha emission during the revision process, therefore, the following short paragraph has been added to the introduction section, Pg. 2:

  "Another photophysical process with important applications is emission from multiple singlet excited states, yielding excitation-dependent multilevel emission with potential uses in sensing, bioimaging, and anti-counterfeiting. This phenomenon represents a violation of Kasha's rule, which states that radiative decay occurs exclusively from the lowest-energy excited electronic state. However, as observed in anti-Kasha emitters such as azulene and pyrene, $S_2 \rightarrow S_0$ emission can occur when the rate of $S_2 \rightarrow S_1$ internal conversion (IC) becomes comparable to the fluorescence rate for $S_2 \rightarrow S_0$ radiative decay."

- Reformulation of Line 3, Pg. 2, paragraph 6:

  "In this work, we demonstrate how enhancing molecular planarity and rigidity, and

reducing the symmetry from **HTX** can preserve TTA and RTP, enhance the photoluminescence quantum yield (PLQY) and result in complex photophysic behaviour, with radiative decay from upper excited states.”

- The word "light" was corrected in Figure 4e.

- The following references have been added:

Lawrance, R. *et al.* From Kasha to Anti-Kasha: Nanomaterials Unlock Applications. *ACS Applied Optical Materials* (2026)

Wan, L., Ling, S., Yang, L. & Li, B. Tuning the energetics of carbonyl-bridged triarylamines: from thermally activated delayed fluorescence to anti-Kasha dual-emission and room temperature phosphorescence materials. *Chem. Sci.* **16**, 12051–12057 (2025).

Wan, L., Ling, S., Yang, L. & Li, B. Tuning the energetics of carbonyl-bridged triarylamines: from thermally activated delayed fluorescence to anti-Kasha dual-emission and room temperature phosphorescence materials. *Chem. Sci.* **16**, 12051–12057 (2025).

You, Z. L. *et al.* Spatiotemporally Resolved Anti-Counterfeiting via Multicomponent Zero-Dimensional Metal Halides with Anti-Kasha Emission. *Adv. Opt. Mater.* **14**, e02654 (2025).

Franca, L. G., Long, Y., Li, C., Danos, A. & Monkman, A. The Critical Role of $n\pi^*$ States in the Photophysics and Thermally Activated Delayed Fluorescence of Spiro Acridine-Anthracenone. *J. Phys. Chem. Lett.* **12**, 1490–1500 (2021).

Barman, D. *et al.* Anti-Kasha emissive organic materials: molecular design, fundamental strategies, mechanistic insights, and emerging applications. *Physical Chemistry Chemical Physics* (2026).

Kuang, Q., Hou, X., Du, C., Wang, X. & Gao, D. Recent advances in the anti-counterfeiting applications of long persistent phosphors. *Physical Chemistry Chemical Physics* **25**, 17759–17768 (2023).

Bursch, M., Mewes, J.-M., Hansen, A. & Grimme, S. Best-Practice DFT Protocols for Basic Molecular Computational Chemistry. *Angew. Chem. Int. Ed.* **61**, e202205735 (2022).

Westphal et al. report a monohomotruxene (MHTX), an asymmetric polycyclic aromatic hydrocarbon (PAH) rationally designed by lowering the molecular symmetry of homotruxene (HTX) and truxene (TX). MHTX exhibits an unusual anti-Kasha behavior with excitation-wavelength-dependent dual emission, affording an overall higher photoluminescence quantum yield (PLQY) upon higher-energy excitation relative to direct S_1 excitation. Under anaerobic environments, populating the S_2 state generates intense ultralong room-temperature phosphorescence (RTP) spanning 455–480 nm with a persistent lifetime exceeding 12 s at ambient temperature. These observations render MHTX a promising new emissive skeleton and highlight the great capability of asymmetric PAH structures to realize intricate excited-state behaviors and advanced photophysical properties. I would like to suggest for publication of this manuscript after the questions and suggestions shown below have been taken into consideration.

1. Additional emission-wavelength-dependent excitation experiments should be supplemented. Supplementary lifetime data of RTP and delayed fluorescence should be provided.
2. Could the authors offer additional experimental evidence confirming the $S_4 \rightarrow S_0$ radiative transition?
3. Please check whether the figure correspondence here is correct. In solution, excitation at the edge of the strong absorption region (λ_{Ex} = 300 nm) yields a vibrationally structured emission band with maxima at 350 and 360 nm and shoulders at 380, 400 and 425 nm (Figure 2a, blue line, and Extended Figure 1a).
4. The authors are suggested to summarize the lifetime parameters of all compounds in a table to facilitate readers' reading and comparative analysis.

5． The authors claim that S_2-state excitation under anaerobic conditions affords strong ultralong room-temperature phosphorescence (RTP) spanning 455–480 nm with a lifetime over 12 s at room temperature, described as the longest phosphorescence among hydrocarbons. Plenty of published works have presented ultralong RTP materials possessing afterglow lifetimes exceeding 12 s, so this sentence requires revision.

6. With regard to the phosphorescence and delayed fluorescence properties of these compounds, the authors may supplement their applications in bioimaging, anti-counterfeiting and other related fields.

7. The authors are suggested to add more recent references focused on anti-Kasha emission and room-temperature phosphorescence, for example: *Org. Lett.* **2026**, *28*, 6589-6594. *J. Am. Chem. Soc.* **2025**, *147*, 30390−30400. *Adv. Funct. Mater.* **2025**, *35*, 2413609. *J. Am. Chem. Soc.* **2025**, *147*, 39879-39889. *Chem. Sci*. **2025**, *16*, 3107-3113. *Chem. Sci.*, **2025**,*16*, 12051-12057. *Chem. Sci.*, **2025**,*16*, 6755-6762. *Chem. Sci.*, **2024**, *15*, 12270−12276.
